# Sinapic Acid Esters: Octinoxate Substitutes Combining Suitable UV Protection and Antioxidant Activity

**DOI:** 10.3390/antiox9090782

**Published:** 2020-08-24

**Authors:** Cédric Peyrot, Matthieu M. Mention, Fanny Brunissen, Florent Allais

**Affiliations:** URD Agro-Biotechnologies Industrielles (ABI), CEBB, AgroParisTech, 51110 Pomacle, France; cedric.peyrot@agroparistech.fr (C.P.); matthieu.mention@agroparistech.fr (M.M.M.); fanny.brunissen@agroparistech.fr (F.B.)

**Keywords:** organic UV filters, antioxidant, sinapic acid esters, photoprotection, bio-based chemicals

## Abstract

In 2021, Hawaii will permanently ban the use and sale of octinoxate-based sunscreens as studies have shown serious impacts of such UV filters on the coral reef. This ban, which could be generalized to other countries, highlights the extreme need to offer alternative UV filters that are not only effective in terms of sun protection, but also healthy with regards to human health and the environment. In this context, a wide library of *p*-hydroxycinnamic esters deriving from naturally occurring sinapic acid has been synthesized using a Knoevenagel–Doebner condensation. The UV filtering activities as well as the antioxidant properties of these sinapic acid esters were then investigated. The results showed promising UVB protection and antioxidant efficacy. A Structure–Activity Relationship (SAR) study on the sinapic acid esters highlighted the need of a free phenol to, as expected, observe antioxidant activity, but also to obtain a higher intensity of protection. Moreover, the nature of the ester moiety also proved to be a key structural feature for the UV absorbance, as higher steric hindrance on the ester moiety leads to more active compounds. The judicious structural design of sinapic esters thus provides promising compounds combining UV protection and antioxidant activity.

## 1. Introduction

A high level of sun exposure can lead to sunburn, premature skin aging and, in the worst case, severe forms of skin cancer [1,2,3]. To offset those negative effects, the use of UV protection is essential. There are two types of commonly used UV filters: mineral filters (TiO_2_, ZnO) and organic filters (avobenzone, octocrylene or octinoxate) [4,5]. The latter is a UVB filter commonly found in sunscreen lotions used on beaches worldwide. However, in recent years, octinoxate has been strongly criticized, in particular for its negative impact on the environment but also on human health [6,7]. Indeed, several studies have demonstrated that organic UV filters induce a virus proliferation which is the source of coral reef bleaching [8,9]. To protect its coral reefs, Hawaii has notably banned the use and sale of sunscreen products containing octinoxate on its territory, starting January 2021 [10]. Such a drastic decision could be taken by many other states in the coming years. Moreover, octinoxate is actively challenged for its endocrine disrupting activity, which can have serious effects on humans [11]. Considering the above considerations, designing new safer alternatives to current UVB filters remains a challenge.

From a synthetic point of view, octinoxate also presents many disadvantages. Indeed, this molecule, produced on a very large scale, is obtained from non-renewable fossil resources. Several patents and publications offer various synthetic routes [12,13,14,15]; the one mainly used at the industrial scale starts with 4-bromo-anisole and consists of reacting it with 2-ethylhexyl acrylate via a palladium-catalyzed Heck reaction in aprotic polar solvent (NMP, DMF) (yields > 80%) [16,17]. Several optimizations have been proposed, notably the use of reusable ionic liquids as a solvent [18]. Other alternatives starting from renewable resources have been proposed. For instance, *p*-coumaric acid, one of the major lignocellulosic-derived *p*-hydroxycinnamic acids [19], is subjected to methylation then esterification with 2-ethylhexanol [20,21]. It is noteworthy that the esterification can be carried out either chemically or enzymatically with lipases [22,23]. The most-described chemical pathway relies on the formation of the acyl chloride and the subsequent reaction with 2-ethylhexanol [24]. Transesterification of 4-methoxy-ethylcinnamate is also reported in the literature [20,25].

**Figure 1:**
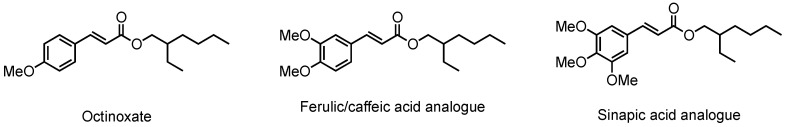
Octinoxate and analogues.

Through the same synthetic pathway, octinoxate analogues were synthesized from ferulic acid [24] and caffeic acid [24], two other naturally occurring *p*-hydroxycinnamic acids. Nevertheless, to the best of our knowledge, no sinapic acid-based analogue has ever been described (Figure 1). This is particularly surprising as it is well known that sinapic acid is the most efficient in terms of absorbance among *p*-hydroxycinnamic acids.

In addition, sinapic acid has already demonstrated its great potential as a starting material for UV filter production [26,27]. Moreover, naturally occurring sinapoyl malate plays the role of a UV filter at the leaves’ surface to protect them from the harmful effects of UV radiation [28]. Several physico-chemical studies have made it possible to better understand the protection mechanism of those molecules [29,30]. Recent work on the highly selective β-β dimerization of sinapic acid esters has highlighted their great potential both as UV filters and antioxidants [26]. Based on these observations, we decided to focus our efforts on the synthesis of renewable sinapic-acid-based octinoxate analogues with dual properties (i.e., UV filter, antioxidant). After having constituted a library of analogues using the well-described pyridine/aniline-mediated Knoevenagel–Doebner condensation as a key step, the influence of the phenol methylation, as well as that of the nature of the ester moiety, on the UV absorbance properties and the antioxidant activity, has been investigated using SARs. Photostability upon UV irradiation was also assessed.

## 2. Materials and Methods

### 2.1. Chemical Synthesis

Syringaldehyde, aniline, pyridine, heptan-1-ol, oleic alcohol, 3,5-dimethylphenol, *o*-cresol, solketal, eugenol, Meldrum’s acid and DPPH were purchased from Sigma Aldrich (St. Louis, MO, USA). Thymol, *p*-hydroxybenzaldehyde, 2-ethylhexan-1-ol, guaiacol and *n*-butylamine were purchased from TCI (Tokyo, Japan). Conc. HCl and solvents were purchased from Fisher Scientific (Hampton, NH, USA) and used as received. All chemicals were used directly without purification.

Chromatographic purifications of products were accomplished using a flash-prep LC system puriFlash^®^ 4100 from Interchim (Montluçon, France) with prepacked silica column (30 μm, Interchim PF-Si30-HP), dual wavelength collection (λ = 254/320 nm) in cyclohexane/ethyl acetate eluent. ^1^H NMR spectra were recorded on a Bruker Fourier 300 (300 MHz) (Bruker, Billerica, MA, USA) and were calibrated with residual DMSO-*d*_6_ or CDCl_3_ protons signals at δ 2.50 or 7.26 ppm, respectively. Data are reported as follows: chemical shift (δ ppm), multiplicity (s = singlet, d = doublet, t = triplet, q = quartet, sept = septet, dd = doublet of doublet, td = triplet of doublet, m = multiplet), coupling constant (Hz), integration and assignment. ^13^C NMR spectra were recorded on a Bruker Fourier 300 (75 MHz) (Bruker, Billerica, MA, USA) and were calibrated with DMSO-*d*_6_ or CDCl_3_ signal at δ 39.52 or 77.16 ppm, respectively. Data are reported as follows: chemical shift (δ ppm) and attribution. ^1^H and ^13^C NMR spectra are available in the ESI. All NMR assignments were made using COSY, HMBC and HSQC spectra. IR spectra were recorded on an Agilent Cary 630 FTIR Spectrometer (Wilmington, DE, USA) and are reported by frequency of absorption (cm^−1^). Melting points were recorded on a Mettler Toledo MP50 Melting Point System (Greifensee, Switzerland) with ME-18552 sample tubes. HRMS were performed on an Agilent 1290 system (Wilmington, DE, USA), equipped with a PDA UV detector, and a 6545 Q-TOF mass spectrometer with a JetStream ESI probe operating at atmospheric pressure as the source.

#### 2.1.1. Malonate Mono-Esters General Procedures

**General Procedure 1 (GP1):** Meldrum’s acid (5.0 g, 35 mmol) and the corresponding alcohol (35 mmol, 1 eq) were melted at 95 °C and stirred for 3 h. After cooling at r.t., the reaction mixture was partitioned between ethyl acetate and saturated aq. NaHCO_3_. The aqueous layer was acidified until pH = 1 with concentrated HCl and extracted with ethyl acetate. The resulting organic layer was dried over anhydrous MgSO_4_, filtered and concentrated.

**General Procedure 2 (GP2):** Meldrum’s acid (4.0 g, 27.8 mmol) and an excess of the corresponding alcohol were heated at reflux overnight. After cooling at r.t., the excess of alcohol was evaporated under reduced pressure.

For each intermediate, yields, NMR analyses and spectra are available in the supplementary information.

#### 2.1.2. Sinapate Esters

The corresponding malonate mono-ester (8.6 mmol), *p*-hydroxybenzaldehyde or syringaldehyde (10.4 mmol, 1.2 eq) and aniline (79 μL, 0.86 mmol) were mixed in pyridine (5.7 mL). The reaction mixture was stirred at 60 °C overnight. After cooling at r.t., the reaction was partitioned between ethyl acetate and 1M aqueous HCl. The organic layer was dried over anhydrous MgSO_4_, filtered and concentrated before being purified by flash chromatography using cyclohexane/ethyl acetate.

**2-Ethylhexyl sinapate (1)** was synthesized as a yellow oil (66% yield) from syringaldehyde and corresponding malonic monoester. Characterization data were identical to those already described. **λ_max_** (EtOH, nm) 332. **ε** (L·mol^−1^·cm^−1^): 19643 [26].

**Ethyl sinapate (4)** was synthesized following procedure from Jaufurally et al. Characterization data were identical to those already described in the literature. **λ_max_** (EtOH, nm) 328. **ε** (L·mol^−1^·cm^−1^): 15015 [31].

***tert*-Butyl sinapate (5)** was synthesized as a pale-yellow powder (60% yield) from syringaldehyde and corresponding malonic monoester. Characterization data were identical to those already described in the literature. **λ_max_** (EtOH, nm) 327. **ε** (L·mol^−1^·cm^−1^): 20847 [26].

**Oleyl sinapate (6)** was synthesized as an orange oil (44% yield) from syringaldehyde and corresponding malonic monoester. **^1^H NMR** (300 MHz, DMSO-*d*_6_): δ (ppm) 8.97 (s, 1H, OH), 7.54 (d, *J* = 15.9 Hz, 1H, H3), 7.02 (s, 2H, H5), 6.52 (d, *J* = 15.9 Hz, 1H, H2), 5.32 (t, *J* = 5.0 Hz, 2H, H17 + H18), 4.11 (t, *J* = 6.5 Hz, 2H, H9), 3.79 (s, 6H, H8), 1.98 (m, 4H, H16 + H19), 1.60 (m, 2H, H10), 1.25 (m, 22H, H11–15 + H20–25), 0.84 (m, 3H, H26). **^13^C NMR** (75 MHz, DMSO-*d*_6_): δ (ppm) 167.1 (C1), 148.5 (C6), 145.7 (C3), 138.7 (C7), 130.1 (C17 + C18), 124.8 (C4), 115.4 (C2), 106.6 (C5), 64.2 (C9), 56.5 (C8), 31.8 (C11–15 or C20–25), 29.0–29.6 (C11–15 or C20–25), 28.8 (C10), 27.0 (C16 + C19), 22.6 (C11–15 or C20–25), 14.4 (C26). **λ_max_** (EtOH, nm) 331. **ε** (L·mol^−1^·cm^−1^): 16,767. **HRMS** (*m/z*) [M + H]^+^ calcd for C_29_H_47_O_5_: 475.3423; found: 475.3417.

***iso*-Propyl sinapate (7)** was synthesized as a pale-yellow oil (73% yield) from syringaldehyde and corresponding malonic monoester. Characterization data were identical to those already described in the literature. **λ_max_** (EtOH, nm) 330. **ε** (L·mol^−1^·cm^−1^): 19584 [32].

**Solketal sinapate (8)** was synthesized as an orange powder (57% yield) from syringaldehyde and corresponding malonic monoester. **^1^H NMR** (300 MHz, CDCl_3_): δ (ppm) 7.62 (d, *J* = 15.8 Hz, 1H, H3), 6.76 (s, 2H, H5), 6.35 (d, *J* = 15.8 Hz, 2H, H2), 4.40 (m, 1H, H10), 4.32 (dd, *J* = 11.5, 4.41 Hz, 1H, H9), 4.18 (dd, *J* = 11.5, 6.3 Hz, 1H, H9), 4.13 (dd, *J* = 8.3, 6.4 Hz, 1H, H11), 3.91 (s, 6H, H8) 3.80 (dd, *J* = 8.4, 6.0 Hz, 1H, H11), 1.46 (s, 3H, H13 or H14), 1.39 (s, 3H, H13 or H14). **^13^C NMR** (75 MHz, CDCl_3_): δ (ppm) 167.0 (C1), 147.3 (C6), 145.9 (C3), 137.3 (C7), 125.9 (C4) 115.3 (C2), 110.1 (C12), 105.2 (C5), 73.9 (C10), 66.5 (C11), 65.0 (C9), 56.4 (C8), 29.9 (C13 or C14), 25.5 (C13 or C14). **λ_max_** (EtOH, nm) 332. **ε** (L·mol^−1^·cm^−1^): 15460. **Mp**: 104–106 °C. **HRMS** (*m/z*) [M + H]^+^ calcd for C_17_H_23_O_7_: 339.1444; found: 339.1452.

**Heptyl sinapate (9)** was synthesized as a brown oil (72% yield) from syringaldehyde and corresponding malonic monoester. Characterization data were identical to those already described in the literature. **λ_max_** (EtOH, nm) 330. **ε** (L·mol^−1^·cm^−1^): 19009 [26].

**Syringol sinapate (10)** was synthesized as a white powder (39% yield) from syringaldehyde and corresponding malonic monoester. **^1^H NMR** (300 MHz, DMSO-*d*_6_): δ (ppm) 9.09 (s, 1H, OH), 7.70 (d, *J* = 15.9 Hz, 1H, H3), 7.18 (t, *J* = 9.0 Hz, 1H, H12), 7.13 (s, 2H, H5), 6.78 (d, *J* = 15.9 Hz, 1H, H2), 6.76 (d, *J* = 9.0 Hz, 2H, H11), 3.81 (s, 6H, H8), 3.75 (s, 6H, H13). **^13^C NMR** (75 MHz, DMSO-*d*_6_): δ (ppm) 164.4 (C1), 152.2 (C10), 148.1 (C6), 147.2 (C3), 138.7 (C7), 128.2 (C9), 126.2 (C12), 124.3 (C4), 113.8 (C2), 106.6 (C5), 105.0 (C11), 56.1 (C8), 56.0 (C13). **λ_max_** (EtOH, nm) 334. **ε** (L·mol^−1^·cm^−1^): 21,458. **Mp**: 169–172 °C. **HRMS** (*m/z*) [M + Na]^+^ calcd for C_19_H_20_O_7_Na: 383.1107; found: 383.1110.

**Guaiacol sinapate (11)** was synthesized as a yellow-orange powder (55% yield) from syringaldehyde and corresponding malonic monoester. Characterization data were identical to those already described in the literature. **λ_max_** (EtOH, nm) 337. **ε** (L·mol^−1^·cm^−1^): 25135 [26].

**Cresol sinapate (12)** was synthesized as yellow powder (63% yield) from syringaldehyde and the corresponding malonic monoester. **^1^H NMR** (300 MHz, DMSO-*d*_6_): δ (ppm) 9.10 (s, 1H, OH), 7.78 (d, *J* = 15.9 Hz, 1H, H3), 7.31 (td, *J* = 7.2, 1.5 Hz, 1H, H13), 7.25 (dd, *J* = 7.6, 1.7 Hz, 1H, H11), 7.20 (dd, *J* = 7.3, 1.5 Hz, 1H, H14), 7.14 (s, 2H, H5), 7.10 (dd, *J* = 7.7, 1.4 Hz, 1H, H12), 6.81 (d, *J* = 15.9 Hz, 1H, H2), 3.82 (s, 6H, H8), 2.14 (s, 3H, H15). **^13^C NMR** (75 MHz, DMSO-*d*_6_): δ (ppm) 165.1 (C1), 149.3 (C9), 148.1 (C6), 147.5 (C3), 138.8 (C7), 131.0 (C13), 130.0 (C10), 127.1 (C11), 125.9 (C14), 124.2 (C4), 122.2 (C12), 113.6 (C2), 106.6 (C5), 56.1 (C8), 15.9 (C15). **λ_max_** (EtOH, nm) 338. **ε** (L·mol^−1^·cm^−1^): 31314 cm^−1^. **Mp**: 120–122 °C. **HRMS** (*m/z*) [M + H]^+^ calcd for C_18_H_19_O_5_: 315.1232; found: 315.1230.

**Thymol sinapate (13)** was synthesized as a white powder (73% yield) from syringaldehyde and corresponding malonic monoester. **^1^H NMR** (300 MHz, DMSO-*d*_6_): δ (ppm) 9.09 (s, 1H, OH), 7.76 (d, *J* = 15.9 Hz, 1H, H3), 7.26 (d, *J* = 7.9 Hz, 1H, H14), 7.14 (s, 2H, H5), 7.06 (dd, *J* = 7.9, 1.1 Hz, 1H, H13), 6.88 (d, *J =* 1.0 Hz, 1H, H11), 6.82 (d, *J* = 15.9 Hz, 1H, H2), 3.82 (s, 6H, H8), 2.94 (sept, *J* = 7.0 Hz, 1H, H15), 2.28 (s, 3H, H17), 1.14 (d, *J* = 6.9 Hz, 6H, H16). **^13^C NMR** (75 MHz, DMSO-*d*_6_): δ (ppm) 166.1 (C1), 148.5 (C6), 148.2 (C9), 147.8 (C3), 139.4 (C7), 137.3 (C10), 136.6 (C12), 127.3 (C13), 126.8 (C14), 124.6 (C4), 123.3 (C11), 114.1 (C2), 107.1 (C5), 56.6 (C8), 27.1 (C15), 23.4 (C16), 20.8 (C17). **λ_max_** (EtOH, nm) 337. **ε** (L·mol^−1^·cm^−1^): 27330. **Mp**: 120–122 °C. **HRMS** (*m/z*) [M + H]^+^ calcd for C_21_H_25_O_5_: 357.1702; found: 357.1701.

**Eugenol sinapate (14)** was synthesized as a yellow powder (48% yield) from syringaldehyde and corresponding malonic monoester. **^1^H NMR** (300 MHz, DMSO-*d*_6_): δ (ppm) 9.07 (s, 1H, OH), 7.71 (d, *J* = 15.8 Hz, 1H, H3), 7.12 (s, 2H, H5), 7.04 (d, *J* = 8.0 Hz, 1H, H14), 6.96 (d, *J* = 1.2 Hz, 1H, H13), 6.78 (d, *J* = 6.8 Hz, 1H, H14), 6.76 (d, *J* = 15.9 Hz, 1H, H2), 5.99 (m, 1H, H16), 5.12 (dd, *J* = 21.0, 1.5 Hz, 1H, H17), 5.07 (d, *J* = 10.2 Hz, 1H, H17), 3.81 (s, 6H, H8), 3.74 (s, 3H, H18), 3.38 (d, *J* = 6.8 Hz, 2H, H15). **^13^C NMR** (75 MHz, DMSO-*d*_6_): δ (ppm) = 164.9 (C1), 150.9 (C10), 148.1 (C6), 147.2 (C3), 138.7 (C9 or C12), 138.7 (C9 or C12), 137.7 (C7), 137.5 (C16), 124.3 (C4), 122.8 (C14), 120.3 (C13), 116.1 (C17), 113.8 (C2), 112.9 (C11), 106.6 (C5), 56.1 (C8), 55.7 (C18), 39.4 (C15). **λ_max_** (EtOH, nm) 337. **ε** (L·mol^−1^·cm^−1^): 25991. **Mp**: 140–142 °C. **HRMS** (*m/z*) [M + H]^+^ calcd for C_21_H_23_O_6_: 371.1495; found: 371.1491.

#### 2.1.3. Methylation of (**4**)

2-Ethylhexyl sinapate (**1**) (212 mg, 0.63 mmol, 0.5 M) was dissolved in anhydrous DMF (1.2 mL) and placed under N_2_. After 10 min of stirring, K_2_CO_3_ (248 mg, 1.79 mmol, 3 eq) and methyl iodide (45 μL, 0.71 mmol, 1.2 eq) were added and the reaction was stirred overnight under N_2_ at r.t. The mixture was then diluted in 1M aqueous HCl and partitioned between ethyl acetate and H_2_O. The organic layer was dried over anhydrous MgSO_4_, filtered and concentrated to afford **methylated 2-ethylhexyl sinapate (2)** as a yellow oil (89% yield). **^1^H NMR** (300 MHz, DMSO-*d*_6_): δ (ppm) 7.58 (d, *J* = 16.0 Hz, 1H, H3), 7.08 (s, 2H, H5), 6.66 (d, *J* = 16.0 Hz, 1H, H2), 4.07 (dd, *J* = 5.7, 2.2 Hz, 2H, H10), 3.82 (s, 6H, H8), 3.68 (s, 3H, H9), 1.61 (m, 1H, H11), 1.29 (m, 8H, H12 + H13 + H14 + H16), 0.88 (m, 6H, H15 + H17). **^13^C NMR** (75 MHz, DMSO-*d_6_*): δ (ppm) 166.6 (C1), 153.1 (C6), 144.7 (C3), 139.4 (C7), 129.6 (C4), 117.4 (C2), 106.0 (C5), 66.0 (C10), 60.1 (C9), 56.1 (C8), 38.3 (C11), 29.8 (C12), 28.4 (C13), 23.2 (C16), 22.5 (C14), 14.0 (C15), 10.8 (C17). **λ_max_** (EtOH, nm) 308. **ε** (L·mol^−1^·cm^−1^): 14173. **HRMS** (*m/z*) [M + H]^+^ calcd for C_20_H_31_O_5_: 351.2171; found: 351.2172.

#### 2.1.4. Acetylation of (**4**)

2-Ethyhexyl sinapate (**1**) (202 mg, 0.60 mmol, 1 M) was dissolved in acetic anhydride (300 μL, 2.97 mmol, 5 eq) and pyridine (600 μL, 7.5 mmol) and stirred at r.t. overnight. The mixture was then diluted in 1M aqueous HCl and partitioned between ethyl acetate and H_2_O. The organic layer was dried over anhydrous MgSO_4_, filtered and concentrated to afford **acetylated 2-ethylhexyl sinapate (3)** as a pale-yellow oil (83% yield). **^1^H NMR** (300 MHz, DMSO-*d*_6_): δ (ppm) 7.61 (d, *J* = 16.0 Hz, 1H, H3), 7.16 (s, 2H, H5), 6.76 (d, *J* = 16.0 Hz, 1H, H2), 4.08 (dd, *J* = 5.7, 2.3 Hz, 2H, H11), 3.80 (s, 6H, H8), 2.25 (m, 3H, H10), 1.61 (m, 1H, H12), 1.29 (m, 8H, H13 + H14 + H15 + H17), 0.87 (m, 6H, H16 + H18). **^13^C NMR** (75 MHz, DMSO-*d*_6_): 168.0 (C9), 166.4 (C1), 152.0 (C6), 144.4 (C3), 132.5 (C7), 132.0 (C4), 118.6 (C2), 105.4 (C5), 66.0 (C11), 56.2 (C8), 39.3 (C12), 29.8 (C13), 28.4 (C14), 23.2 (C17), 22.5 (C15), 20.2 (C10), 14.0 (C16), 10.8 (C18). **λ_max_** (EtOH, nm) 299. **ε** (L·mol^−1^·cm^−1^): 18046. **HRMS** (*m/z*) [M + H]^+^ calcd for C_21_H_31_O_6_: 379.2121; found: 379.2120.

#### 2.1.5. Amide Derivatives

Meldrum’s acid (1.44 g, 10 mmol, 0.2 M) and the corresponding amine (10 mmol, 1 eq) were mixed in acetonitrile (50 mL), the resulting mixture was heated at 60 °C for 4 h. After cooling at r.t., acetonitrile was evaporated under vacuum to afford the corresponding intermediary. The crude product was used without purification, mixed with syringaldehyde (12 mmol, 1.2 eq), aniline (1.2 mmol, 0.1 eq) and dissolved in pyridine (6.7 mL). The mixture was heated at 60 °C overnight. After cooling at r.t. the reaction was partitioned between AcOEt and 1M aqueous HCl. The organic layer was dried over anhydrous MgSO_4_, filtered and concentrated prior to purification by flash chromatography using cyclohexane/AcOEt as eluant.

***N*-phenyl sinapoylamide (15)** was synthesized as a yellow powder (25% yield) using aniline. **^1^H NMR** (300 MHz, DMSO-*d*_6_): δ (ppm) 10.10 (s, 1H, NH), 8.91 (s, 1H, OH), 7.69 (d, *J* = 7.6 Hz, 2H, H10), 7.50 (d, *J* = 15.6 Hz, 1H, H3), 7.32 (t, *J* = 7.9 Hz, 2H, H11), 7.04 (t, *J* = 7.4 Hz, 1H, H12), 6.92 (s, 2H, H5), 6.67 (d, *J* = 15.6 Hz, 1H, H2), 3.82 (s, 6H, H8). **^13^C NMR** (75 MHz, DMSO-*d*_6_): δ (ppm) 164.0 (C1), 148.1 (C6), 141.0 (C3), 139.6 (C9), 137.7 (C7), 128.8 (C11), 125.1 (C4), 123.2 (C12), 119.3 (C2), 119.1 (C10), 105.4 (C5), 56.0 (C8). **λ_max_** (EtOH, nm) 336. **ε** (L·mol^−1^·cm^−1^): 25757. **Mp**: 74–77 °C. **HRMS** (*m/z*) [M + H]^+^ calcd for C_17_H_18_NO_4_: 300.1236; found: 300.1235.

***N*-butyl sinapoylamide (16)** was synthesized as a yellow powder (13% yield) using *n*-butylamine. **^1^H NMR** (300 MHz, DMSO*-d*_6_): δ (ppm) 8.78 (s, 1H, OH), 7.92 (t, *J* = 5.6 Hz, 1H, NH), 7.30 (d, *J* = 15.7 Hz, 1H, H3), 6.84 (s, 2H, H5), 6.46 (d, *J* = 15.7 Hz, 1H, H2), 3.79 (s, 6H, H8), 3.15 (q, *J* = 6.7 Hz, 2H, H9), 1.40 (m, 2H, H10), 1.30 (m, 2H, H11), 0.88 (t, *J* = 7.2 Hz, 3H, H12). **^13^C NMR** (75 MHz, DMSO-*d*_6_): δ (ppm) 165.2 (C1), 148.1 (C6), 141.0 (C3), 139.6 (C9), 137.7 (C7), 128.8 (C11), 125.1 (C4), 123.2 (C12), 119.3 (C2), 119.1 (C10), 105.4 (C5), 56.0 (C8). **λ_max_** (EtOH, nm) 320. **ε** (L·mol^−1^·cm^−1^): 17655. **Mp**: 104–106 °C. **HRMS** (*m/z*) [M + H]^+^ calcd for C_15_H_22_NO_4_: 280.1549; found: 280.1550.

#### 2.1.6. Ketone Derivative

Syringaldehyde (1 g, 5.5 mmol) was dissolved in acetone (50 mL) and placed in an ice bath. A solution of NaOH (3.5 g, 87.5 mmol) in H_2_O (35 mL) was added dropwise. After complete addition, the reaction mixture was stirred until it reached room temperature. Excess of NaOH was neutralized with aqueous 1 M HCl and the mixture was extracted with ethyl acetate. The organic layer was dried over anhydrous MgSO4, filtered and evaporated under reduced pressure to afford **4-(4-Hydroxy-3,5-dimethoxyphenyl)-3-buten-2-one (17)** as a yellow powder (61% yield). **^1^H NMR** (300 MHz, DMSO-*d*_6_): δ (ppm) 9.06 (s, 1H, OH), 7.52 (d, *J* = 16.3 Hz, 1H, H3), 7.02 (s, 2H, H5), 6.72 (d, *J* = 16.3 Hz, 1H, H2), 3.80 (s, 6H, H8), 2.29 (s, 3H, H9).**^13^C NMR** (75 MHz, DMSO-*d*_6_): δ (ppm) = 198.0 (C1), 148.1 (C6), 144.5 (C3), 138.4 (C4), 124.7 (C2), 106.3 (C5), 56.1 (C8), 27.2 (C9). **λ_max_** (EtOH, nm) 344. **ε** (L·mol^−1^·cm^−1^): 22593. **Mp**: 136–138 °C. **HRMS** (*m/z*) [M + H]^+^ calcd for C_12_H_15_O_4_: 223.0970; found: 223.0963.

#### 2.1.7. UV Analysis & Loss of Absorbance (LoA)

A Cary 60 UV-Vis (Agilent) was used to record UV/Vis spectra from initial solutions of the desired compounds at 10 μM in ethanol placed in 1 cm quartz cuvette and are reported in wavelength (nm). Those solutions were then irradiated for one hour in a Rayonet^®^ RPR-200 (λ = 300 nm, P = 8.32 W/m^2^, stirring, T = 35 °C) from the Southern New England Ultraviolet Company (Branford, CT, USA) using 14 RPR-3000A lamps. The resulting absorbance was then compared to the initial one and LoA was calculated in percentage at the λ_max_.

#### 2.1.8. DPPH Inhibition

Antiradical activities were determined using the 2,2-diphenyl-1-picrylhydrazyl (DPPH) assay to provide EC_50_ values [33]. To a well containing 10 μL of potential antiradical solution in ethanol (concentrations ranging from 400 to 12.5 μM), 190 μL of DPPH solution (C = 200 μM in ethanol) was added. Reaction was carried out in a Multiskan FC system (Thermo Fisher Scientific, Waltham, MA, USA) and disappearance of the DPPH radicals was monitored at 520 nm every 5 min for 7.5 h in duplicate. The use of different concentrations of potential antiradical gave the EC_50_ value, which is the quantity needed to reduce half the initial population of DPPH radicals.

## 3. Results and Discussion

**Scheme 1:**
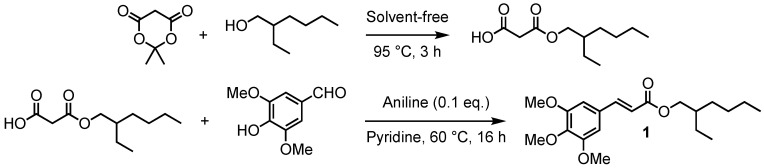
Two-step synthetic pathway leading to octinoxate analogues.

As previously described, the most common approach to access *p*-hydroxycinnamates is through the reaction of the corresponding acyl chloride and an alcohol. However, when the desired ester has a free phenol moiety, it is necessary to protect/deprotect it selectively. This last step remains very tedious taking into account α,β-unsaturated ester moiety sensitivity, and often leads to low yields. To avoid both these protection/deprotection steps and the use of hazardous acyl chlorides, we have developed a two-step synthetic route involving (1) the obtention of a malonic monoester through the opening of Meldrum’s acid [34,35], and (2) its subsequent Knoevenagel–Doebner condensation with unprotected syringaldehyde [36] for which various synthetic methodologies have been described in the literature [37,38,39]. It is worth mentioning that this two-step procedure was carried out without solvent. Indeed, Meldrum’s acid turns liquid at 95 °C, playing the role of both reagent and solvent [40,41]. This method allowed us to readily access mono-2-ethylhexyl malonate that then underwent Knoevenagel–Doebner condensation with syringaldehyde, in the presence of pyridine and aniline, to provide an octinoxate analogue in sinapic series, 2-ethylhexyl sinapate (**1**) (Scheme 1).

**Figure 2:**
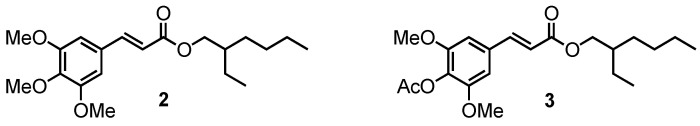
Methylated (**2**) and acetylated (**3**) octinoxate analogues with a sinapic acid core.

In order to determine the impact of the phenol moiety on the UV absorbance, methylation and acetylation of compound **1** were carried out to access compounds **2** and **3**, respectively (Figure 2). Note that compound **2** corresponds to the octinoxate analogue in sinapic acid series.

**Figure 3:**
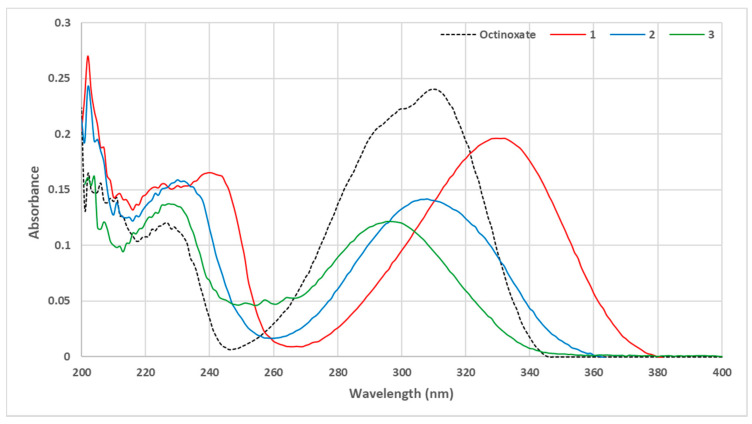
UV Vis spectra of octinoxate analogues bearing sinapic moiety (C = 10 μmol·L^−1^ in ethanol).

To evaluate the UV properties of each compound, a UV-Vis spectrum at 10 µmol·L^−1^ in ethanol was performed for each molecule. Spectra are grouped in Figure 3 and compared with octinoxate as a reference.

**Figure 4:**
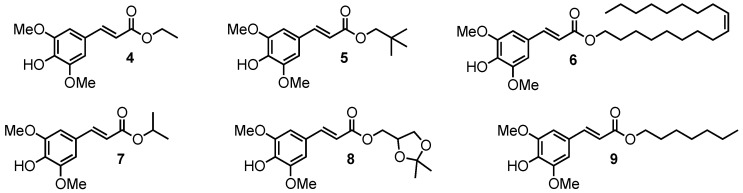
Sinapic acid analogues of octinoxate bearing aliphatic esters.

Unexpectedly, contrary to what could have been assumed from the previous literature [26,27], the use of a sinapic core did not lead to a greater absorbance than that of the coumaric moiety. However, we clearly observed an impact of the phenol protection and that of the protecting group (Me vs. Ac) on the absorbance. Indeed, with the free phenol (compound 1), a hypsochromic shift was observed, giving access to both UVB (280–315 nm) and UVA (315–400 nm) wavelength coverage. The use of an acetate as phenol protecting group (compound **3**) led to a wavelength coverage area comparable to that of octinoxate, just like methylated compound **2**. Nevertheless, there was a slight hypsochromic shift using methylated analogue. In terms of absorbance, the protection of phenol resulted in an important decrease in absorbance. Therefore, if one seeks UV filters with a high absorbance at both UVA and UVB wavelengths, it is preferable to use filters with a free phenol. In an attempt to challenge octinoxate in terms of absorbance intensity, our next strategy consisted of modulating the second key structural feature, i.e., the ester moiety. For this, we used the same synthetic route described above with various aliphatic alcohols (compounds **4**–**9**, Figure 4).

**Figure 5:**
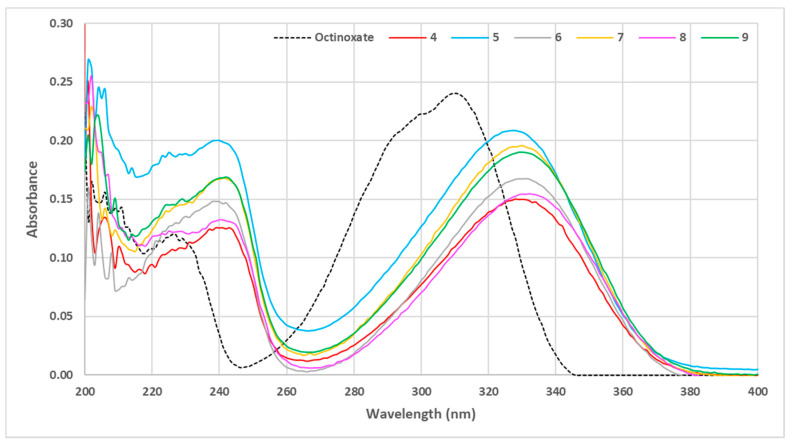
UV visible spectra of aliphatic sinapic acid esters (C = 10 μmol·L^−1^ in ethanol).

The absorbance of those six compounds was evaluated at 10 µmol·L^−1^ in ethanol and the UV-Vis spectra were compared to that of octinoxate as reference (Figure 5).

**Figure 6:**
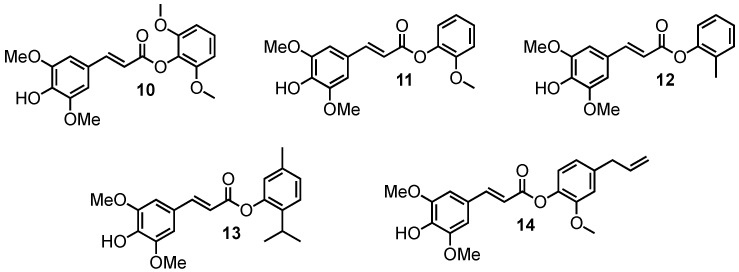
Sinapate analogues of octinoxate bearing phenolic esters.

The use of sparsely sterically hindered primary alcohols led to the esters showing the lowest absorbance spectra (compounds **4** and **8**). Fatty alcohols provided a slight increase in the absorbance capacity; however, when the fatty chain was extremely long (compound **6**), a loss of intensity compared to moderate chain length (compound **9**) was observed. The use of secondary (compound **7**) or tertiary alcohol (compound **5**) resulted in a gain in absorbance (Figure 5). In summary, the presence of a very bulky ester group resulted in an increase in absorbance. It is noteworthy that such an effect is in accordance with previous studies that highlighted that steric hindrance of the ester moiety was crucial for good UV properties on sinapate, as it promotes the *cis*/*trans* isomerization rate of such molecules upon UV exposure [29]. Based on these results, it was decided to continue our study by exploring bulkier phenolic esters **10**–**14** that were readily prepared using the same two-step synthetic pathway, (Figure 6).

**Figure 7:**
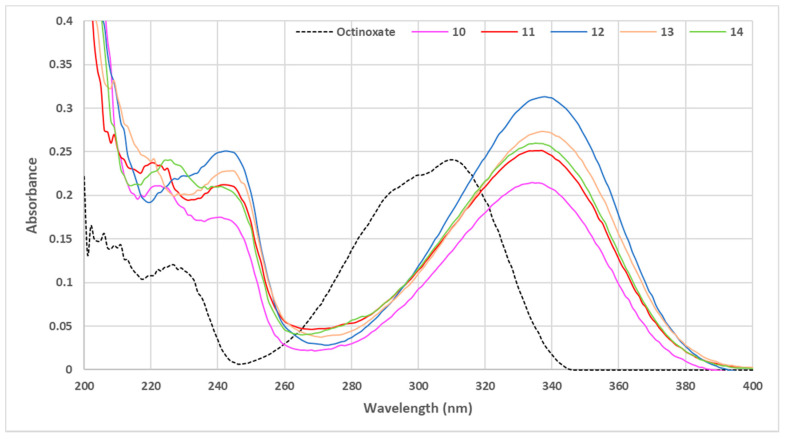
UV visible spectra of phenolic sinapic acid esters (C = 10 μmol·L^−1^ in ethanol).

The UV-Vis absorbance of the five new compounds was determined at 10 µmol·L^−1^ in ethanol. We then compared the spectra obtained with that of octinoxate as a reference (Figure 7).

Overall, the use of phenolic ester moiety (PEM) afforded a boost in UV absorbance, even allowing, in some cases, a greater value than octinoxate (Figure 7). Concerning the three different positions of the substitution on the ester aromatic rings (i.e., *ortho*, *meta* and *para*), only the *ortho* position seemed to induce a significant modification to the absorbance intensity, as the introduction of substituents in the *meta* (compound **13**) or *para* (compound **14**) position did not modify further the absorbance. While the introduction of various steric hindrance to the *ortho* position of the PEM influenced the level of absorbance for the corresponding compounds, conversely to what we observed with aliphatic sinapate, higher steric hindrance did not result in better absorbance on the *ortho* position. With a simple methyl group as substituent (compound **12**), the resulting absorbance was the best of all synthesized esters. The presence of a methoxy group (compound **11**) in place of methyl on the PEM gave similar properties to the *meta*/*para* substitutions and finally, substitution at both *ortho* positions (compound **10**) led to the lowest result. If the use of phenolic ester moiety proved that steric hindrance leads to better UV properties, it also highlighted that a too-high hindrance at the ester position (i.e., presence of two bulky groups in *ortho* positions) negatively impacted the absorbance properties. In conclusion, the steric congestion of the ester group has to be carefully tuned to afford optimal results.

**Figure 8:**
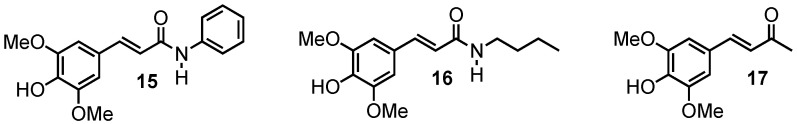
Sinapic acid analogues of octinoxate with amides or ketone groups.

To go even further, we sought to identify whether the nature of the carbonyl function could also impact the UV absorption. For this, two amide derivatives, as well as a ketone, have been synthesized with a sinapic acid core (compounds **15**–**17**, Figure 8).

**Figure 9:**
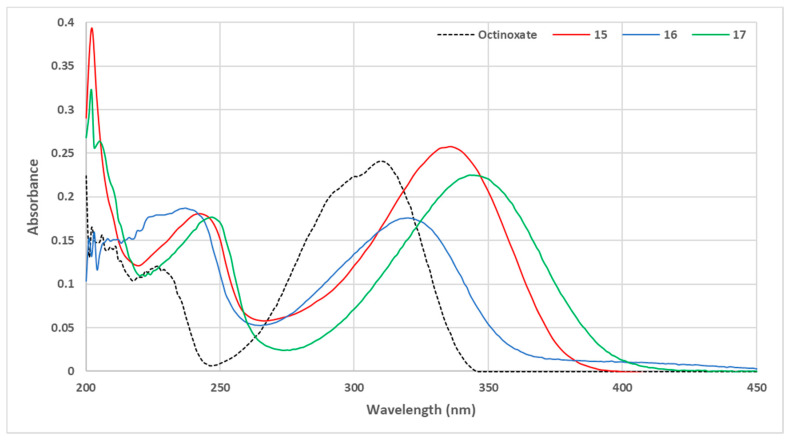
UV visible spectra of sinapic acid amide and ketone (C = 10 μmol·L^−1^ in ethanol).

UV-vis spectra at 10 µmol·L^−1^ in ethanol were obtained for compounds **15**–**17** and are reported in Figure 9.

The aniline-based *p*-hydroxycinnamide **15** showed an absorbance similar to those obtained with phenolic ester (compounds **11**–**14**), thus confirming the importance of steric hindrance on the carbonyl, as previously observed. Aliphatic amide **16** exhibited an absorbance similar to those of aliphatic esters (compounds **4**,**6**,**8**). Nevertheless, while the nature of the carbonyl (i.e., ester vs. amide) had no significant impact on the UV properties, the presence of a methyl ketone resulted in a slight bathochromic shift with the level of absorbance similar to the bulkiest aliphatic ester **5**.

The above SARs studies highlight the importance of having a free phenol on the molecule to cover both UVA and UVB wavelengths. Moreover, the steric hindrance at the ester position is essential to obtain a high protection but must be carefully chosen to avoid a negative impact on the absorption of such compounds. The nature of the carbonyl function (ester or amide) is of little importance, although the second one is known to be more stable to hydrolysis. To conclude this study on the UV filtering properties, the photostability of the various compounds was assessed.

### 3.1. Loss of Absorbance (LoA)

**Figure 10:**
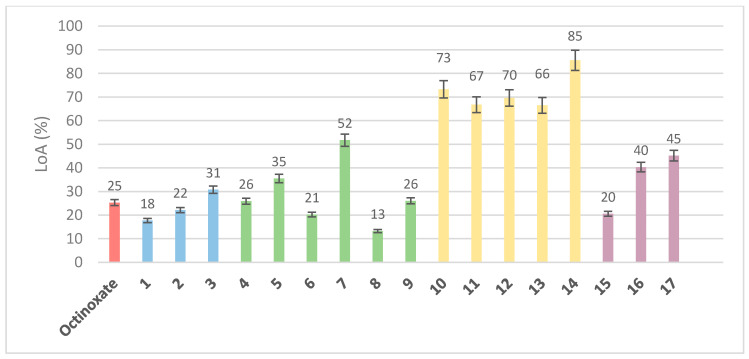
Loss of Absorbance in percentages after UV irradiation (λ = 300 nm, P = 8.32 W/m^2^, stirring, T = 35 °C) for 60 min (C = 10 μmol·L^−1^ in ethanol): reference (red), octinoxate sinapic based analogues (blue, **1**–**3**), aliphatic sinapic acid esters series (green, **4**–**9**), phenolic sinapic acid esters series (yellow, **10**–**14**) and amide and ketone sinapic acid series (purple, **15**–**17**). Each value is presented with the mean ± standard error.

In addition to the coverage area and the absorbance intensity, the photostability upon UV irradiation (i.e., loss of absorbance) is important when one seeks alternatives to current UV filters. For this, solutions in ethanol (C = 1 mmol·L^−1^) were irradiated for 60 min under UV light (λ = 300 nm, P = 8.32 W/m^2^, stirring, T = 35 °C). The absorbance of the resulting solutions was compared to that of the non-irradiated solutions (t = 0) to determine the percentage of absorbance loss (Figure 10).

When looking at compounds **1**–**3** (sinapic acid esters octinoxate analogues), it appears that the sinapic core provides identical results than octinoxate in terms of loss of absorbance. Indeed, after 1 h of irradiation, octinoxate exhibited 25% loss of absorbance when the sinapic analogue (**1**) loses 18% of absorbance. The acetylated analogue (**3**) and the methylated analogue (**2**) seemed somewhat worse, since they displayed 31% and 22% loss of absorbance, respectively. These results further confirm the need to favor analogues with free rather than protected phenols. With regards to the aliphatic sinapate series (compounds **4**–**9**), one can observe that primary alcohols exhibited interesting photostabilities, close to or better than that of octinoxate, with a loss of absorbance of less than 15% for compound **8**. Secondary (**7**) or tertiary (**5**) alcohols proved to be less photostable, with a loss of absorbance higher than 30% (52% and 35%, respectively). These results undoubtedly prove that the nature and steric hindrance of the ester moiety play a significant role on the UV filter photostability. As for the phenolic ester series (compounds **10**–**14**), it clearly appeared that these analogues were very unstable toward UV irradiation, as all compounds lost more than 60% of their initial absorbance. As phenolic esters (R-CO_2_-Ar) are known to be easily hydrolyzed [42], one can assume that they are also more prone to photodegradation than aliphatic esters under UV irradiation. Despite their better absorbance properties, phenolic esters proved to be poor candidates as UV filters with regards to their high loss of absorbance, as did the ketone analogue (compound **17**), with an intermediate behavior with a loss of absorbance of 45%, which was almost twice as high as octinoxate. Finally, aromatic amide **15** seemed to be an interesting compromise. Indeed, while the phenolic derivatives collapsed, compound **15** showed an interesting photostability (LoA of 20%), even better than octinoxate (25%). However, compared to esters **4**–**14** where aliphatic moieties led to lower LoA, the trend was reversed for amides. Indeed, the compound with butylamine **16** was less stable than its analogues in aromatic series (compound **15**). It is noteworthy that an in-depth kinetic study of the photostability of all compounds upon UVA/UVB irradiation, as well as the structural elucidation of the potential photoproducts, will be performed and reported in due course.

In order to stabilize sunscreen formulations, but also to protect the skin, antioxidants are usually added to limit Reactive Oxygen Species (ROS) formation at the dermis level and thus maintain cellular homeostasis. As multifunctional compounds are increasingly sought after by manufacturers in the cosmetics industry, the last step of this study was to determine whether one or various compounds could be both a potent photostable UV filter and a strong antioxidant to limit the number of ingredients in sunscreen formulations.

### 3.2. Antioxidant Activities

To assess the antiradical potential of the synthesized molecules, tests were performed using DPPH (2,2-diphenyl-1-picrylhydrayl) as a free radical and by monitoring its disappearance. This allowed the determination of EC_50_ values (half maximal effective concentration), defined as the quantity (in mmol) of synthesized compound needed to reduce 50% of free radicals. The lower the EC_50_, the better the antiradical capacity. Once determined, the EC_50_ values were used to assess the kinetics of the radical scavenging for each compound. To do so, T_EC50_, the time needed to reach the steady state of radical inhibition with a quantity of antiradical corresponding to its EC_50_, was determined. This parameter allowed the sorting of antiradical properties into three kinetic behaviors: rapid (T_EC50_ < 300 s), intermediate (300 < T_EC50_ < 1800 s) and slow (T_EC50_ > 1800 s) as described previously by Sánchez-Moreno et al. [43]. Results are shown in Table 1.

As expected, molecules bearing a protected phenol group (Octinoxate, **2** and **3**) did not exhibit any antiradical properties. All tested compounds with free phenol and ester moiety (compounds **1**, **4**–**14**) resulted in EC_50_ within a 13.7–21.1 nmol range. This showed that antiradical activity did not depend on the nature of the ester group (i.e., aliphatic or phenol esters) in a significative manner. However, the introduction of amide (compounds **15** and **16**) in place of the ester provided better antioxidant properties. The presence of an anilide (R-CO-NH-Ph) moiety resulted in significantly lower EC_50_ than the corresponding esters (compounds **10**–**14**), while the aliphatic amide **16** provided a slightly lower value than its ester counterparts (compounds **1**, **4**–**9**). The presence of a hydrogen on the amide could explain those results; indeed, it could react with free radicals in addition to the phenol [44,45]. Finally, the use of a ketone (compound **17**) also provided an interesting EC_50_ close to the one obtained with compound **15**. In order to evaluate the potential of the synthesized compounds as antioxidants, their EC_50_ were benchmarked against butylated hydroxyanisole (BHA) and butylated hydroxytoluene (BHT), two molecules commonly used as antioxidants at the industrial scale. Although none of the compounds were better than BHA, compounds **15** and **17** were more active than BHT. Regarding their kinetics, all synthesized compounds reached the steady state of radical inhibition in a maximum of 600 s, exhibiting rapid to intermediate behaviors similar to the BHA reference, and significantly better than BHT, which is a slow antiradical.

In addition to the UV and antiradical properties, the toxicity of such compounds should also be evaluated. It has been observed that the presence of methoxy substituents (-OMe) on the aromatic ring significantly decreased the interaction of molecules with endocrine receptor [46], therefore, the use of a sinapic acid core that possesses two -OMe substituents should result in a lower risk of endocrine disruption. Moreover, previous studies in our laboratory proved the innocuousness of several sinapic acid derivatives towards endocrine receptors [47,48]. However, while the sinapic core seems favorable with regards to human health, the impact of the ester moiety on the latter, as well as the possible environmental toxicity of these compounds, should not be overlooked. Such properties will need an in-depth study beyond the preliminary screening of activities reported herein.

## 4. Conclusions

Herein, the synthesis, UV properties, photostability (loss of absorbance upon UV irradiation) and antiradical properties of octinoxate analogues with a sinapic acid core were described. Sinapic acid derivatives with free phenol were synthesized in two steps without the need of protection/deprotection steps, and exhibited absorbance covering both the UVA and UVB regions. The structure–activity relationships study provided insight into the UV properties of synthesized molecules. Absorbance levels and photostability can be fine-tuned by using more or less bulky substituents or by switching to other functions like amides or ketones. However, an extreme degree of steric hindrance proved to have a negative impact. Finally, all compounds with a free phenol provided encouraging EC_50_ values, with some of them being more active than the industrially used BHT. Sinapate derivatives proved to be multifunctional compounds that could be applied as UV filters and antiradical at the same time in sunscreen formulation.

## Figures and Tables

**Table 1:** EC_50_, T_EC50_ and kinetic behavior of compounds **1**–**17** for DPPH inhibition in ethanol.

Compound	EC_50_ (nmol) ^a^	T_EC50_ (s) ^a^	Kinetic
BHA	6.6 ± 0.2	>4800	Slow
BHT	11.8 ± 1.3	360 ± 13	Intermediate
Octinoxate	- ^b^	- ^b^	- ^b^
**1**	22.2 ± 1.1	180 ± 10	Rapid
**2**	- ^b^	- ^b^	- ^b^
**3**	- ^b^	- ^b^	- ^b^
**4**	18.0 ± 0.9	360 ± 18	Intermediate
**5**	17.0 ± 0.7	360 ± 15	Intermediate
**6**	19.9 ± 1.0	180 ± 9	Rapid
**7**	14.4 ± 0.4	180 ± 9	Rapid
**8**	19.8 ± 1.0	180 ± 11	Rapid
**9**	20.9 ± 1.1	420 ± 32	Intermediate
**10**	21.1 ± 1.1	180 ± 8	Rapid
**11**	17.1 ± 0.8	360 ± 18	Intermediate
**12**	15.3 ± 0.5	240 ± 12	Rapid
**13**	13.7 ± 0.6	480 ± 24	Intermediate
**14**	19.4 ± 0.9	<60	Rapid
**15**	8.9 ± 0.3	600 ± 40	Intermediate
**16**	15.9 ± 0.7	240 ± 12	Rapid
**17**	9.4 ± 0.4	180 ± 10	Rapid

^a^ Each value is the mean ± standard error, ^b^ no activity observed.

## Supplementary Materials

The following are available online at https://www.mdpi.com/2076-3921/9/9/782/s1; ^1^H & ^13^C NMR spectra and details for all compounds (malonate monoesters and sinapate esters).
