# Supplementary material for: Sinapic Acid Esters: Octinoxate Substitutes Combining Suitable UV Protection and Antioxidant Activity
Source: Antioxidants (Basel). 2020 Aug 24;9(9):782. doi: 10.3390/antiox9090782 (PMC7554726; doi:10.3390/antiox9090782)
Supplement: Supplementary file 1 [file antioxidants-09-00782-s001.pdf]

Supplementary Information

Sinapic acid esters: Octinoxate substitutes combining suitable UV protection and antioxidant activity

Cédric Peyrot §[1], Matthieu M. Mention §[1], Fanny Brunissen [1] and Florent Allais[1]*

[a] URD Agro-Biotechnologies Industrielles (ABI), CEBB, AgroParisTech, 51110 Pomacle, France
§ These authors contributed equally to this work
* Corresponding author: florent.allais@agroparistech.fr

Table of Contents

1. General Information 2
2. Malonate monoesters 2
3. NMR Spectra 4
  3.1. Malonate monoesters 4
  3.2. Sinapate esters 15

1. General Information

Syringaldehyde, aniline, pyridine, heptan-1-ol, oleic alcohol, 3,5-dimethylphenol, *o*-cresol, solketal, eugenol, Meldrum's acid and DPPH were purchased from Sigma Aldrich. Thymol, 4-hydroxybenzaldehyde, 2-ethylhexan-1-ol, gaïacol and n-butylamine were purchased from TCI. Concentrated HCl and solvents were purchased from Fisher Scientific and used as received. All chemicals were used directly without purification.

Chromatographic purifications of products were accomplished using a flash-prep LC system puriFlash® 4100 from Interchim with prepacked silica column (30 µm, Interchim PF-Si30-HP), dual wavelength collection (λ = 254/320 nm) in cyclohexane/ethyl acetate eluant. ^{1}H NMR spectra were recorded on a Brucker Fourier 300 (300 MHz) and were calibrated with residual DMSO-*d*$_6$ or $CDCl_3$ protons signals at δ 2.50 or 7.26 ppm respectively. Data are reported as follows: chemical shift (δ ppm), multiplicity (s = singlet, d = doublet, t = triplet, q = quartet, sept = septet, dd = doublet of doublet, td = triplet of doublet, m = multiplet), coupling constant (Hz), integration and assignment. ^{13}C NMR spectra were recorded on a Brucker Fourier 300 (75 MHz) and were calibrated with DMSO-*d*$_6$ or $CDCl_3$ signal at δ 39.52 or 77.16 ppm respectively. Data are reported as follows: chemical shift (δ ppm) and attribution. All NMR assignments were made using COSY, HMBC and HSQC spectra. IR spectra were recorded on an Agilent Cary 630 FTIR Spectrometer and are reported in frequency of absorption (cm^{-1}). Melting points were recorded on a Mettler Toledo MP50 Melting Point System with ME-18552 sample tubes. HRMS were performed on an Agilent 1290 system, equipped with a PDA UV detector, and a 6545 Q-TOF mass spectrometer (Wilmington, DE, USA) with a JetStream ESI probe operating at atmospheric pressure as the source.

2. Malonate monoesters

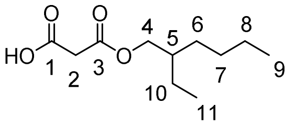

GP1 was followed with 2-ethylhexanol to obtain **mono-2-ethylhexylmalonate** as a yellow oil (83% yield). Characterization data were identical with those already described(Ref)

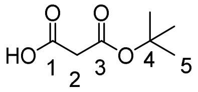

GP2 was followed with *tert*-butanol to obtain **mono-*tert*-butyl malonate** as a pale-yellow oil (97% yield). Characterization data were identical with those already described(Ref)

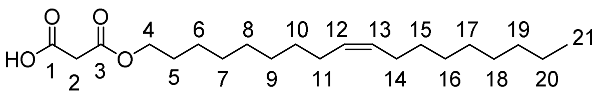

GP1 was followed with oleic alcohol to obtain **mono-oleyl malonate** as a yellow oil (77% yield). **^{1}H NMR** (300 MHz, DMSO-*d*$_6$): δ (ppm) 12.75 (s, 1H, OH), 5.31 (t, *J* = 4.8 Hz, 2H, H12 + H13), 4.03 (t, *J* = 6.6 Hz, 2H, H4), 3.34 (s, 2H, H2), 1.97 (m, 4H, H11 + H14), 1.55 (m, 2H, H5), 1.24 (m, 22H, H6-10 + H15-20), 0.83 (m, 3H, H21). **^{13}C NMR** (75 MHz, DMSO-*d*$_6$): δ (ppm) 168.1 (C1), 167.0 (C3), 129.6 (C12 & C13), 64.6 (C4), 41.6 (C2), 31.4 (C6-10 or C15-20), 28.6-29.0 (C6-10 or C15-20), 28.1 (C5), 26.7 (C6-10 or C15-20), 26.6 (C11 + C14), 25.3 (C6-10 or C15-20), 22.2 (C6-10 or C15-20), 14.0 (C21). **HRMS** (m/z) $[M+H]^+$ calcd for $C_{21}H_{39}O_4$: 355.2848; found: 355.2850.

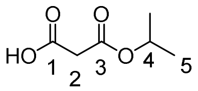

GP2 was followed with iso-propanol to obtain mono-iso-propyl malonate as a pale-yellow oil (76% yield). Characterization data were identical with those already described.(ref)

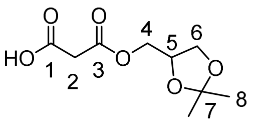

GP1 was followed with solketal to obtain **monosolketal malonate** as a colorless oil (39% yield). ^{1}**H NMR** (300 MHz, $CDCl_3$): δ (ppm) 4.35 (m, 1H, H5), 4.26 (dd, *J* = 11.4, 4.5 Hz, 1H, H4), 4.18 (dd, *J* = 11.4, 6.0 Hz, 1H, H4), 4.09 (dd, *J* = 8.5, 6.4 Hz, 1H, H6), 3.77 (dd, *J* = 8.6, 5.8 Hz, 1H, H6), 3.49 (s, 2H, H2), 1.43 (s, 3H, H8 or H9), 1.36 (s, 3H, H8 or H9). ^{13}**C NMR** (75 MHz, $CDCl_3$): δ (ppm) 171.1 (C1), 167.2 (C3), 110.7 (C7), 73.8 (C5), 66.6 (C4), 66.4 (C6), 41.1 (C2), 27.2 (C8 or C9), 25.8 (C8 or C9). **HRMS** (m/z) $[M+H]^+$ calcd for $C_9H_{15}O_6$: 219.0869; found: 219.0870.

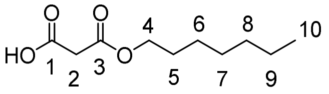

GP1 was followed with heptan-1-ol to obtain **monoheptyl malonate** as a colorless oil (77% yield). Characterization data were identical with those already described(Ref)

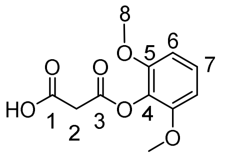

GP1 was followed with 2,6-dimethoxyphenol to obtain **monosyringol malonate** as a white solid (72% yield). ^{1}**H NMR** (300 MHz, $CDCl_3$): δ (ppm) 7.16 (t, *J* = 8.5 Hz, 1H, H7), 6.61 (d, *J* = 2H, H6), 3.82 (s, 6H, H8), 3.75 (s, 2H, H2). ^{13}**C NMR** (75 MHz, $CDCl_3$): δ (ppm) 169.9 (C1), 165.4 (C3), 152.2 (C4), 128.3 (C5), 127.0 (C7), 105.0 (C6), 56.3 (C8), 40.0 (C2). **Mp**: 62-65 °C. **HRMS** (m/z) $[M+H]^+$ calcd for $C_{11}H_{13}O_6$: 241.0712; found: 241.0720.

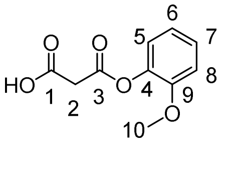

GP1 was followed with gaïacol to obtain **monogaïacol malonate** as a white solid (60% yield). Characterization data were identical with those already described(Ref)

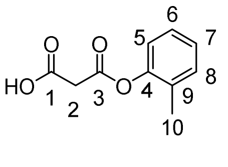

GP1 was followed with *o*-cresol to obtain **monocresol malonate** as a white solid (75% yield). ^{1}**H NMR** (300 MHz, DMSO-d_6): δ (ppm) 13.01 (s, 1H, OH), 7.24 (m, 3H, H6 + H7 + H8), 7.05 (dd, *J* = 7.7, 1.6 Hz, 1H, H5), 3.70 (s, 2H, H2), 2.14 (s, 3H, H10). ^{13}**C NMR** (75 MHz, DMSO-d_6): δ (ppm) 168.0 (C1), 165.5 (C3), 148.9 (C4), 131.1 (C6), 130.0 (C9), 127.1 (C8), 126.2 (C7), 122.0 (C5), 41.5 (C2), 15.6 (C10).). **Mp**: 52-55 °C. **HRMS** (m/z) $[M+H]^+$ calcd for $C_{10}H_{11}O_4$: 195.0657; found: 195.0650.

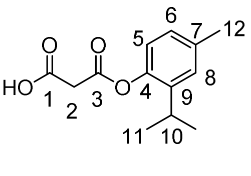

GP1 was followed with thymol to obtain **monothymol malonate** as a pale-yellow oil (79% yield). ^{1}**H NMR** (300 MHz, DMSO-d_6): δ (ppm) 13.00 (s, 1H, OH), 7.24 (d, *J* = 7.9 Hz, 1H, H5), 7.06 (ddd, *J* = 7.9, 1.6, 0.6 Hz, 1H, H6), 6.83 (d, *J* = 1.0 Hz, 1H,

H8), 3.68 (s, 1H, H2), 3.02 (sept, J = 6.8 Hz, 1H, H10), 2.26 (s, 3H, H12), 1.11 (d, J = 6.9 Hz, 6H, H11). **^{13}C NMR** (75 MHz, DMSO-d_6): δ (ppm) 167.9 (C1), 165.9 (C3), 147.4 (C4), 136.9 (C9), 136.2 (C7), 127.2 (C6), 126.5 (C5), 122.5 (C8), 41.6 (C2), 26.1 (C10), 23.0 (C11), 20.3 (C12). **HRMS** (m/z) [M+H]$^+$ calcd for $C_{13}H_{17}O_4$: 237.1127; found: 237.1123.

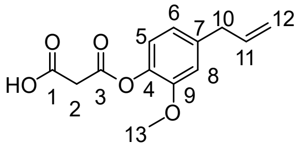

GP1 was followed with eugenol to obtain **mono-eugenol malonate** as a yellow oil (36% yield). **^{1}H NMR** (300 MHz, DMSO-d_6): δ (ppm) 12.94 (s, 1H, H14), 6.98 (d, J = 8.1 Hz, 1H, H5), 6.96 (d, J = 2.9 Hz, 1H, H8), 6.77 (dd, J = 8.0, 2.0 Hz, 1H, H6), 5.97 (m, 1H, H11), 5.10 (m, 2H, H12), 3.75 (s, 3H, H13), 3.63 (s, 2H, H2), 3.37 (d, J = 6.8 Hz, 1H, H10). **^{13}C NMR** (75 MHz, DMSO-d_6): δ (ppm) 167.7 (C1), 165.3 (C3), 150.6 (C9), 139.2 (C4), 137.5 (C11), 137.2 (C7), 122.4 (C5), 120.4 (C6), 116.1 (C12), 113.1 (C8), 55.8 (C13), 41.2 (C2), 39.4 (C10). **HRMS** (m/z) [M+H]$^+$ calcd for $C_{13}H_{15}O_5$: 251.0919; found: 251.0917.

3. NMR Spectra

3.1. Malonate monoesters

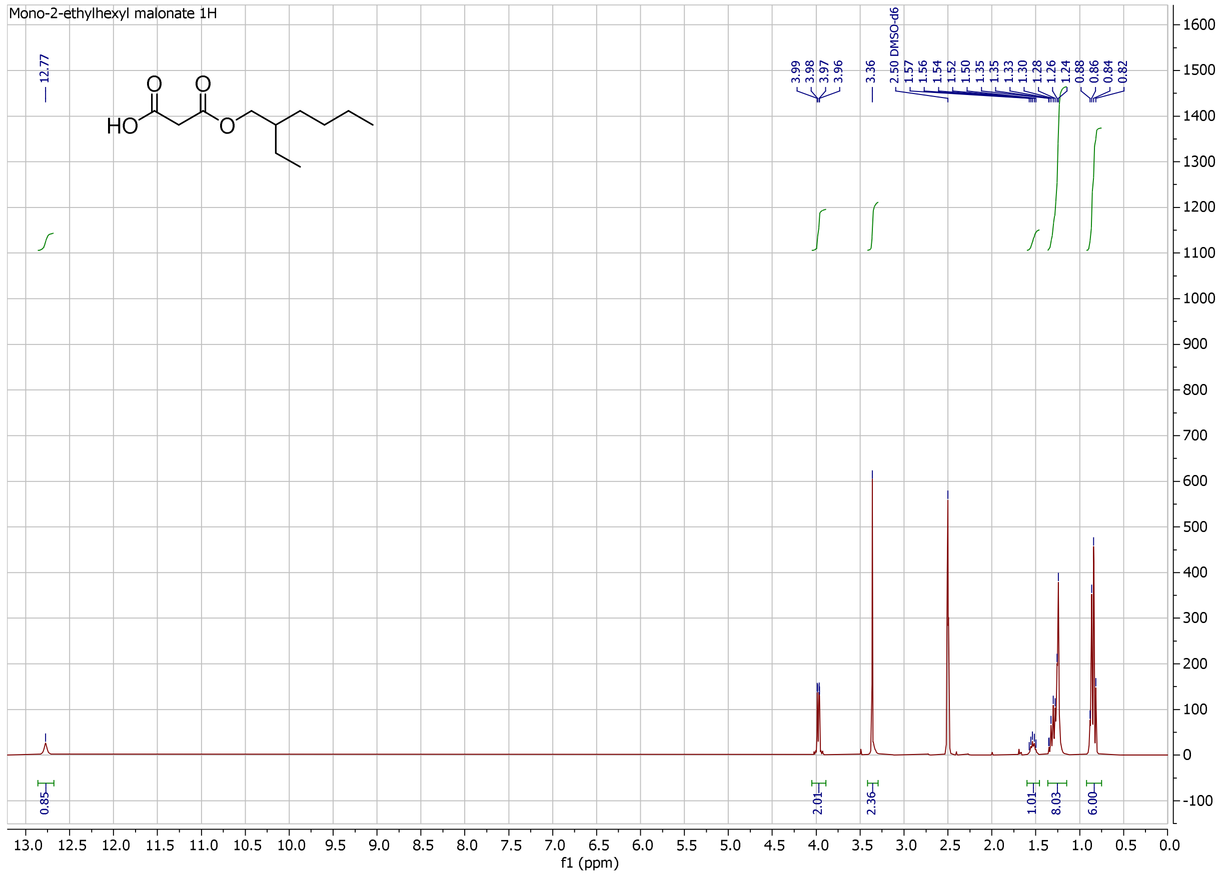

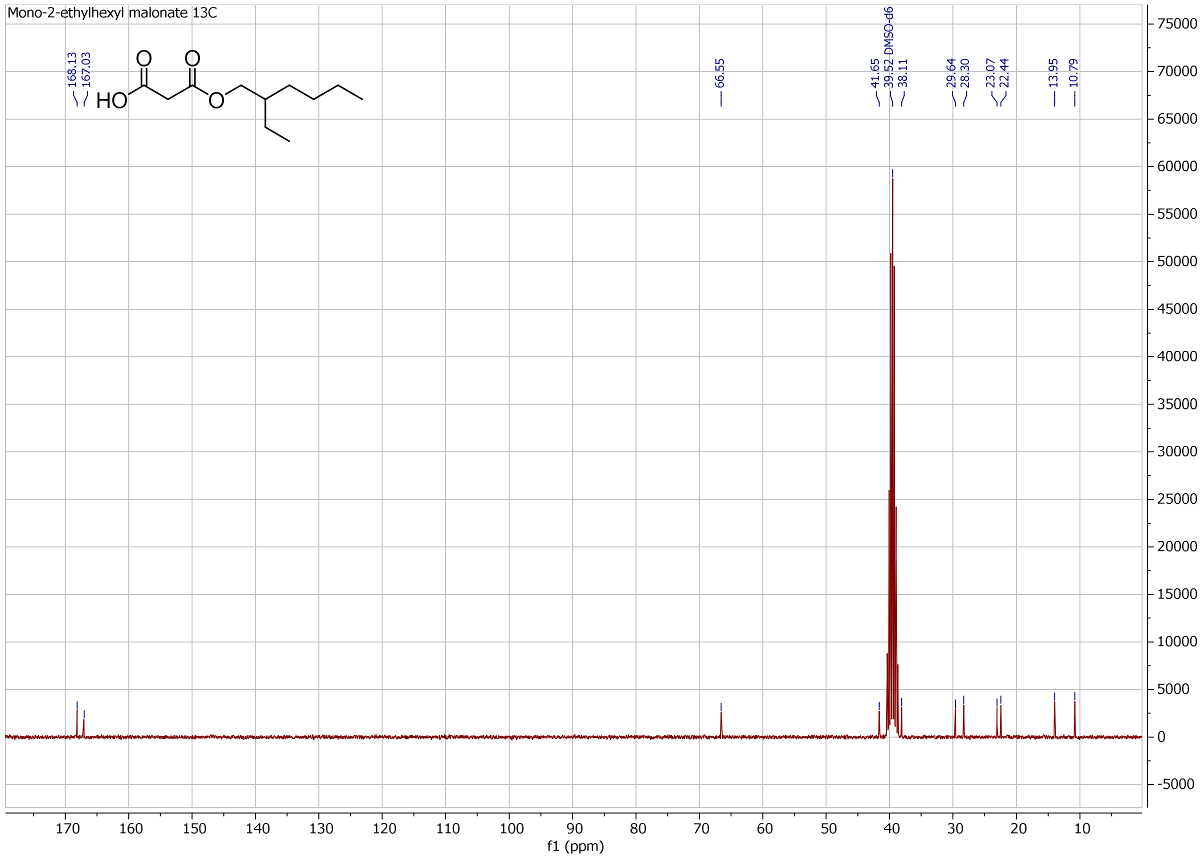

Mono-2-ethylhexyl malonate 13C
HO
O
O
O
168.13
167.03
66.55
41.65
39.52 DMSO-d6
38.11
29.64
28.30
23.07
22.44
13.95
10.79
f1 (ppm)

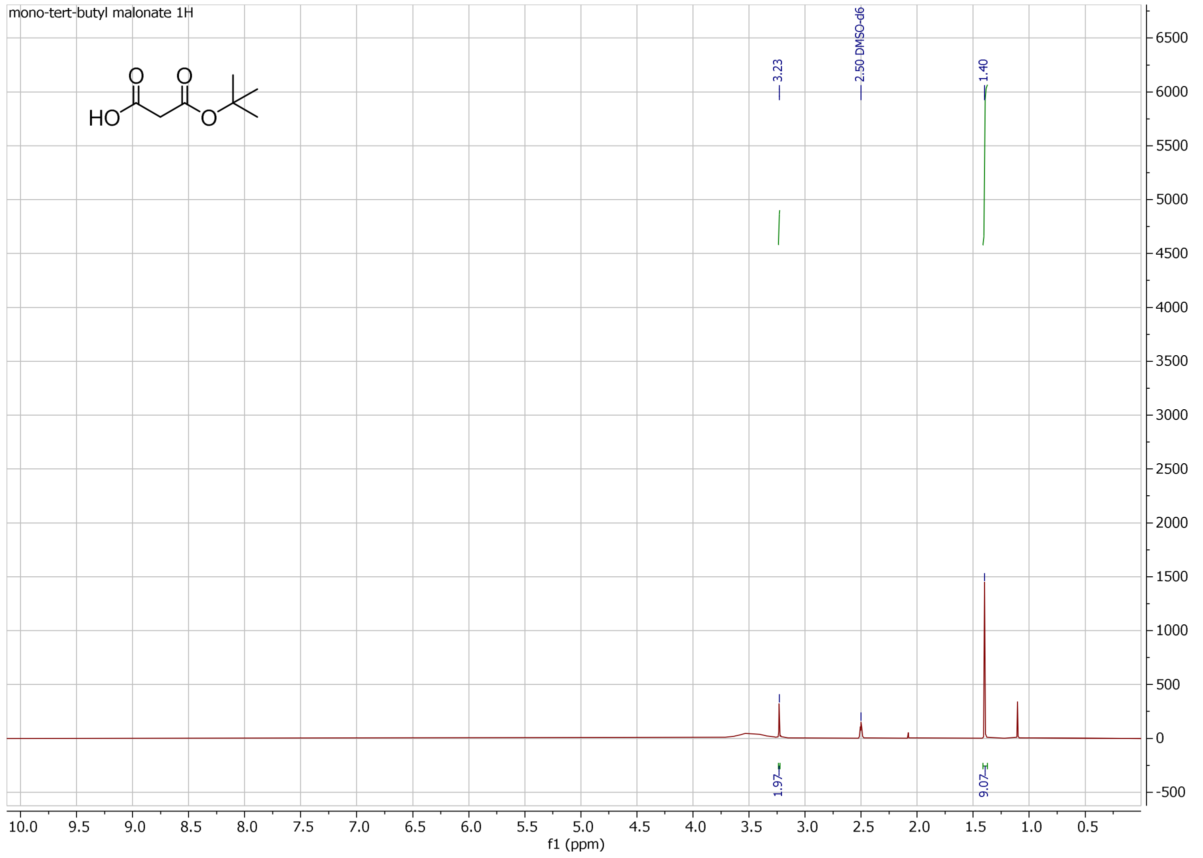

mono-tert-butyl malonate 1H
HO
O
O
O
3.23
2.50 DMSO-d6
1.40
1.97
9.07
f1 (ppm)

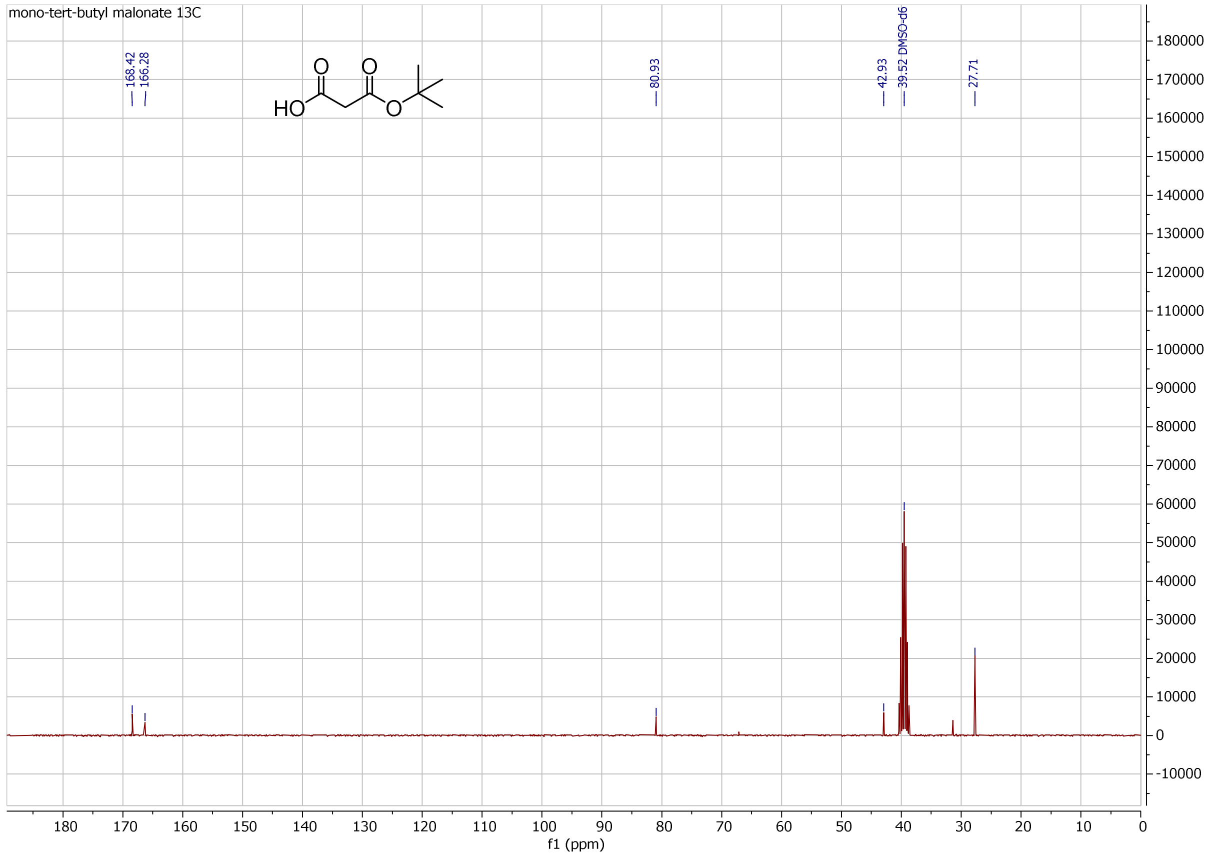
mono-tert-butyl malonate 13C
168.42
166.28
80.93
42.93
39.52 DMSO-d6
27.71
HO
O
O
O
f1 (ppm)

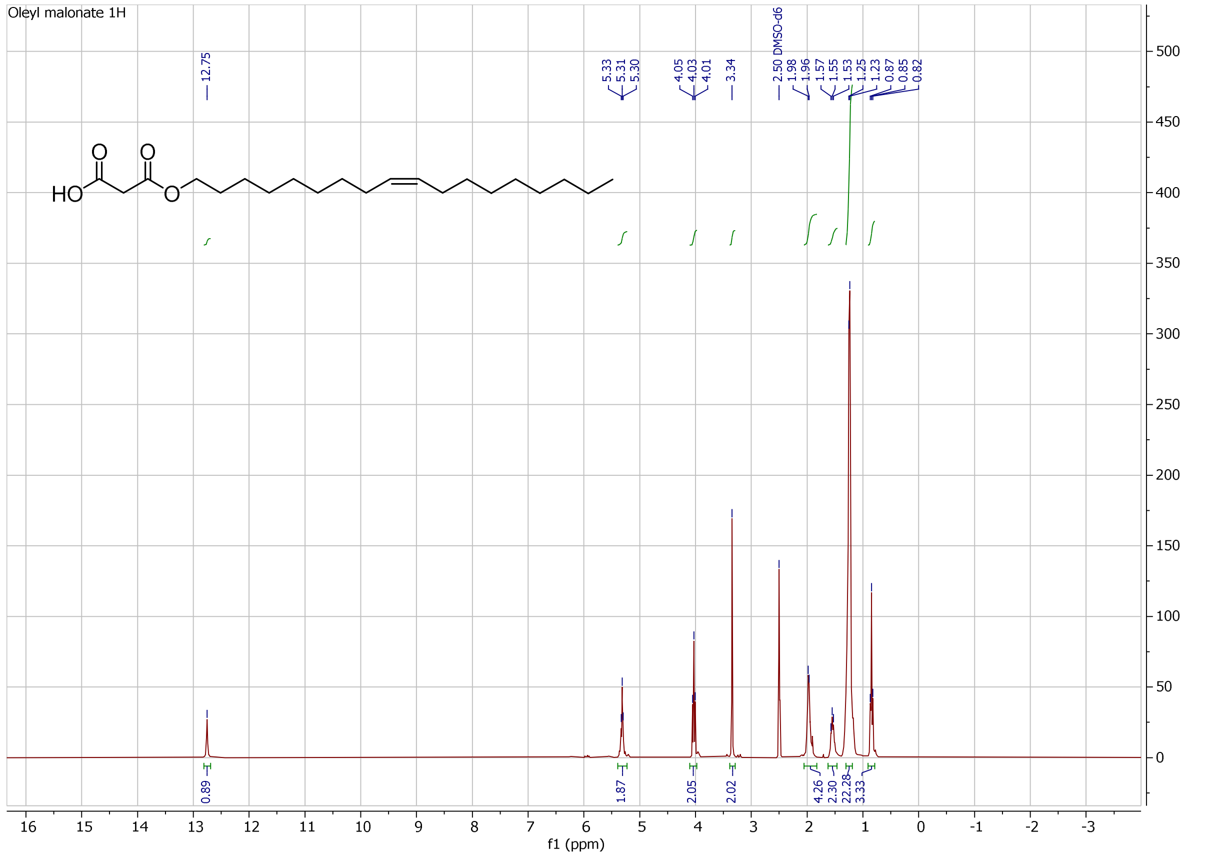
Oleyl malonate 1H
12.75
5.33
5.31
5.30
4.05
4.03
4.01
3.34
2.50 DMSO-d6
1.98
1.96
1.57
1.55
1.53
1.25
1.23
0.87
0.85
0.82
HO
O
O
O
0.89
1.87
2.05
2.02
4.26
2.30
22.28
3.33
f1 (ppm)

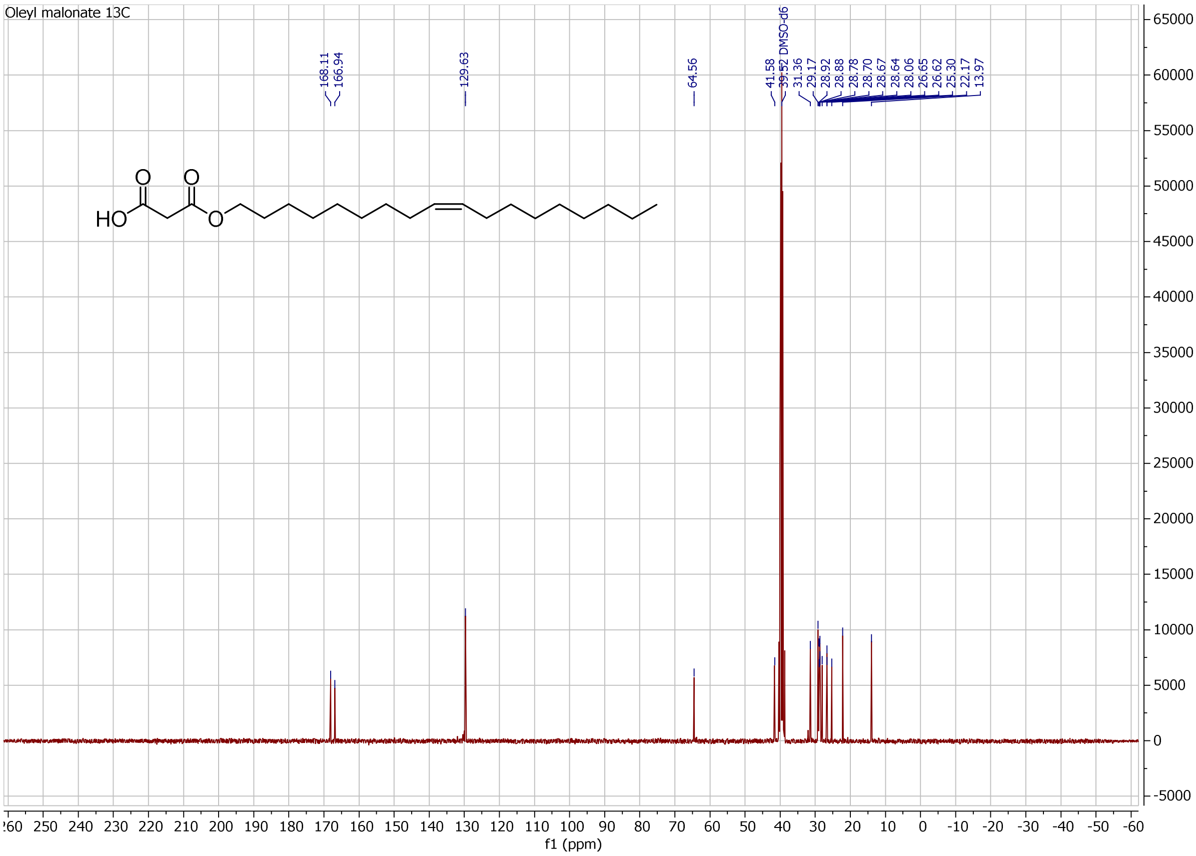
Oleyl malonate 13C
168.11
166.94
129.63
64.56
41.58
39.52 DMSO-d6
31.36
29.17
28.92
28.88
28.78
28.70
28.67
28.64
28.06
26.65
26.62
25.30
22.17
13.97
HO
O
O
O
f1 (ppm)

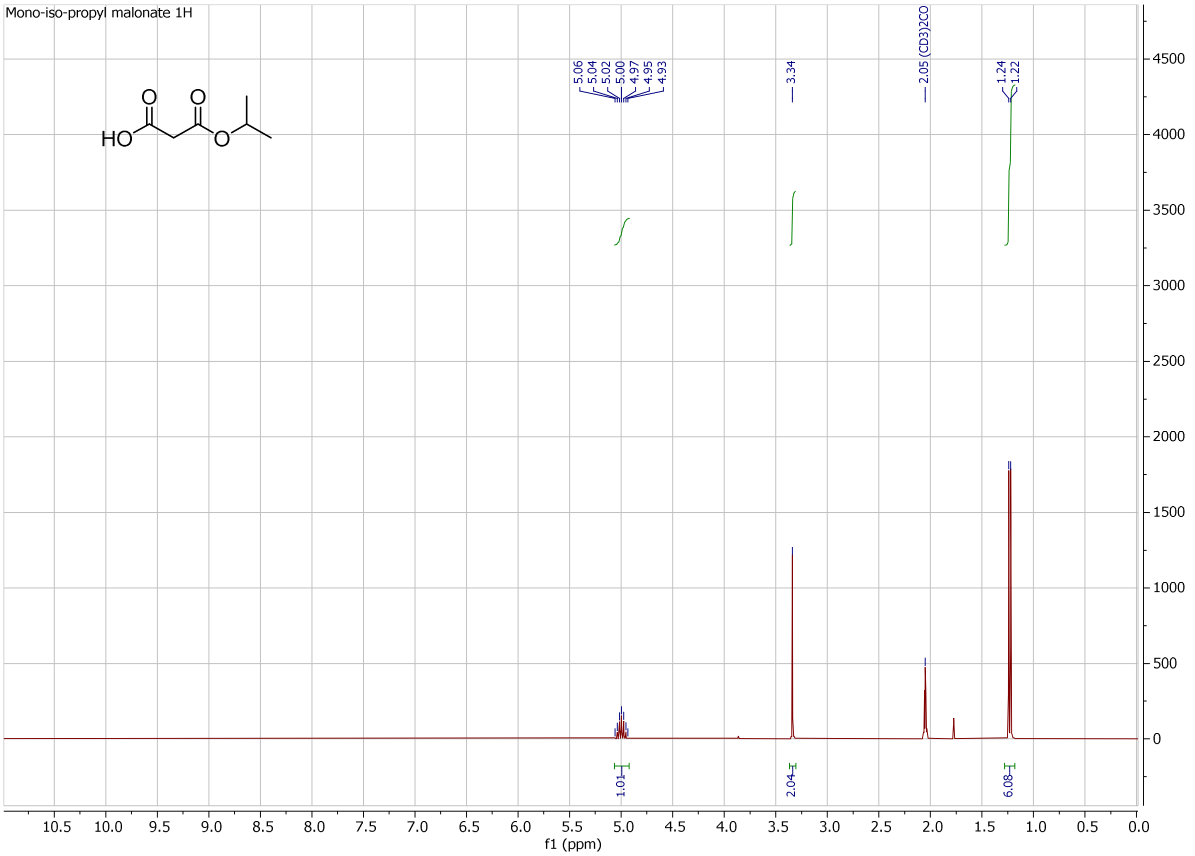
Mono-iso-propyl malonate 1H
5.06
5.04
5.02
5.00
4.97
4.95
4.93
3.34
2.05 (CD3)2CO
1.24
1.22
HO
O
O
O
1.01
2.04
6.08
f1 (ppm)

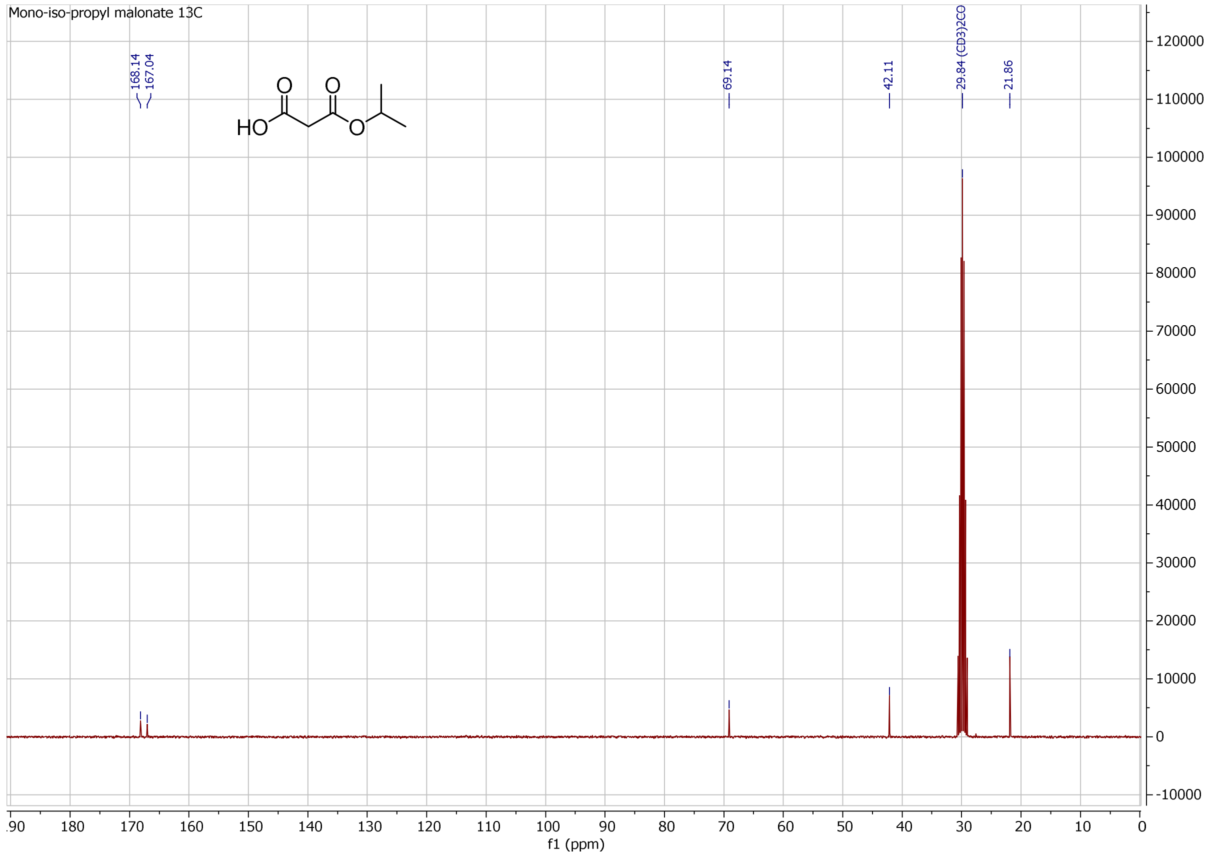
Mono-iso-propyl malonate 13C
168.14
167.04
69.14
42.11
29.84 (CD3)2CO
21.86
HO
O
f1 (ppm)

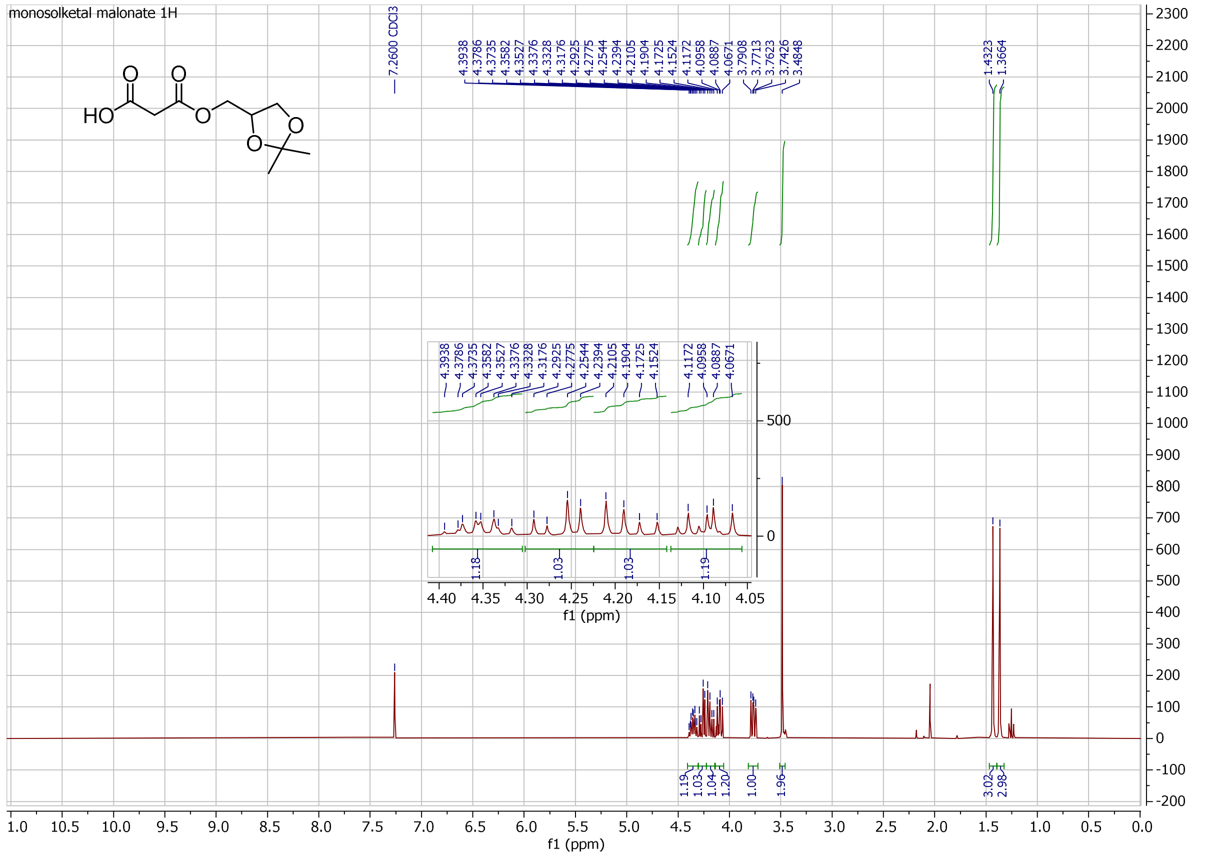
monosolketal malonate 1H
7.2600 CDCl3
1.4323
1.3664
HO
O
f1 (ppm)

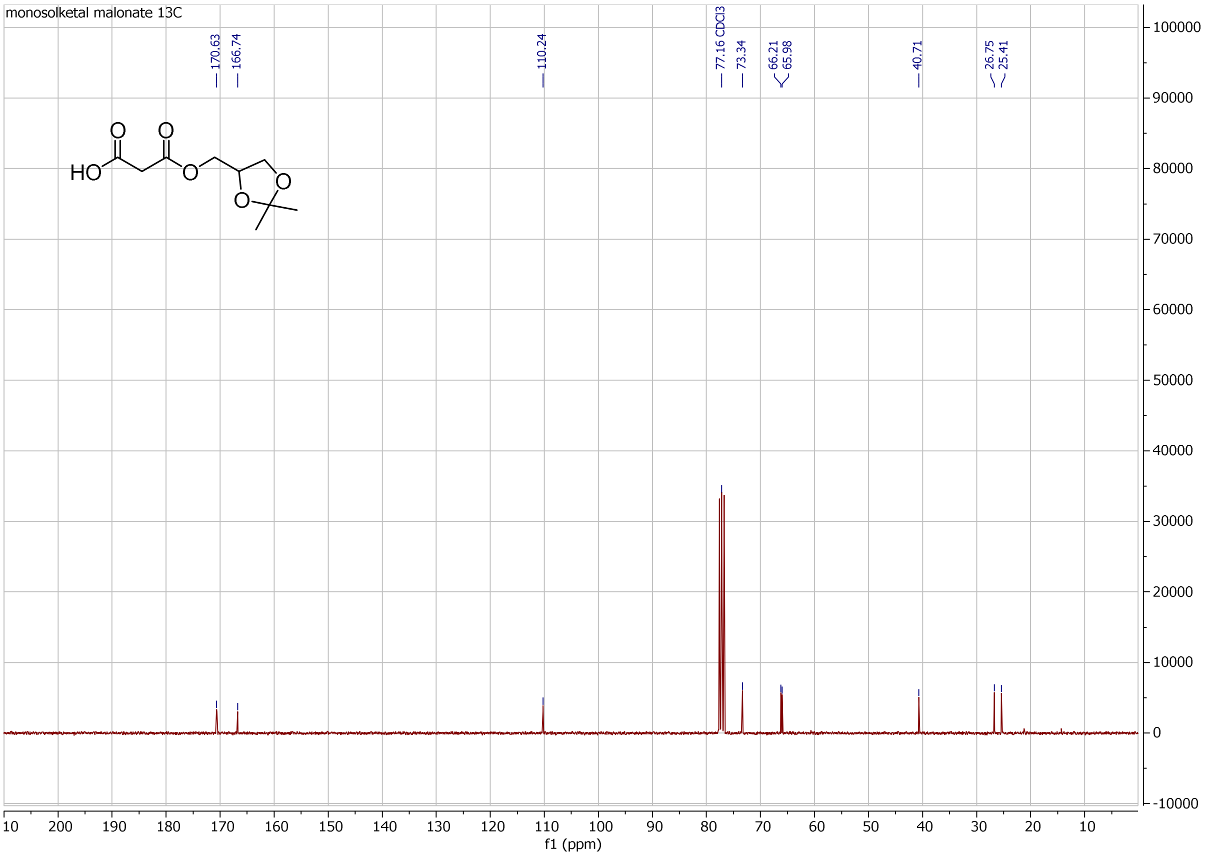
monosolketal malonate 13C
170.63
166.74
110.24
77.16 CDCl3
73.34
66.21
65.98
40.71
26.75
25.41
HO
O
O
O
O
O
f1 (ppm)

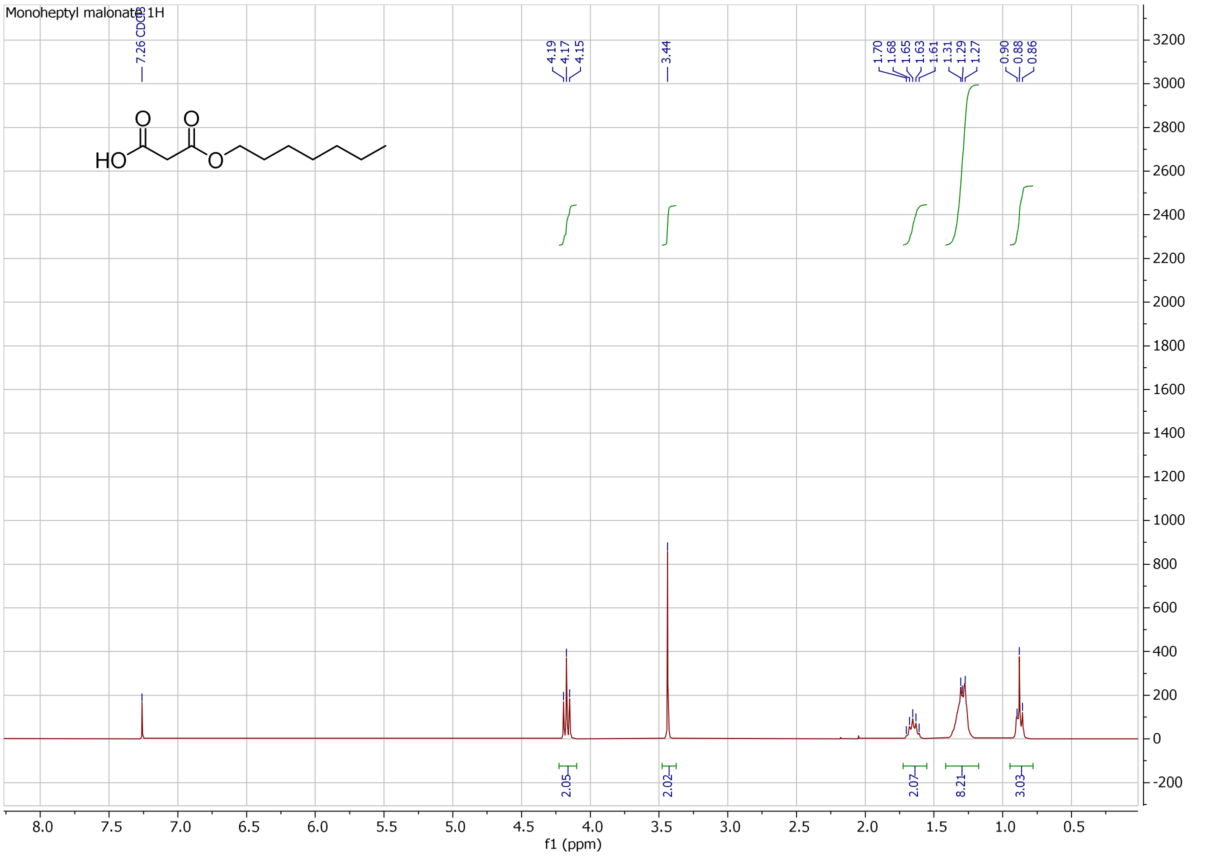
Monoheptyl malonate 1H
7.26 CDCl3
4.19
4.17
4.15
3.44
1.70
1.68
1.65
1.63
1.61
1.31
1.29
1.27
0.90
0.88
0.86
HO
O
O
O
2.05
2.02
2.07
8.21
3.03
f1 (ppm)

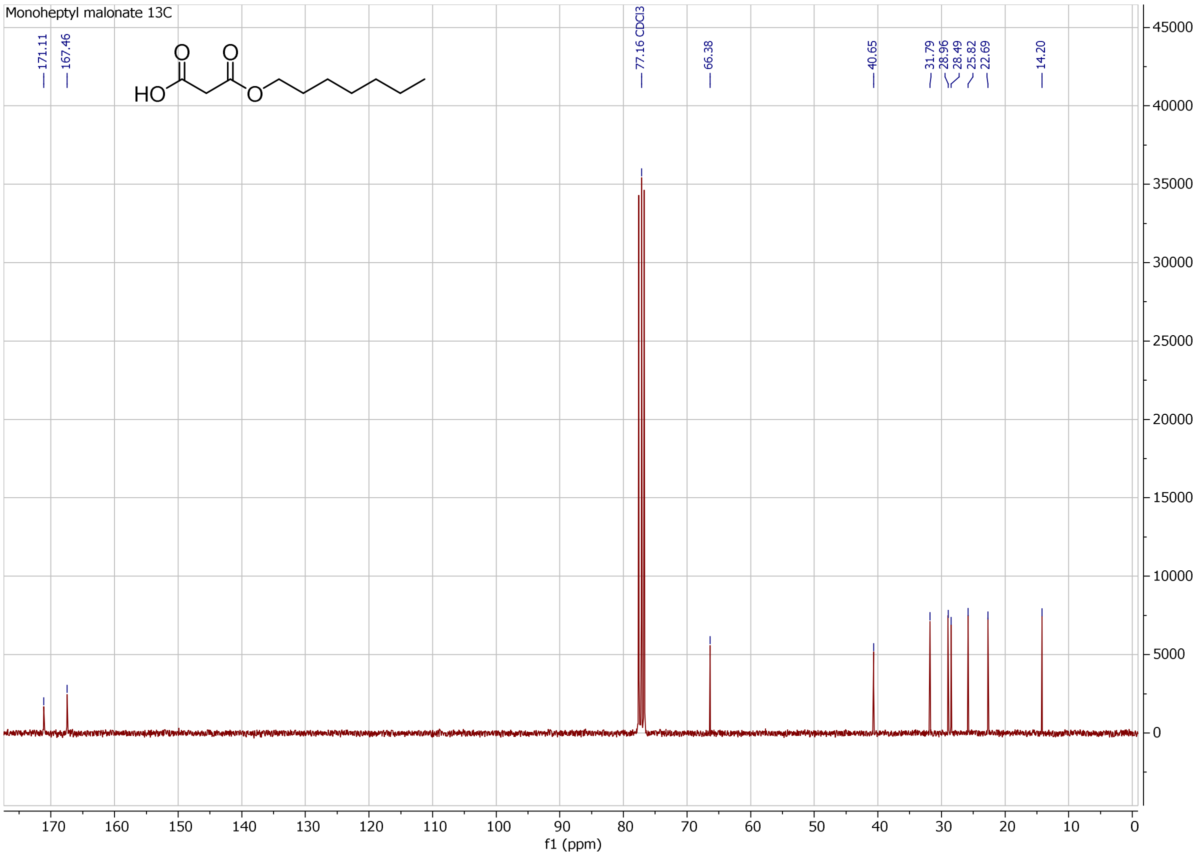
Monoheptyl malonate 13C
171.11
167.46
77.16 CDCl3
66.38
40.65
31.79
28.96
28.49
25.82
22.69
14.20
f1 (ppm)

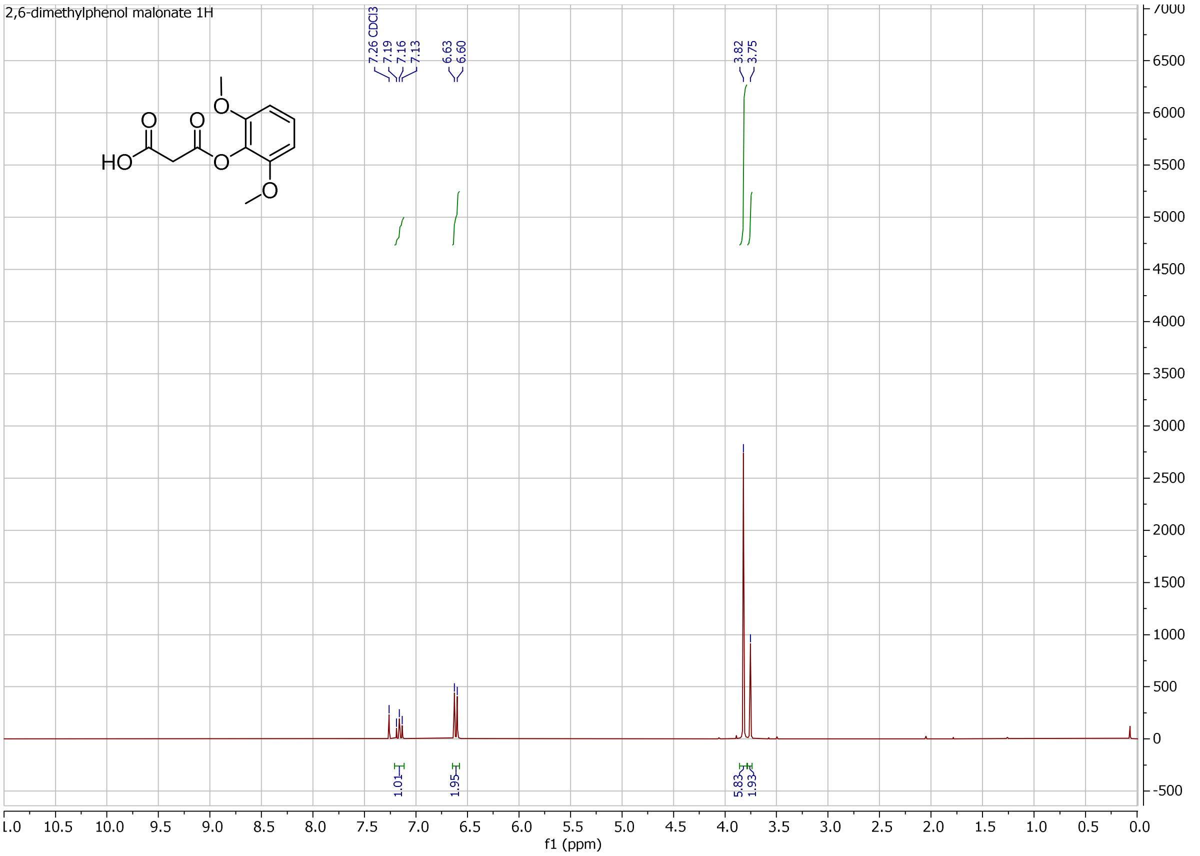
2,6-dimethylphenol malonate 1H
7.26 CDCl3
7.19
7.16
7.13
6.63
6.60
3.82
3.75
1.01
1.95
5.83
1.93
f1 (ppm)

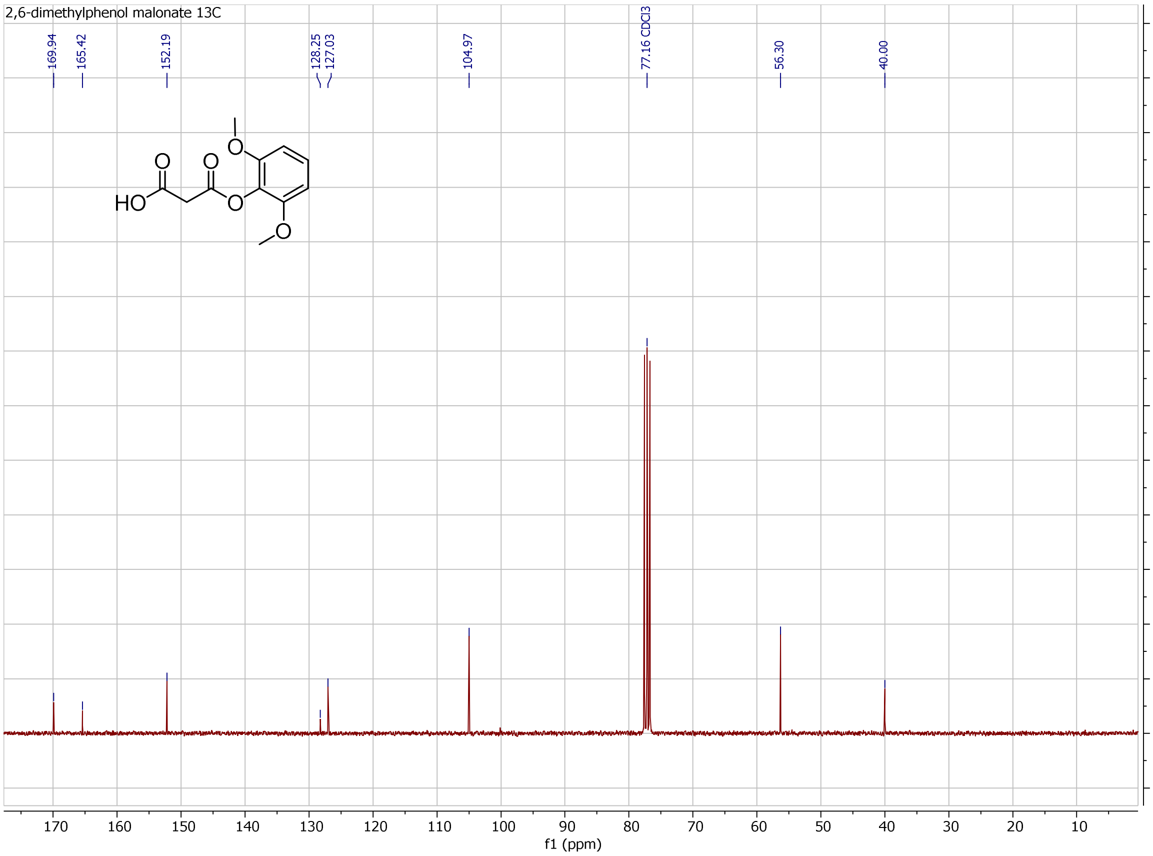
2,6-dimethylphenol malonate 13C
169.94
165.42
152.19
128.25
127.03
104.97
77.16 CDCl3
56.30
40.00
HO
O
f1 (ppm)

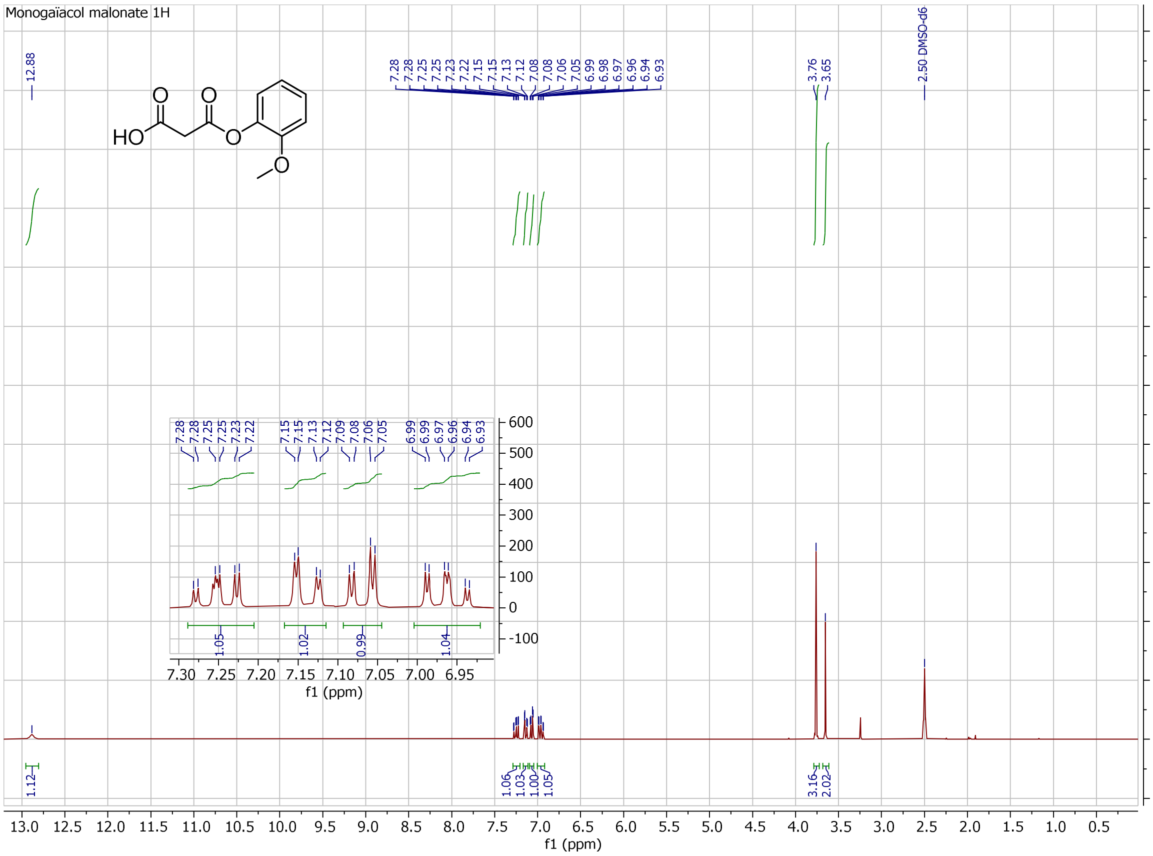
Monogaïacol malonate 1H
12.88
7.28 7.28 7.25 7.25 7.23 7.22 7.15 7.15 7.13 7.12 7.08 7.08 7.06 7.05 6.99 6.98 6.97 6.96 6.94 6.93
3.76
3.65
2.50 DMSO-d6
HO
O
1.05
1.02
0.99
1.04
1.12
1.06
1.03
1.00
1.05
3.16
2.02
f1 (ppm)

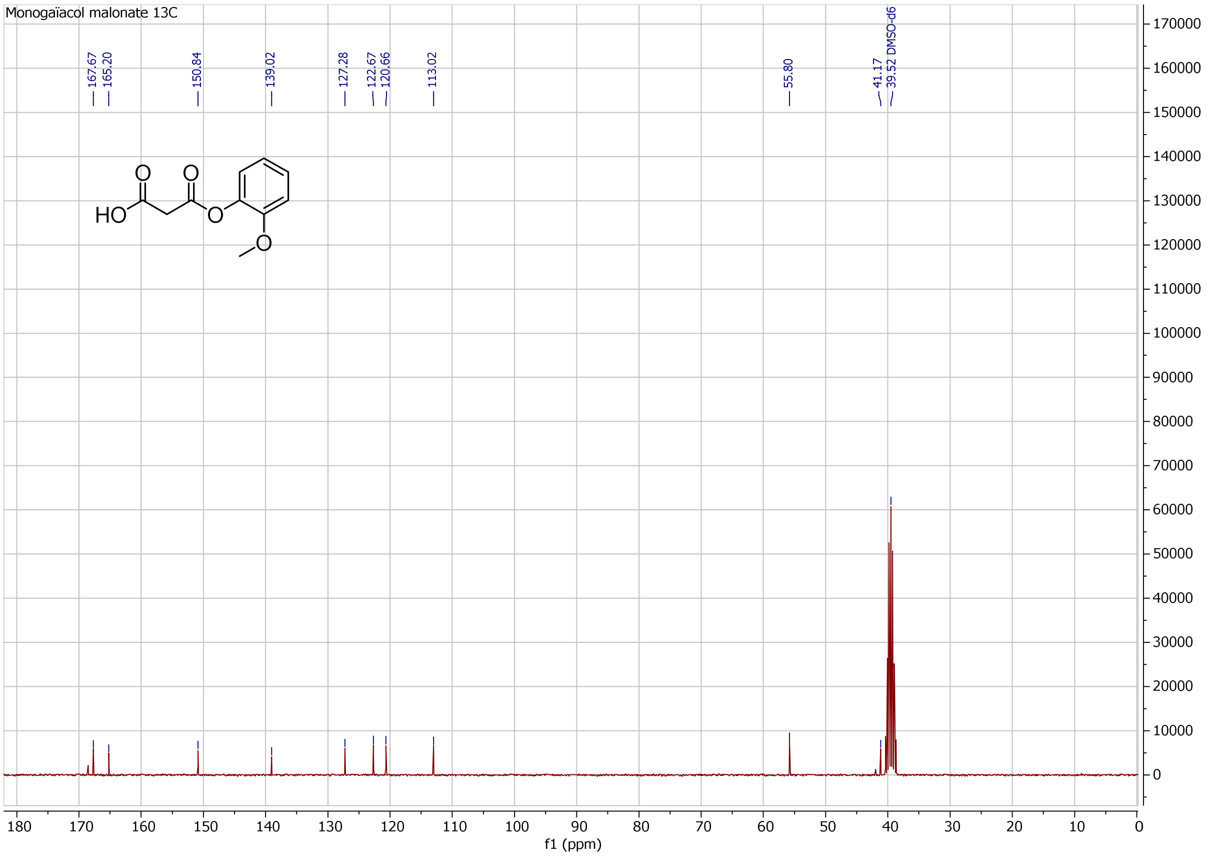
Monogaïacol malonate 13C
167.67
165.20
150.84
139.02
127.28
122.67
120.66
113.02
55.80
41.17
39.52 DMSO-d6
HO
O
O
O
O
f1 (ppm)

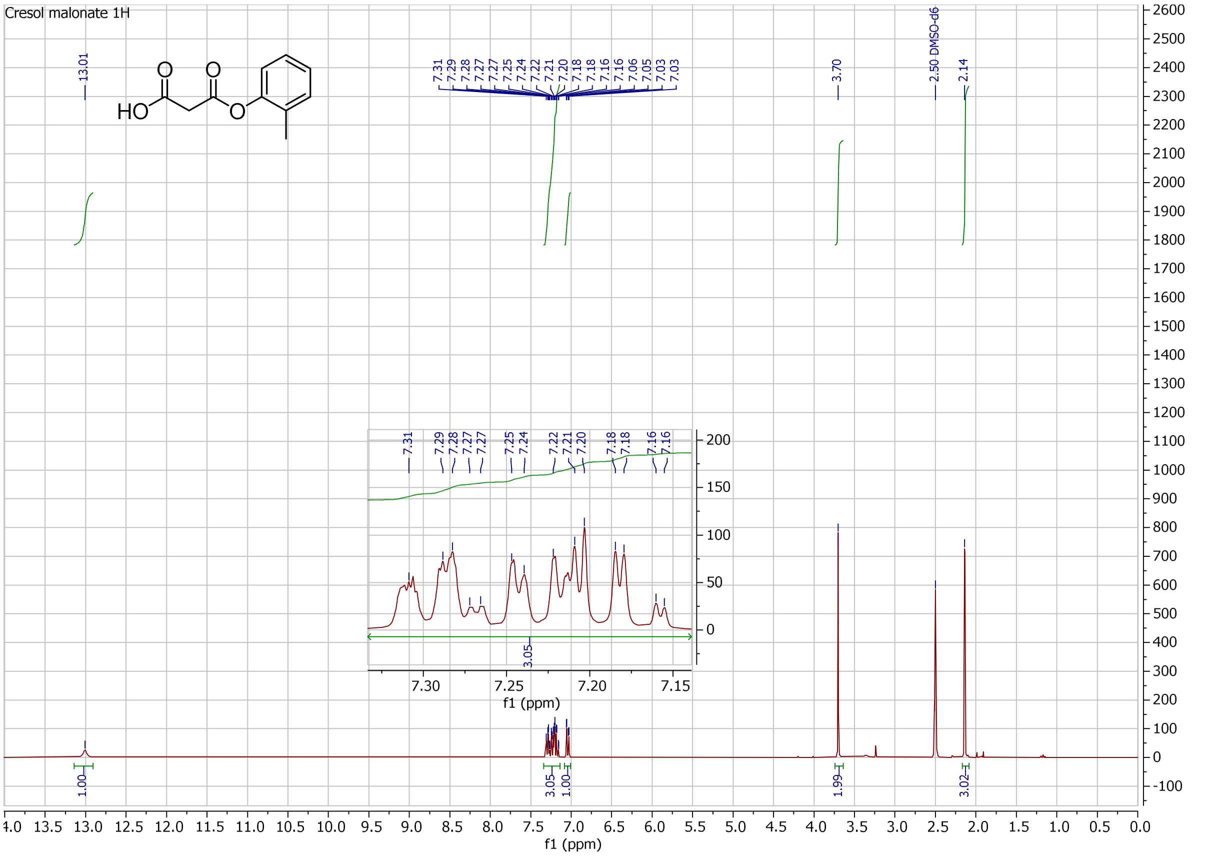
Cresol malonate 1H
13.01
7.31
7.29
7.28
7.27
7.27
7.25
7.24
7.22
7.21
7.20
7.18
7.16
7.16
7.06
7.05
7.03
7.03
3.70
2.50 DMSO-d6
2.14
HO
O
O
O
1.00
3.05
1.00
1.99
3.02
f1 (ppm)

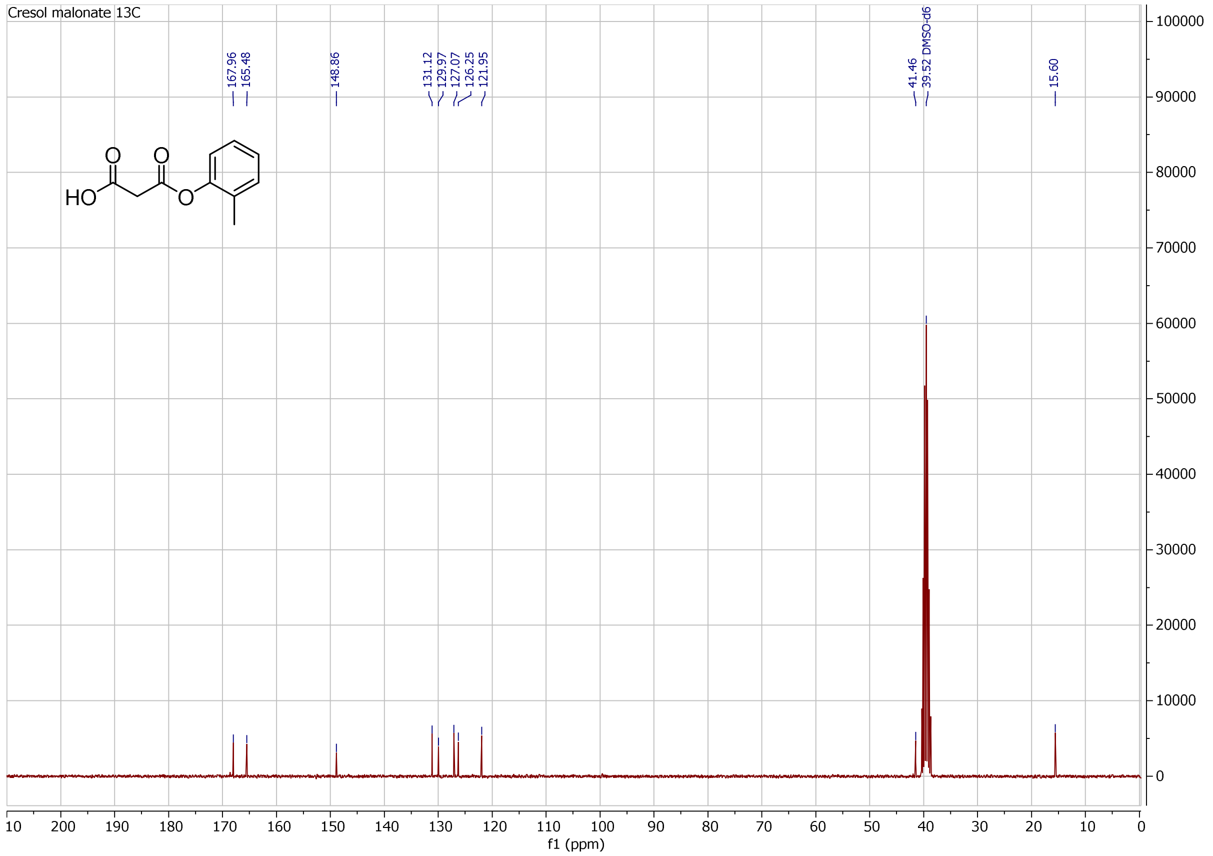

Cresol malonate 13C
167.96
165.48
148.86
131.12
129.97
127.07
126.25
121.95
41.46
39.52 DMSO-d6
15.60
HO
O
O
O
f1 (ppm)

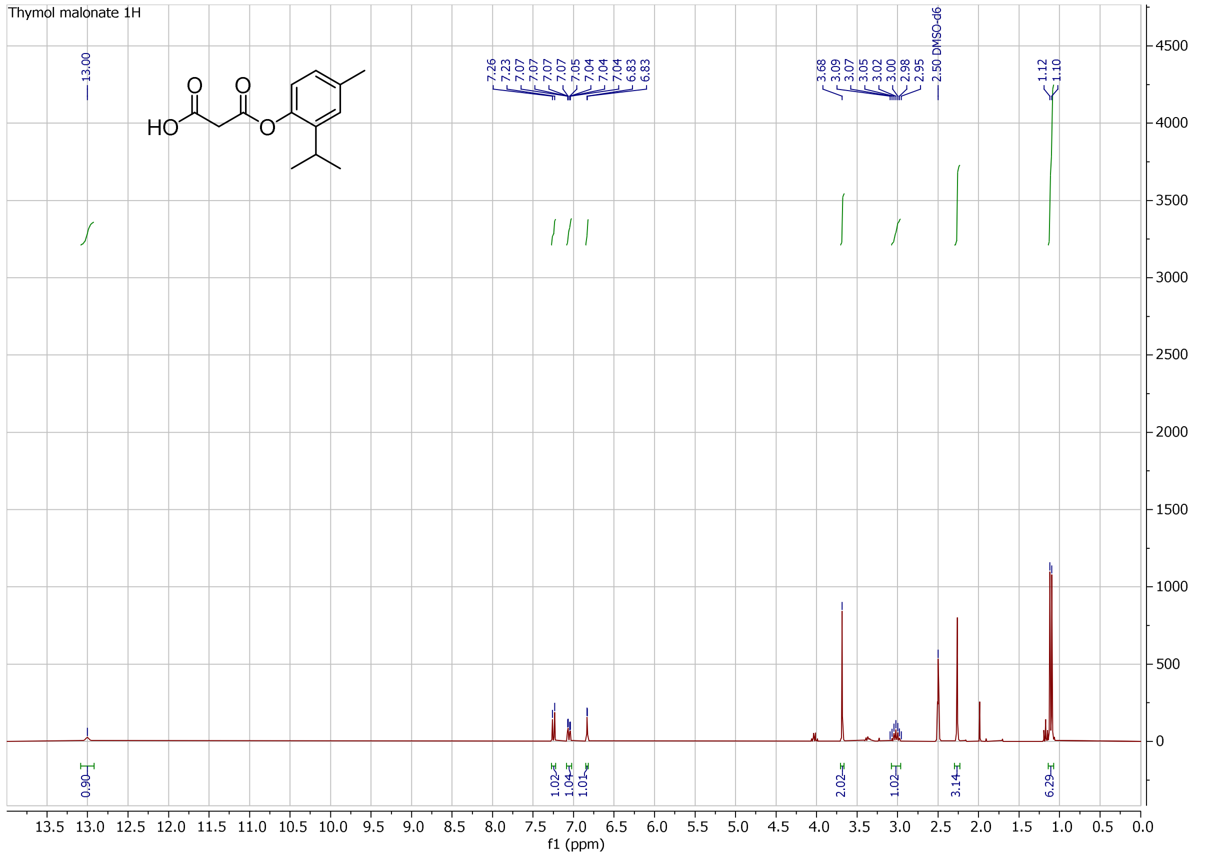

Thymol malonate 1H
13.00
7.26
7.23
7.07
7.07
7.07
7.05
7.04
7.04
7.04
6.83
6.83
3.68
3.09
3.07
3.05
3.02
3.00
2.98
2.95
2.50 DMSO-d6
1.12
1.10
HO
O
O
O
0.90
1.02
1.04
1.01
2.02
1.02
3.14
6.29
f1 (ppm)

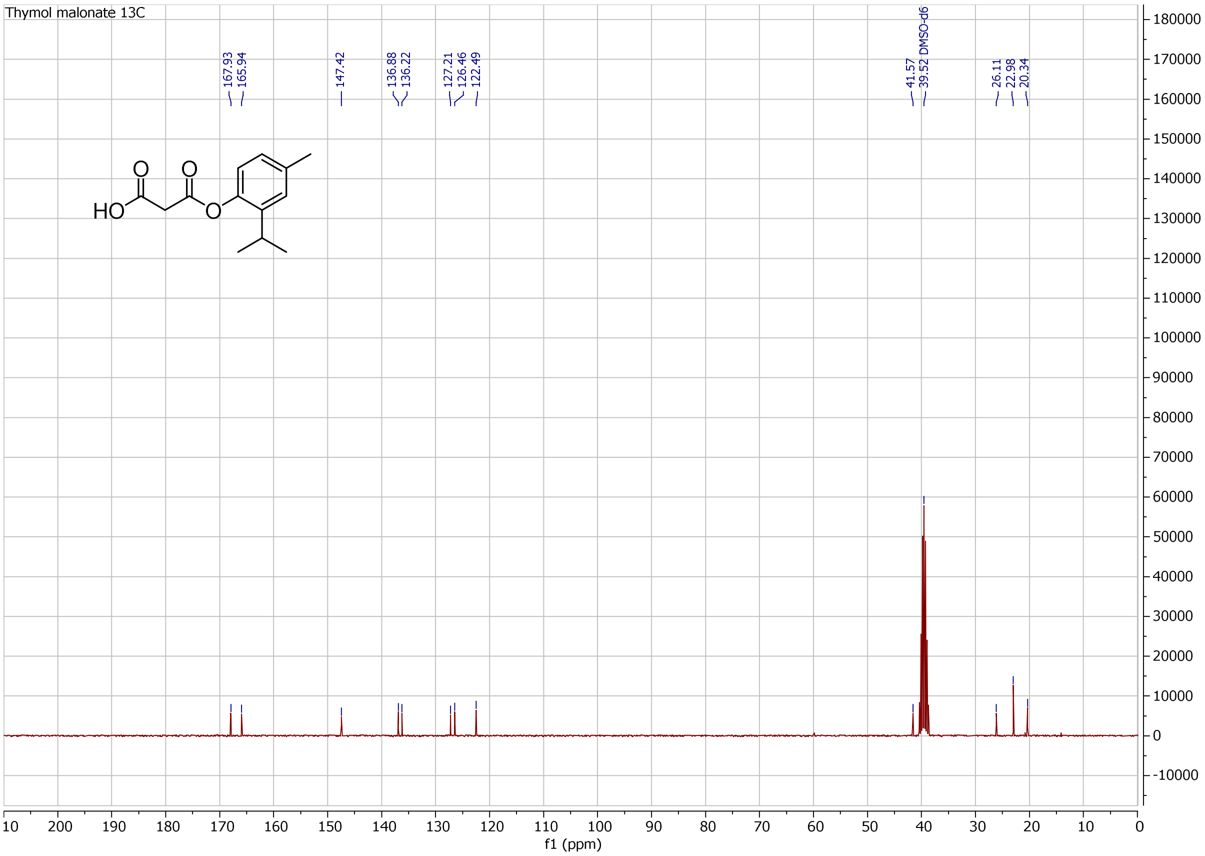
Thymol malonate 13C
167.93
165.94
147.42
136.88
136.22
127.21
126.46
122.49
41.57
39.52 DMSO-d6
26.11
22.98
20.34
HO
O
f1 (ppm)

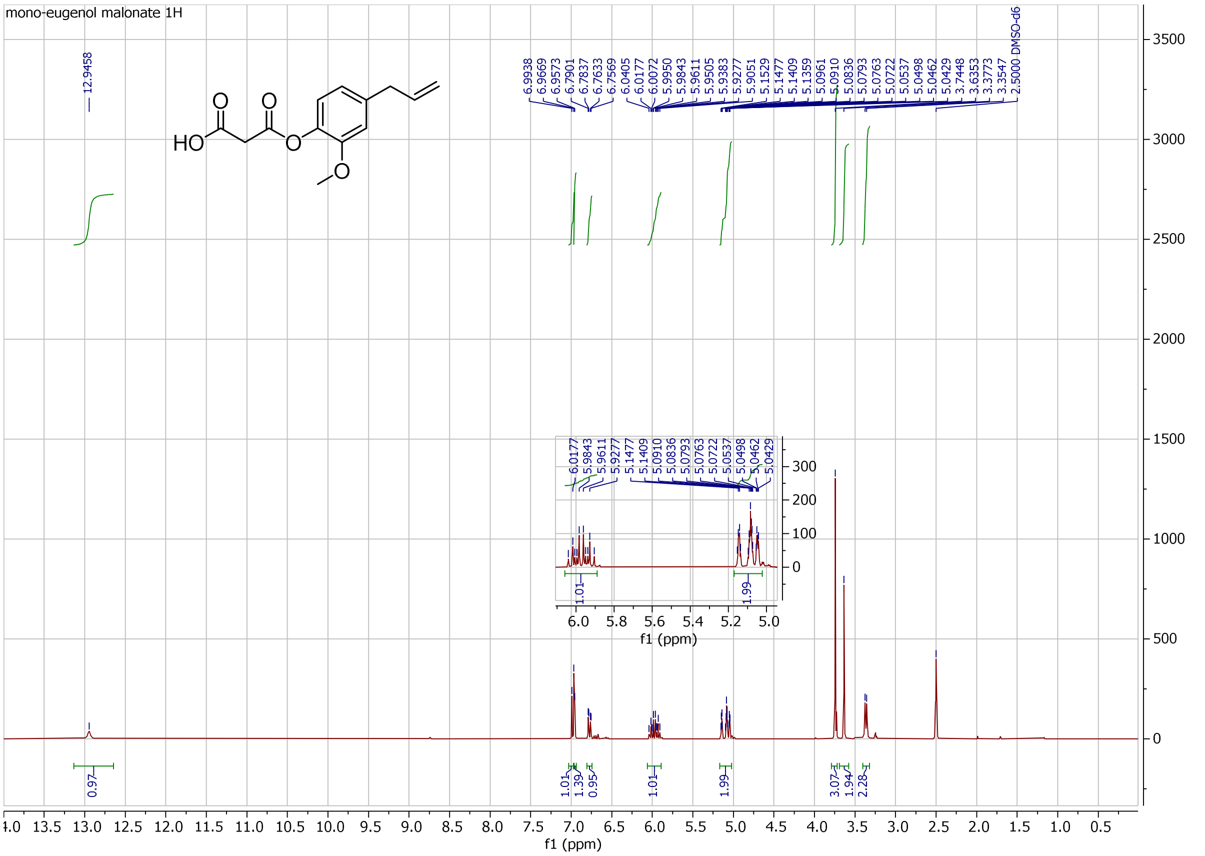
mono-eugenol malonate 1H
12.9458
HO
O
2.5000 DMSO-d6
0.97
1.01
1.39
0.95
1.01
1.99
3.07
1.94
2.28
f1 (ppm)

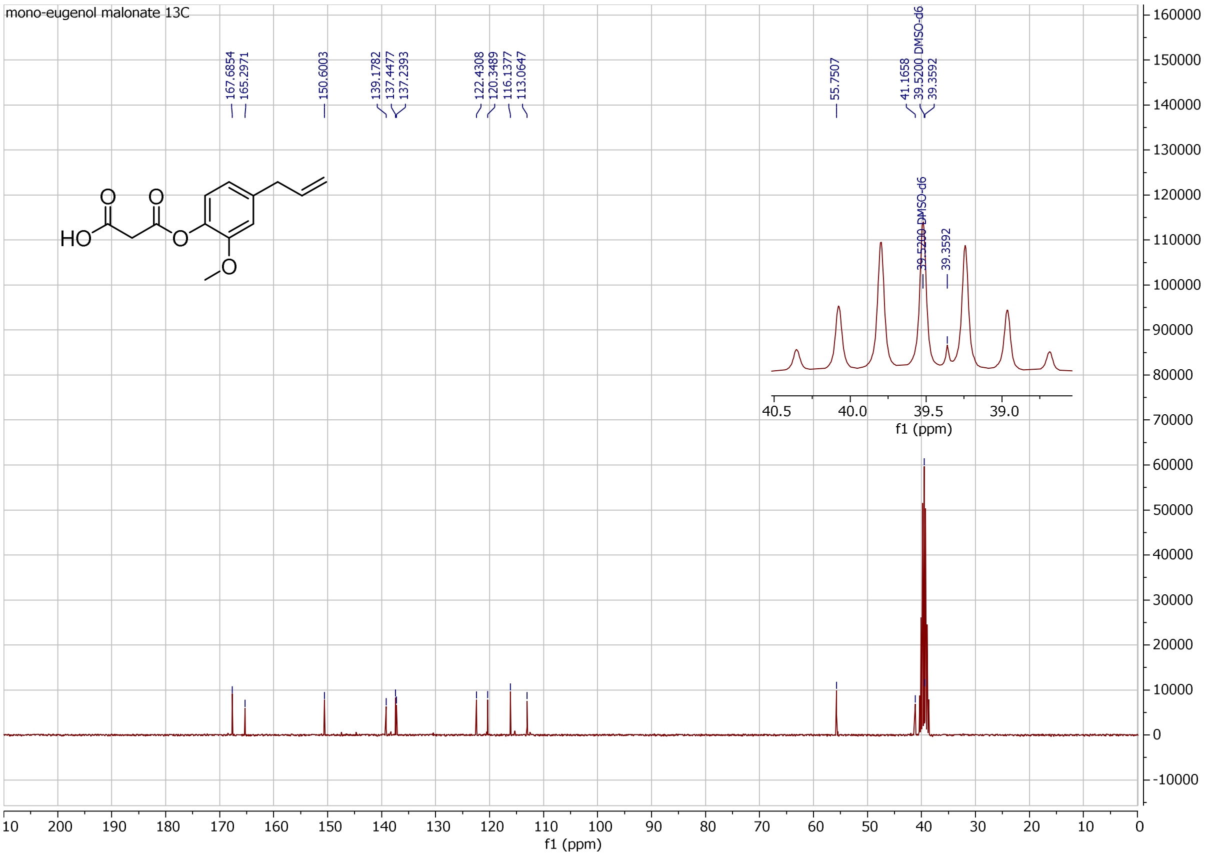

3.2. Sinapate esters

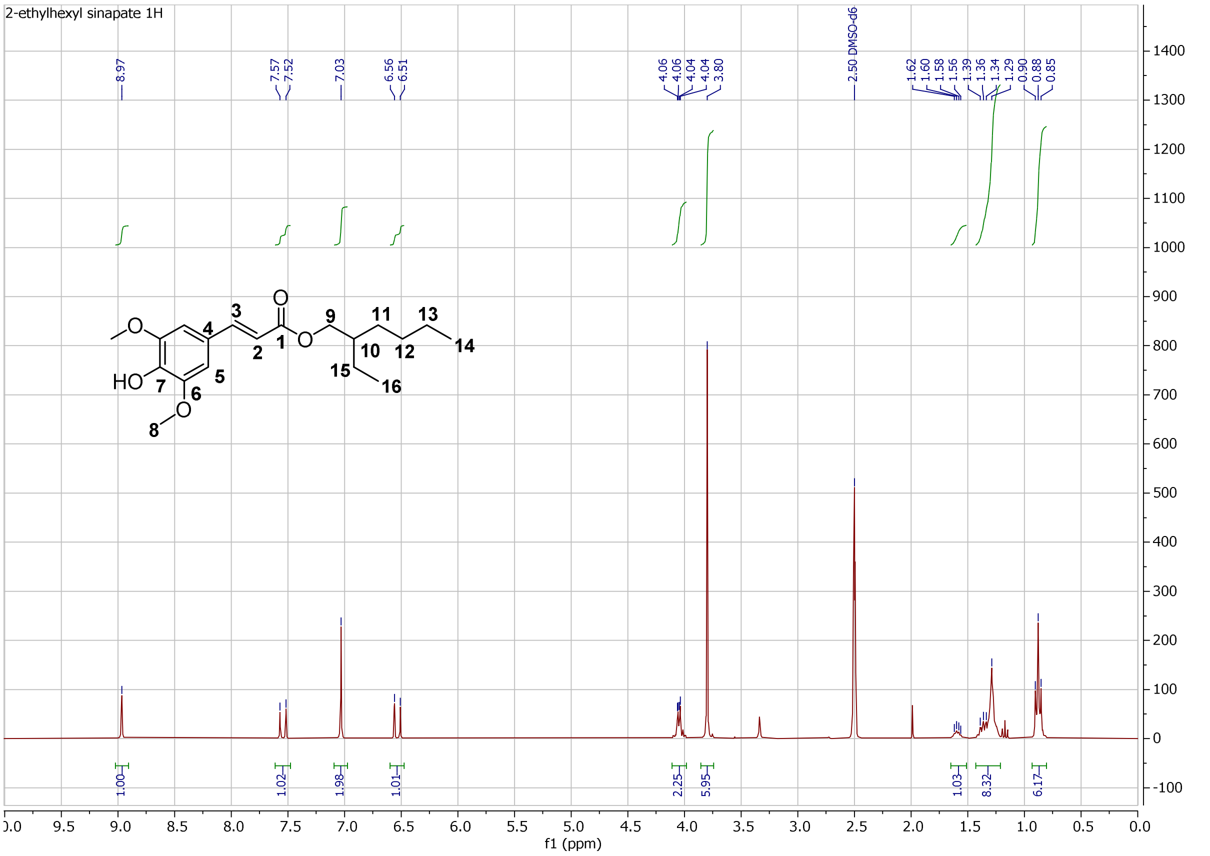

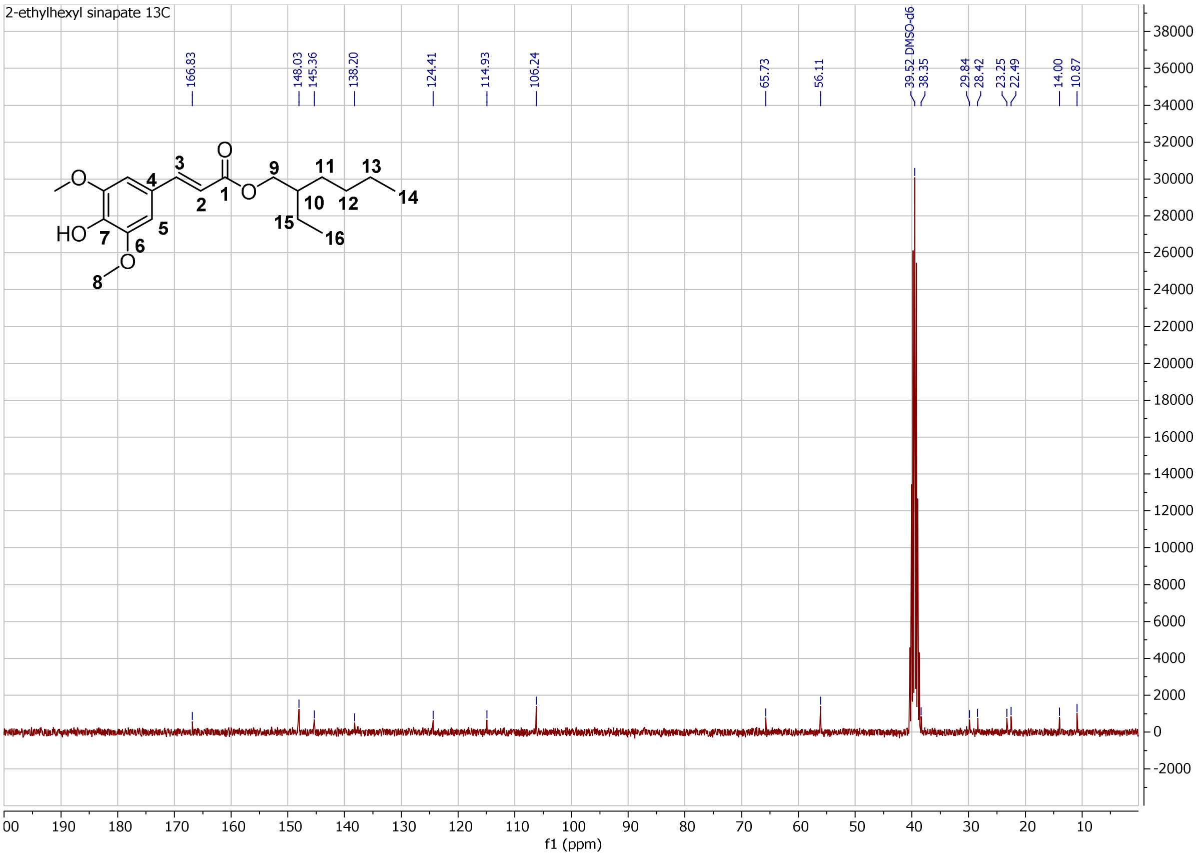
2-ethylhexyl sinapate 13C
166.83
148.03
145.36
138.20
124.41
114.93
106.24
65.73
56.11
39.52 DMSO-d6
38.35
29.84
28.42
23.25
22.49
14.00
10.87
f1 (ppm)

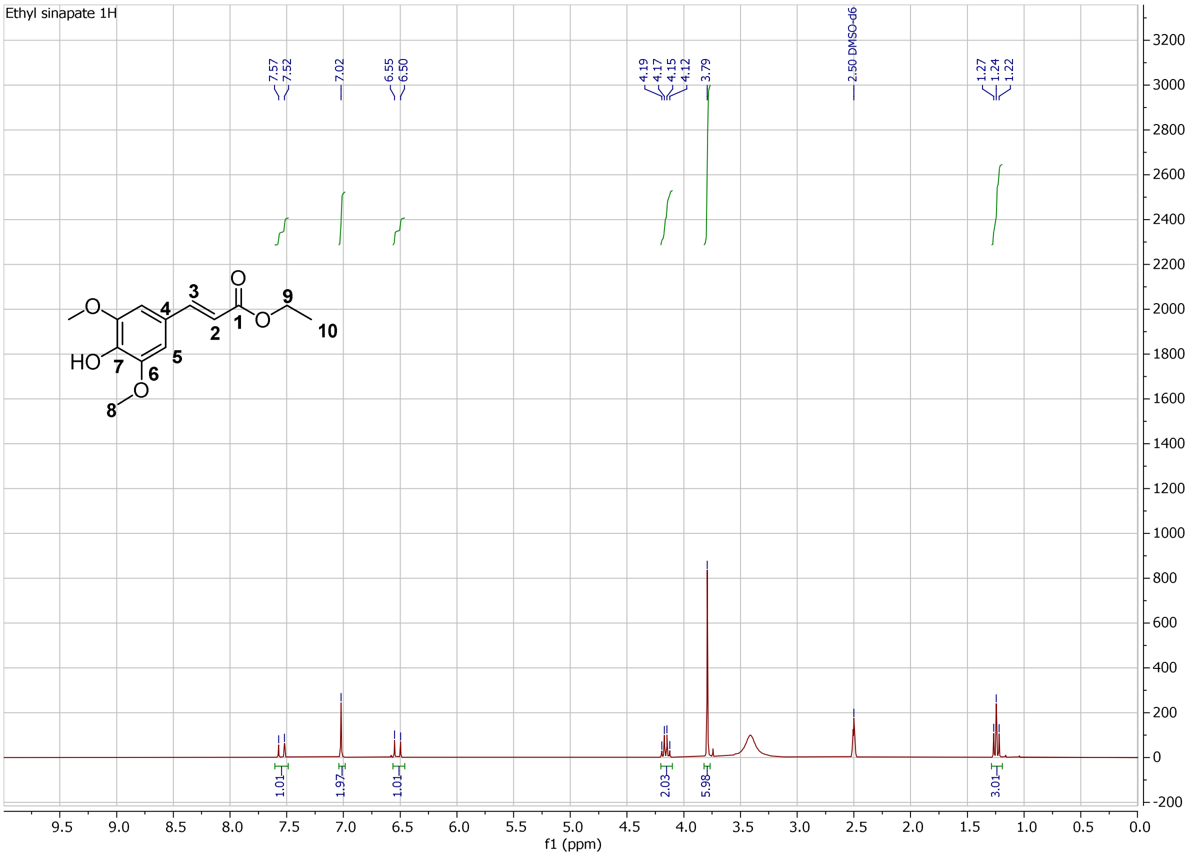
Ethyl sinapate 1H
7.57
7.52
7.02
6.55
6.50
4.19
4.17
4.15
4.12
3.79
2.50 DMSO-d6
1.27
1.24
1.22
1.01
1.97
1.01
2.03
5.98
3.01
f1 (ppm)

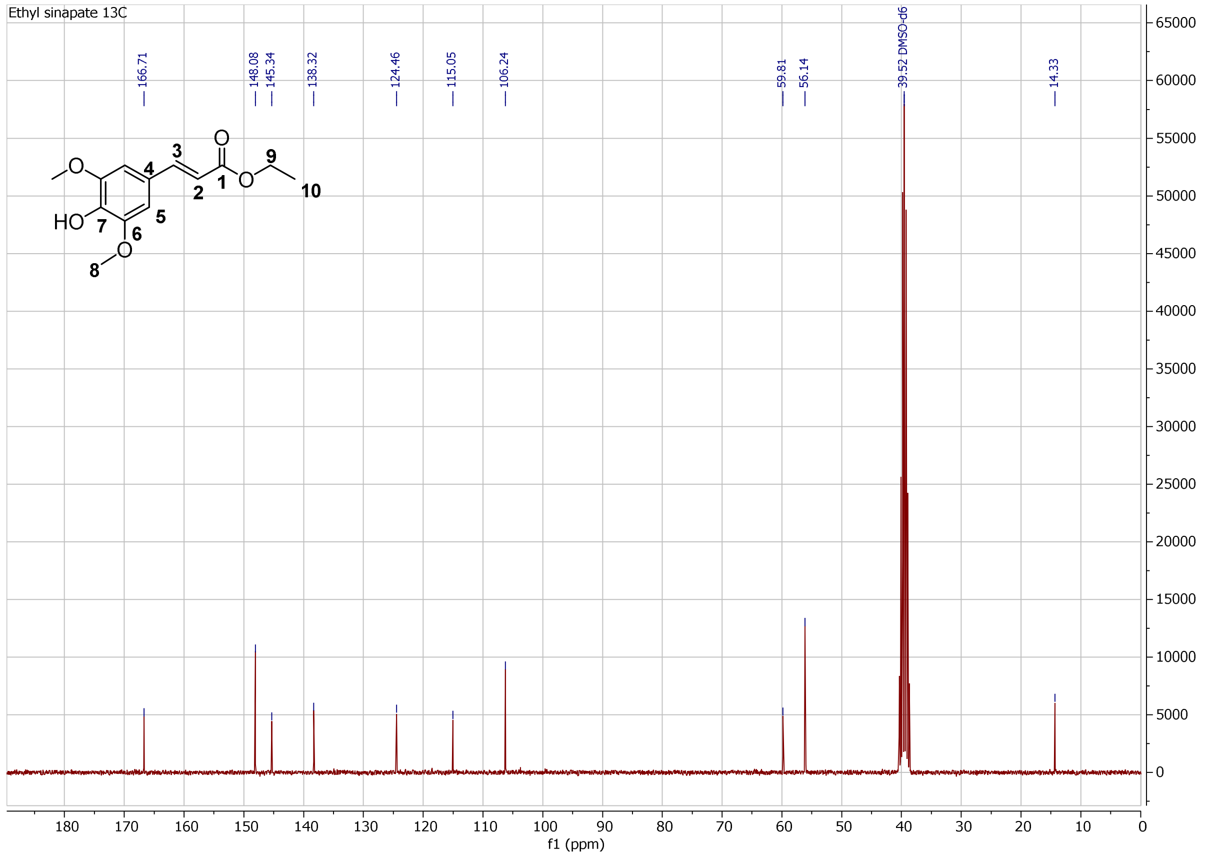

Ethyl sinapate 13C
166.71
148.08
145.34
138.32
124.46
115.05
106.24
59.81
56.14
39.52 DMSO-d6
14.33
f1 (ppm)

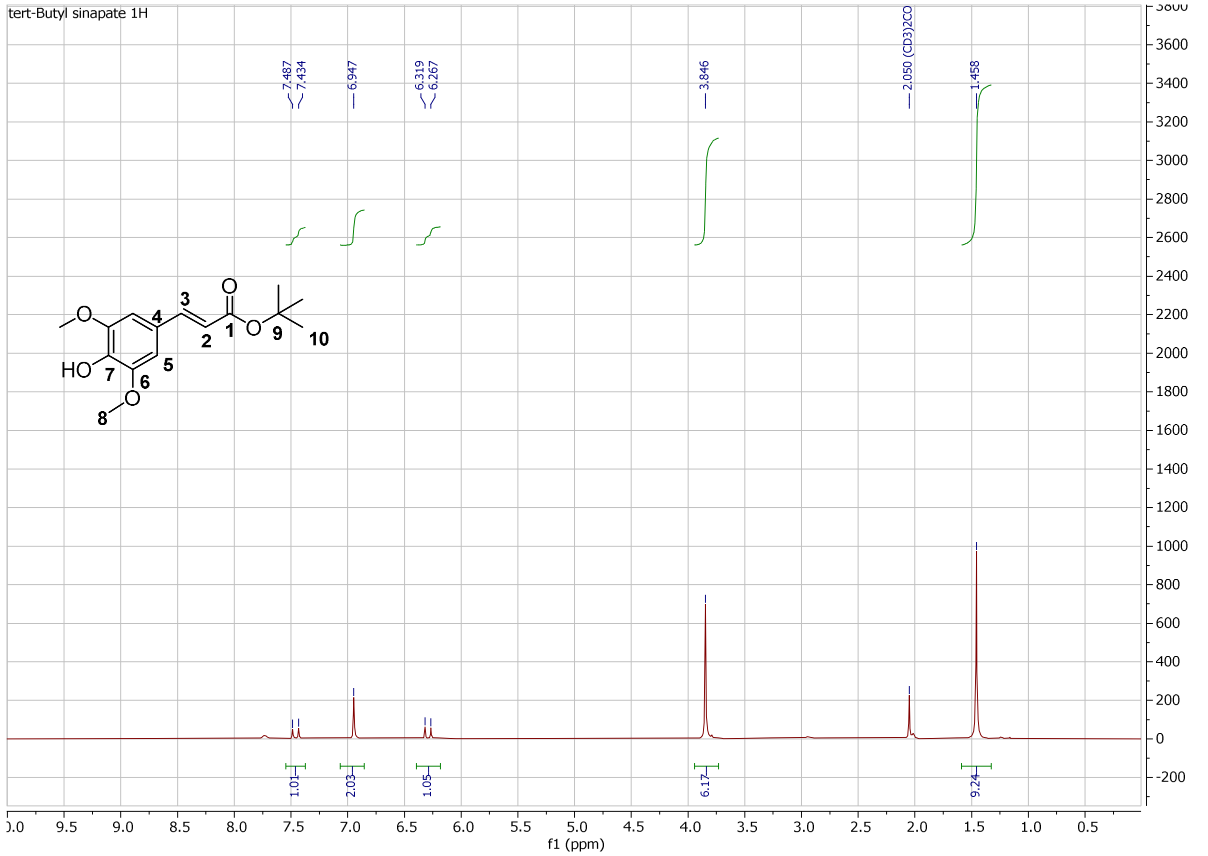

tert-Butyl sinapate 1H
7.487
7.434
6.947
6.319
6.267
3.846
2.050 (CD3)2CO
1.458
1.01
2.03
1.05
6.17
9.24
f1 (ppm)

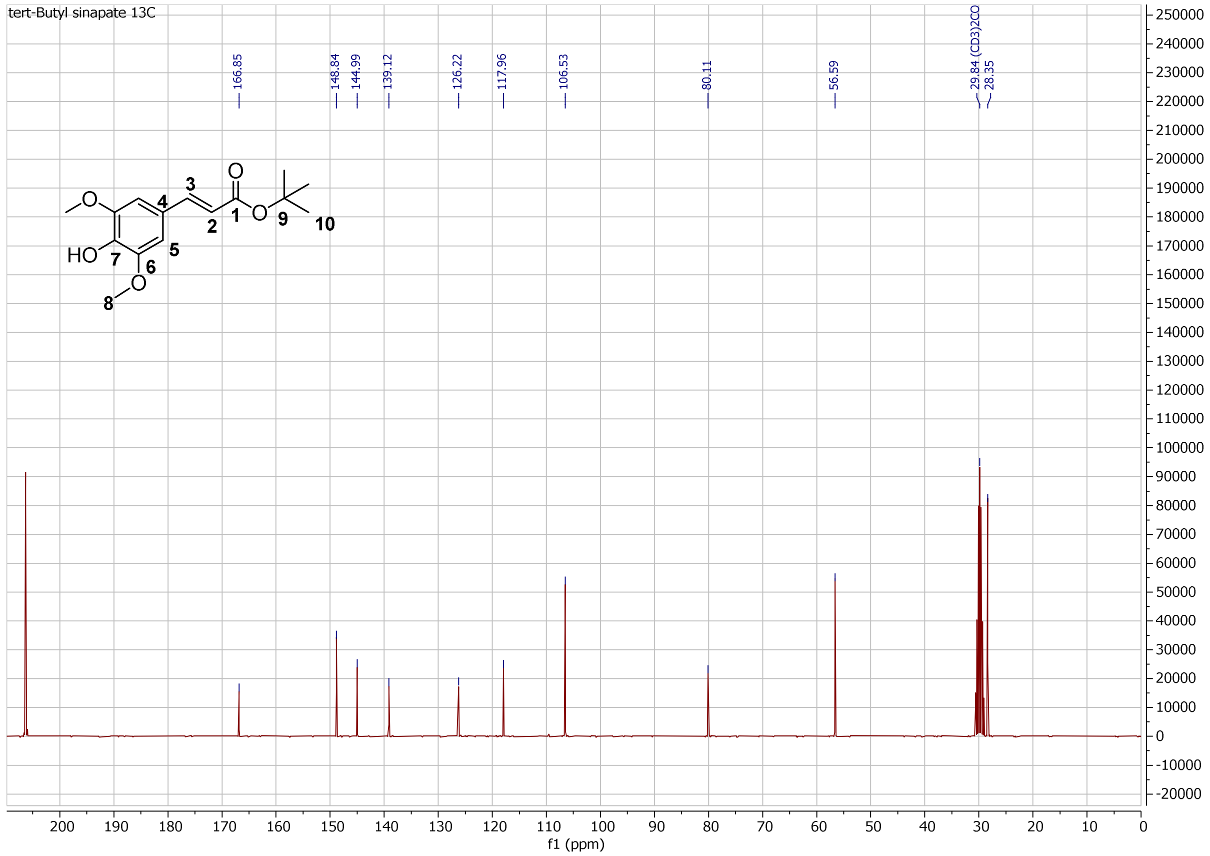
tert-Butyl sinapate 13C
166.85
148.84
144.99
139.12
126.22
117.96
106.53
80.11
56.59
29.84 (CD3)2CO
28.35
f1 (ppm)

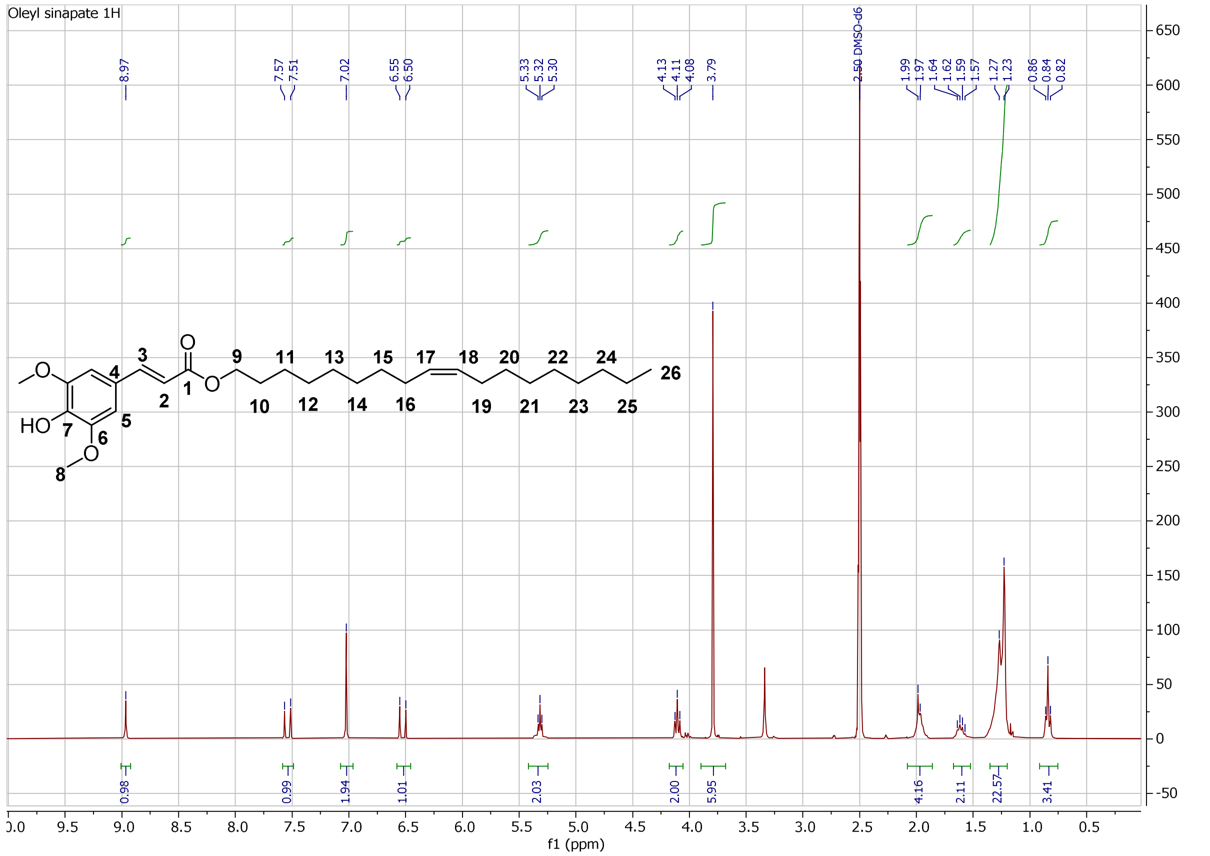
Oleyl sinapate 1H
8.97
7.57
7.51
7.02
6.55
6.50
5.33
5.32
5.30
4.13
4.11
4.08
3.79
2.50 DMSO-d6
1.99
1.97
1.64
1.62
1.59
1.57
1.27
1.23
0.86
0.84
0.82
0.98
0.99
1.94
1.01
2.03
2.00
5.95
4.16
2.11
22.57
3.41
f1 (ppm)

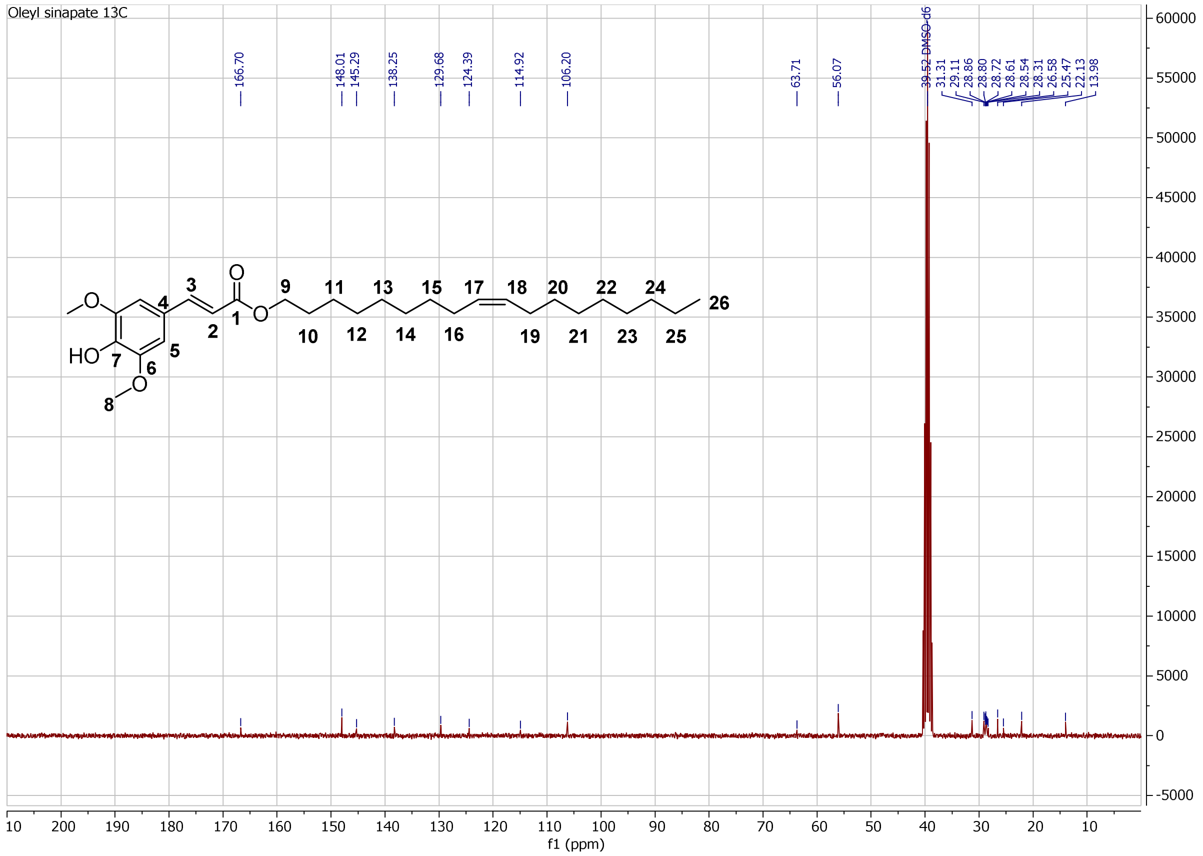
Oleyl sinapate 13C
166.70
148.01
145.29
138.25
129.68
124.39
114.92
106.20
63.71
56.07
39.52 DMSO-d6
31.31
29.11
28.86
28.80
28.72
28.61
28.54
28.31
26.58
25.47
22.13
13.98
f1 (ppm)

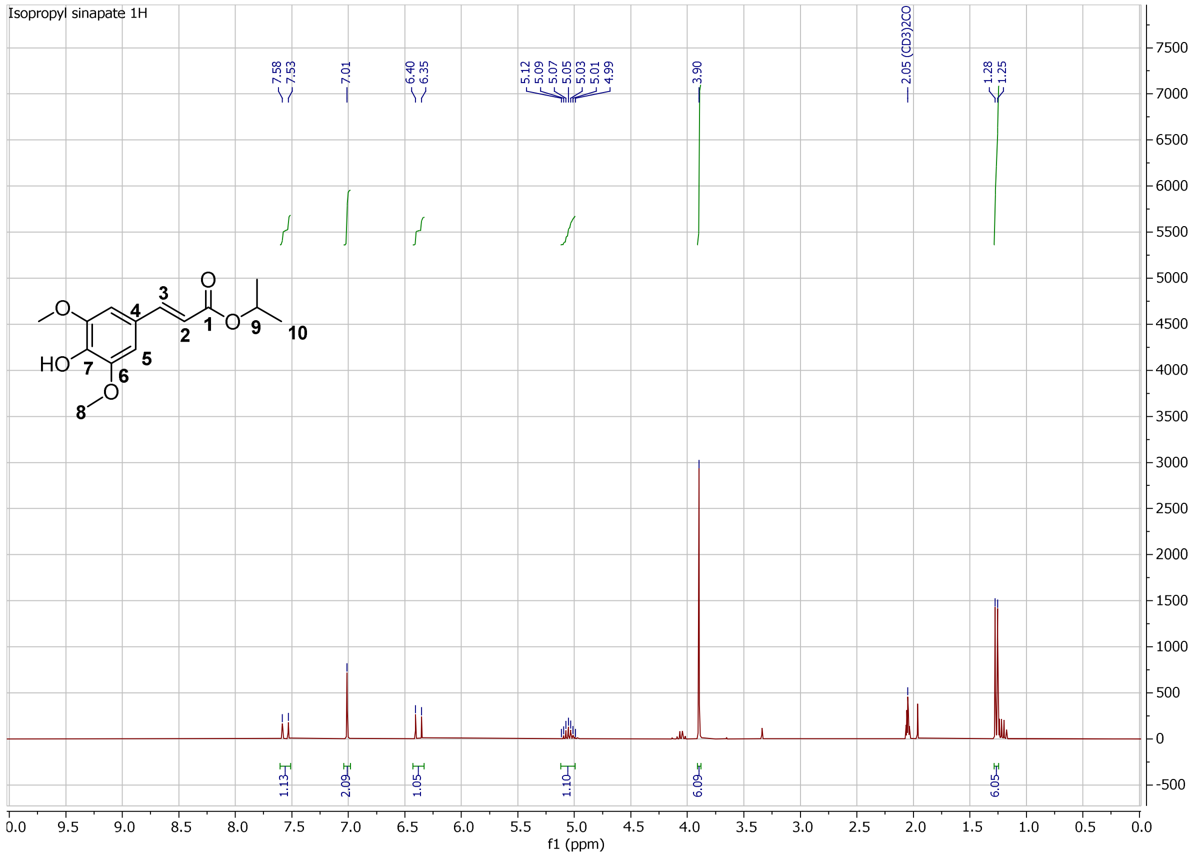
Isopropyl sinapate 1H
7.58
7.53
7.01
6.40
6.35
5.12
5.09
5.07
5.05
5.03
5.01
4.99
3.90
2.05 (CD3)2CO
1.28
1.25
1.13
2.09
1.05
1.10
6.09
6.05
f1 (ppm)

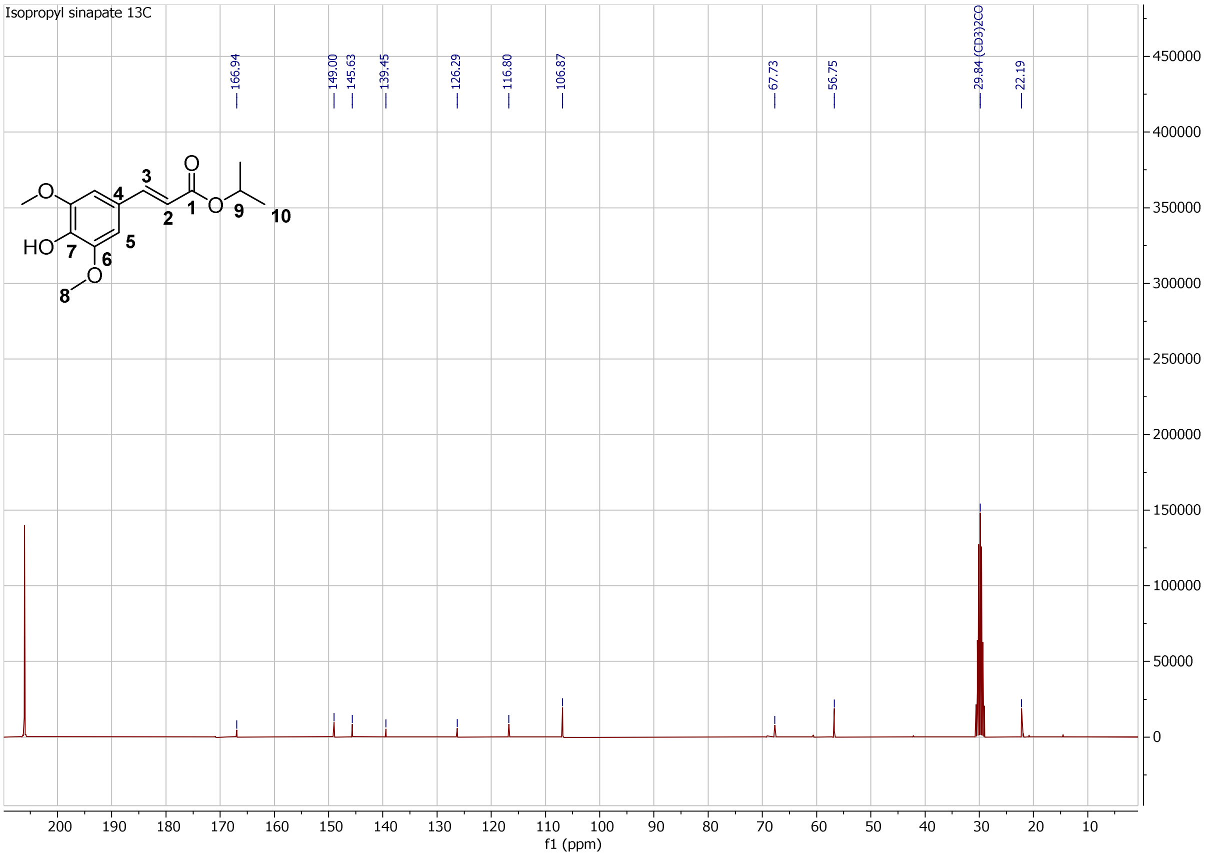
Isopropyl sinapate 13C
166.94
149.00
145.63
139.45
126.29
116.80
106.87
67.73
56.75
29.84 (CD3)2CO
22.19
f1 (ppm)

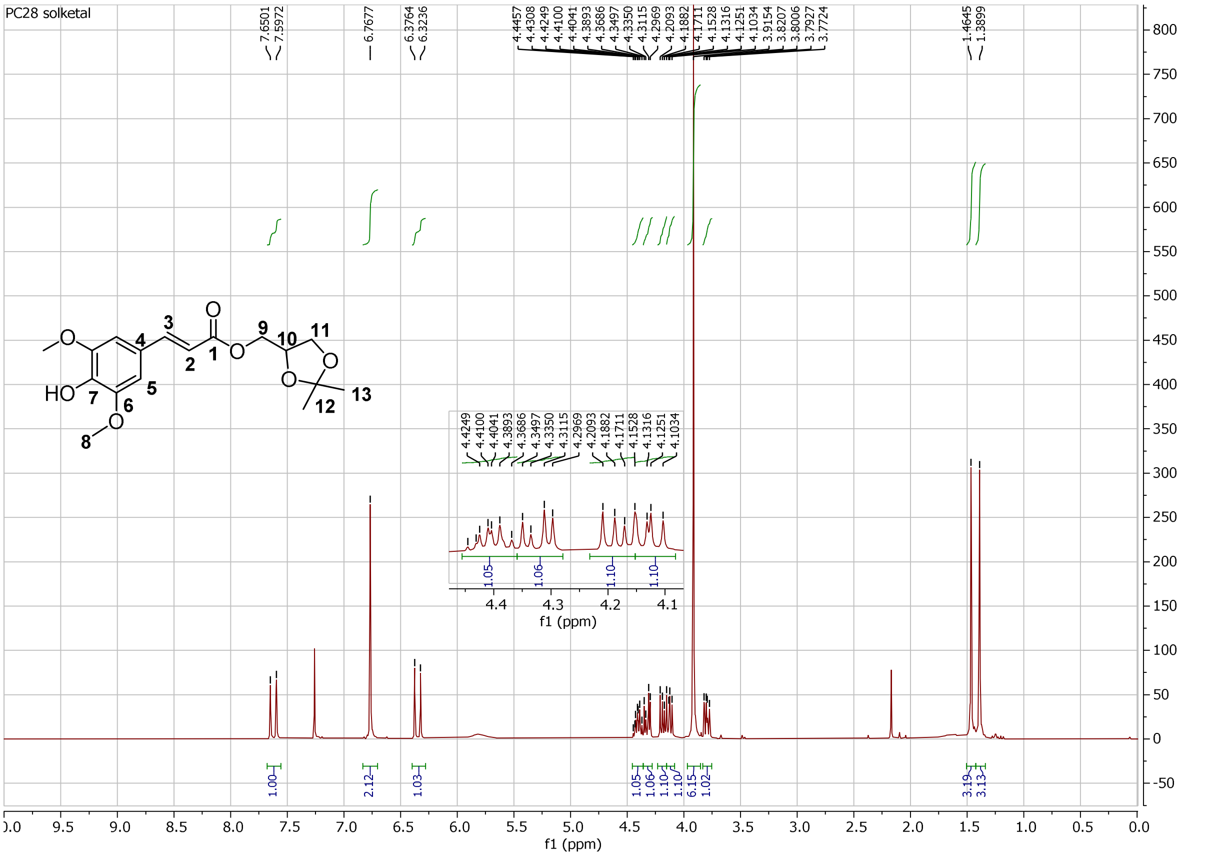
PC28 solketal
7.6501
7.5972
6.7677
6.3764
6.3236
1.4645
1.3899
f1 (ppm)

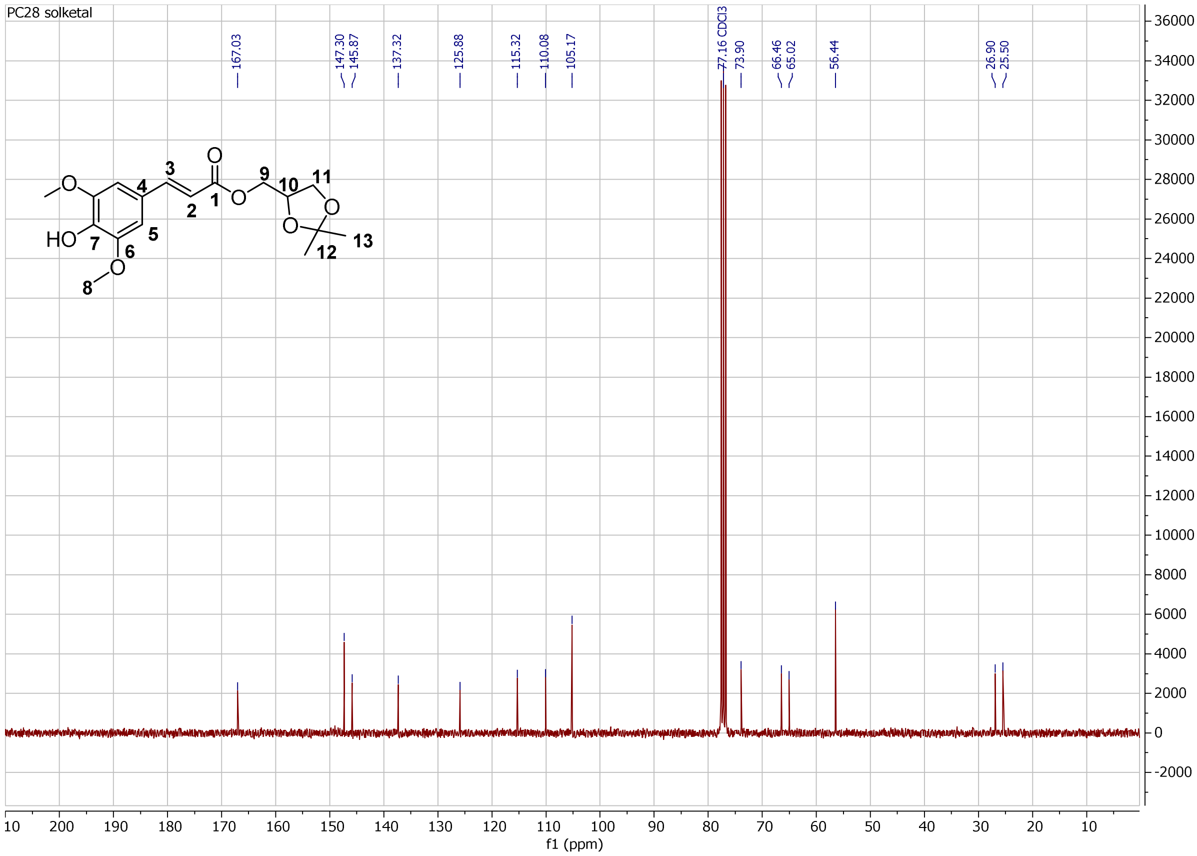
PC28 solketal
167.03
147.30
145.87
137.32
125.88
115.32
110.08
105.17
77.16 CDCl3
73.90
66.46
65.02
56.44
26.90
25.50
f1 (ppm)

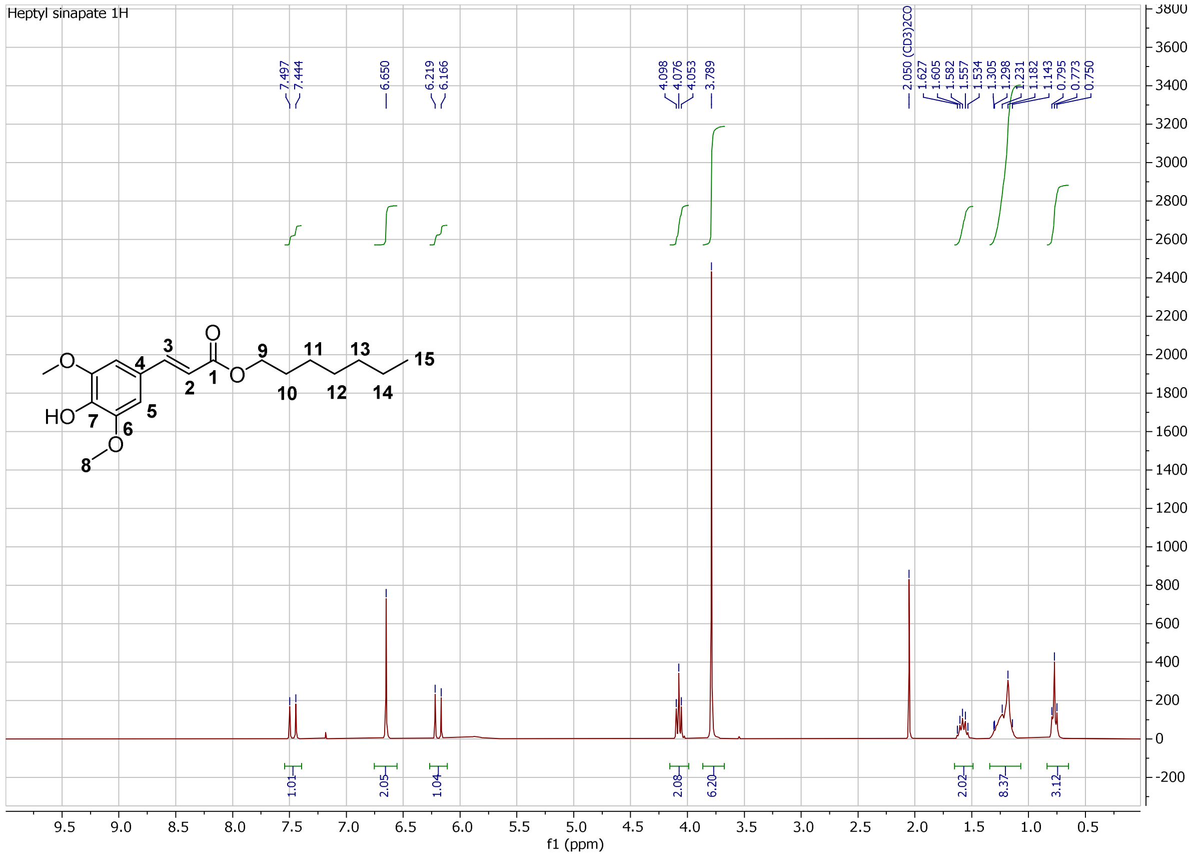
Heptyl sinapate 1H
7.497
7.444
6.650
6.219
6.166
4.098
4.076
4.053
3.789
2.050 (CD3)2CO
1.627
1.605
1.582
1.557
1.534
1.305
1.298
1.231
1.182
1.143
0.795
0.773
0.750
1.01
2.05
1.04
2.08
6.20
2.02
8.37
3.12
f1 (ppm)

Heptyl sinapate 13C

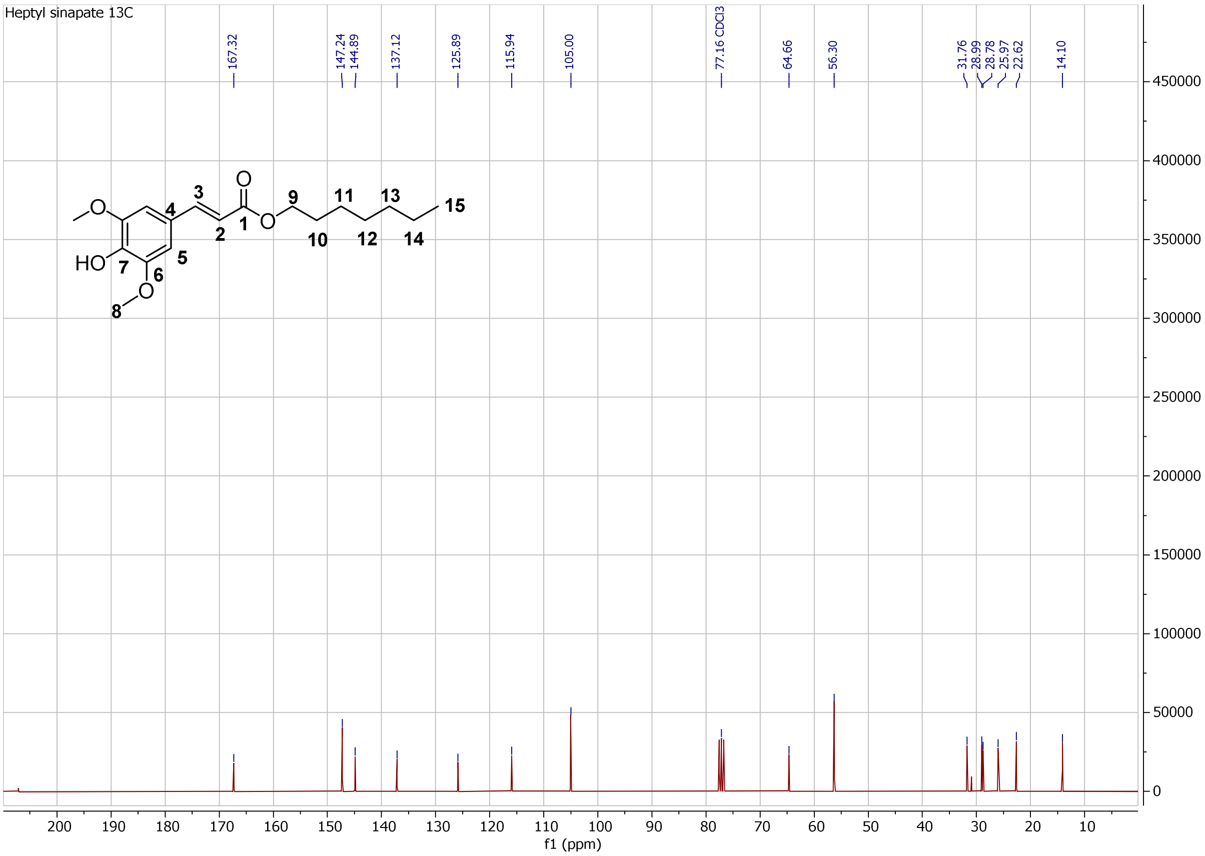

Syringol sinapate 1H

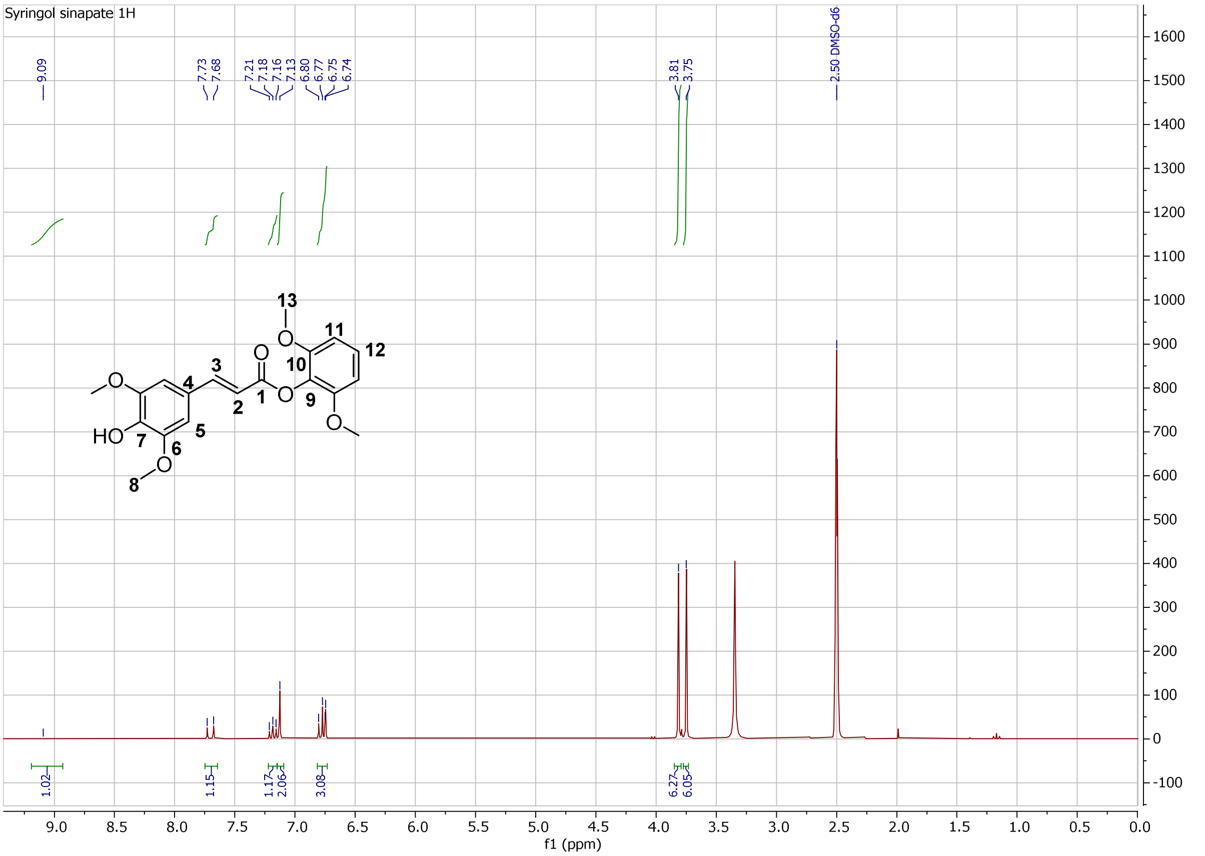

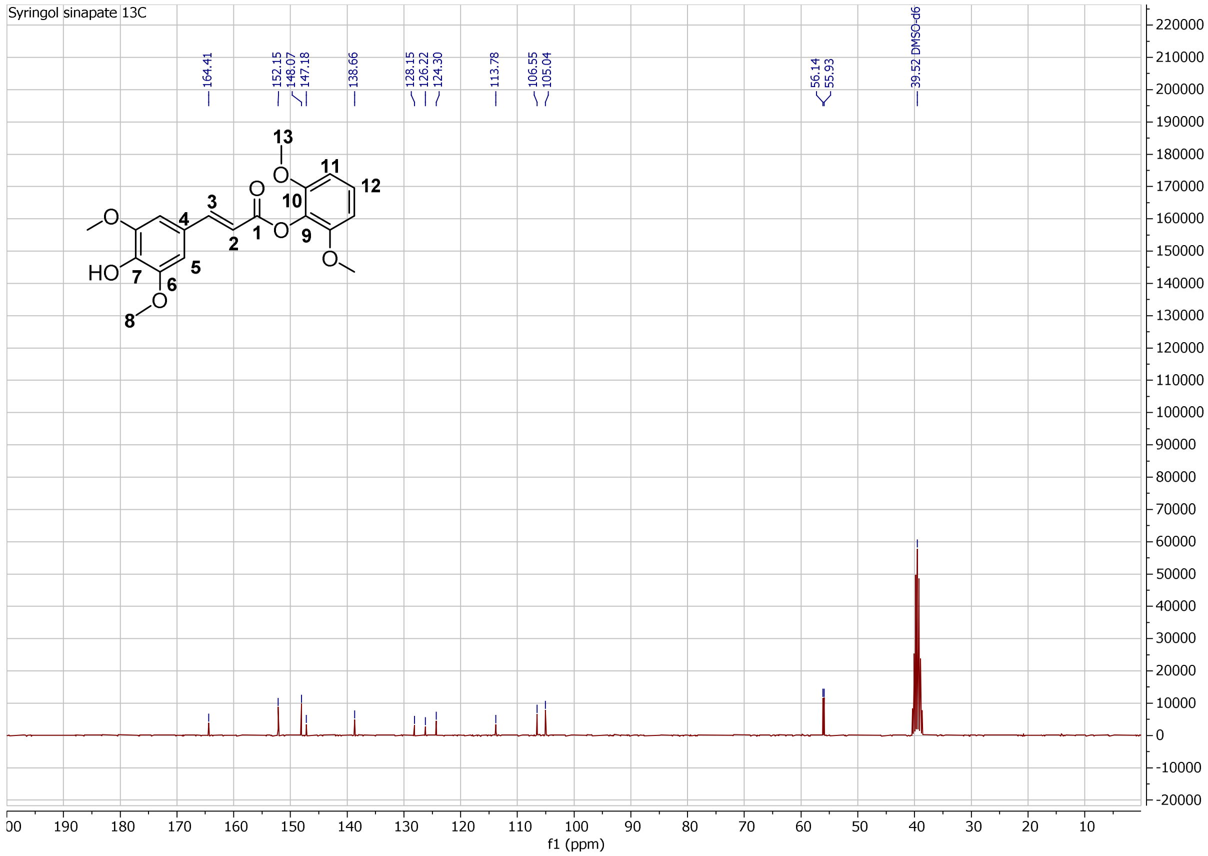
Syringol sinapate 13C
164.41
152.15
148.07
147.18
138.66
128.15
126.22
124.30
113.78
106.55
105.04
56.14
55.93
39.52 DMSO-d6
f1 (ppm)

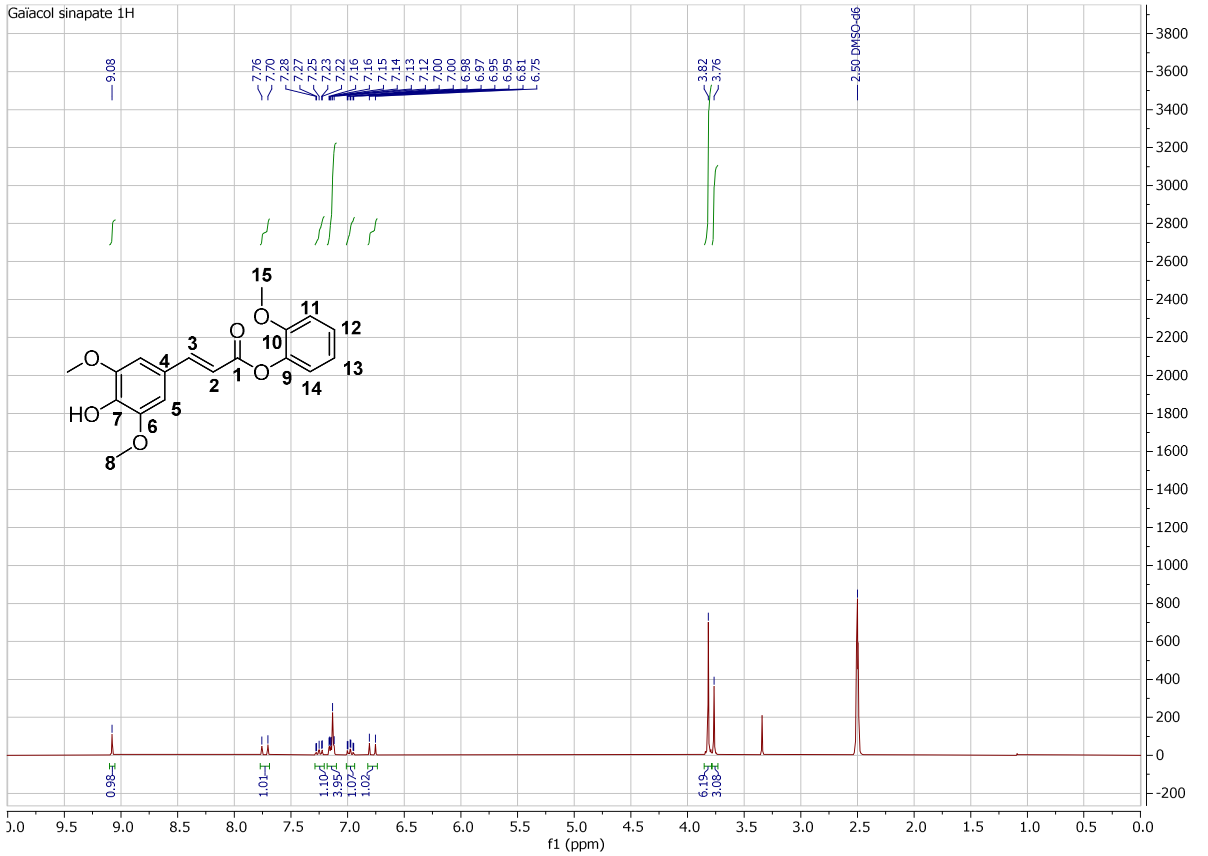
Gaïacol sinapate 1H
9.08
2.50 DMSO-d6
3.82
3.76
f1 (ppm)

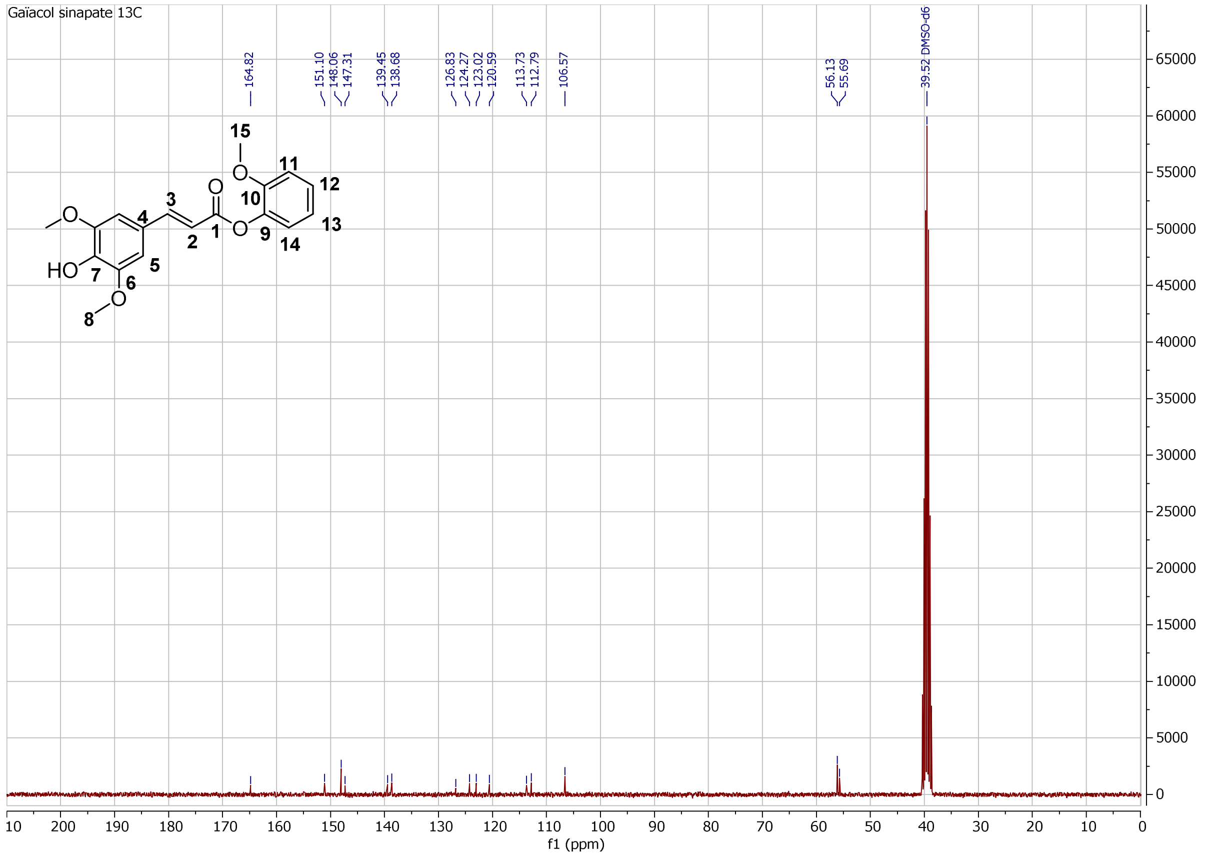

Gaïacol sinapate 13C
164.82
151.10
148.06
147.31
139.45
138.68
126.83
124.27
123.02
120.59
113.73
112.79
106.57
56.13
55.69
39.52 DMSO-d6
f1 (ppm)

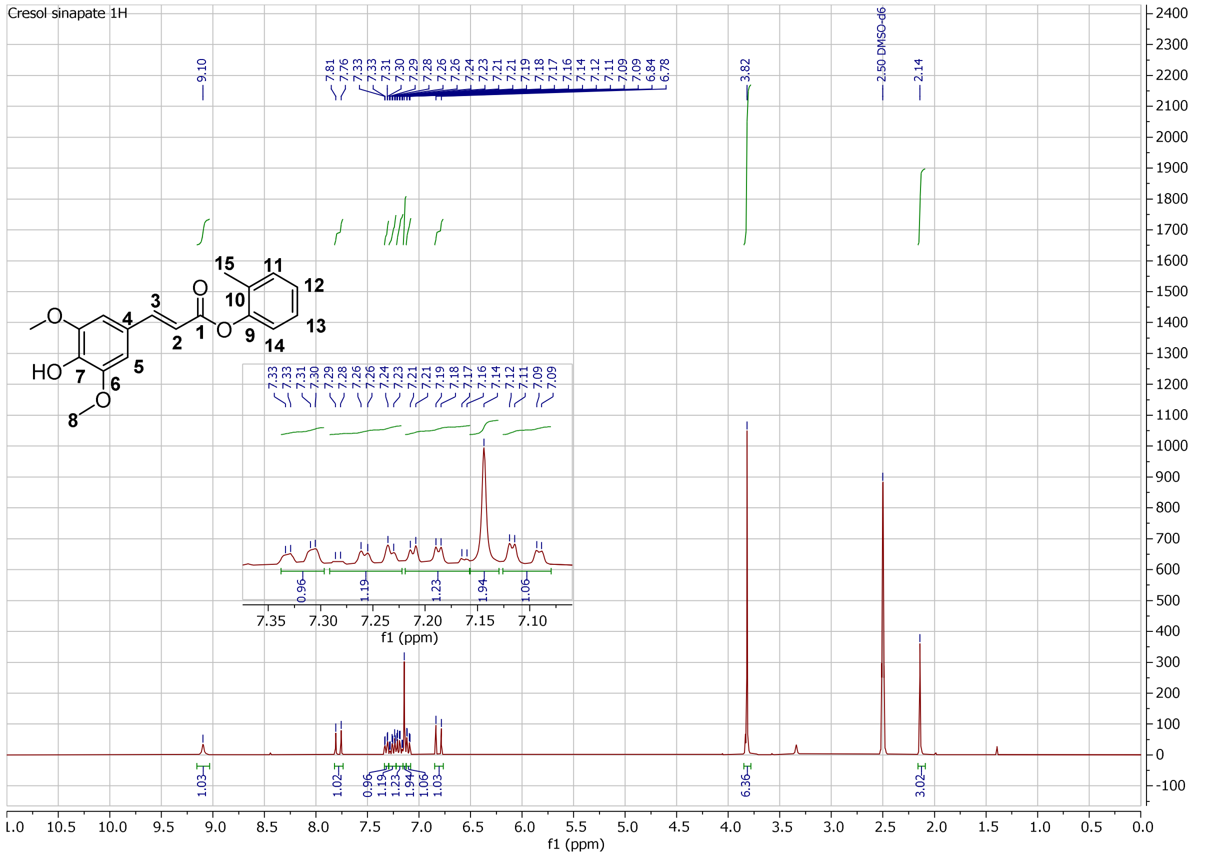

Cresol sinapate 1H
9.10
7.81
7.76
6.84
6.78
3.82
2.50 DMSO-d6
2.14
1.03
1.02
0.96
1.19
1.23
1.94
1.06
1.03
6.36
3.02
f1 (ppm)

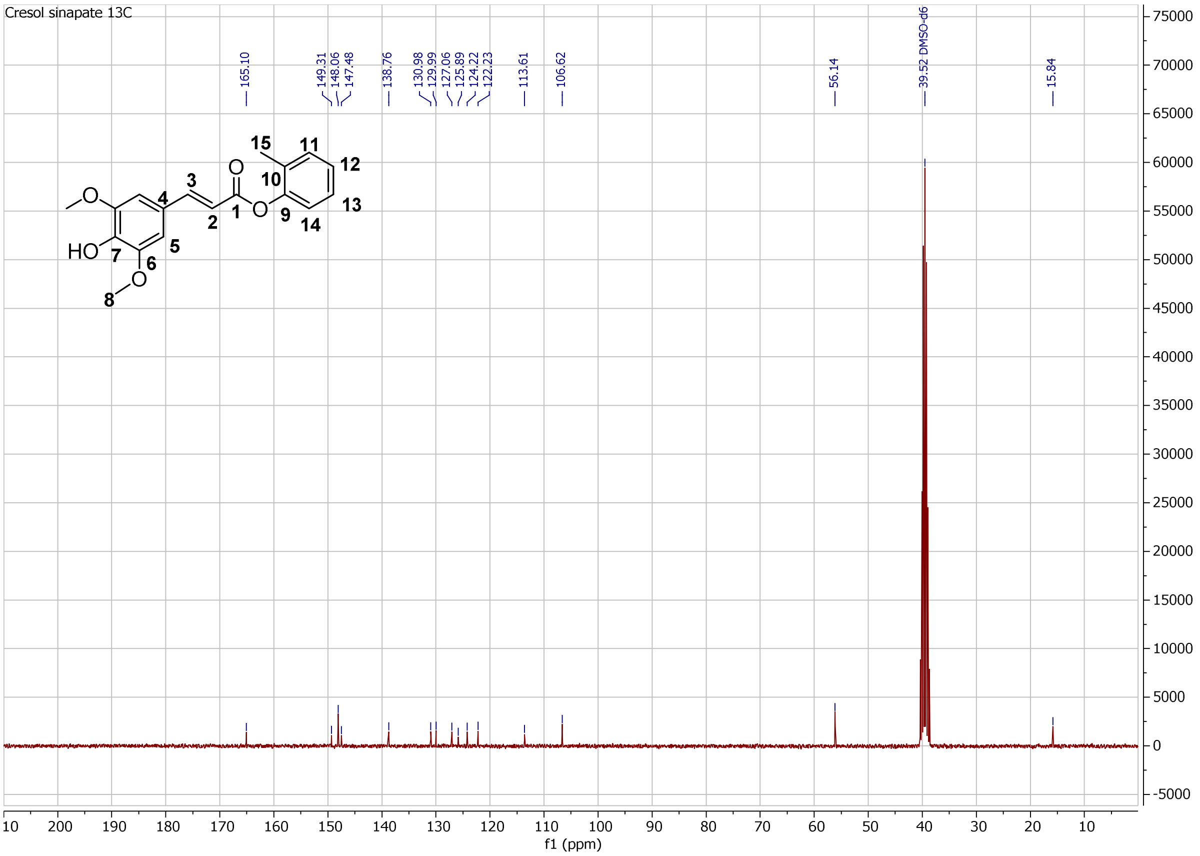
Cresol sinapate 13C
165.10
149.31
148.06
147.48
138.76
130.98
129.99
127.06
125.89
124.22
122.23
113.61
106.62
56.14
39.52 DMSO-d6
15.84
f1 (ppm)

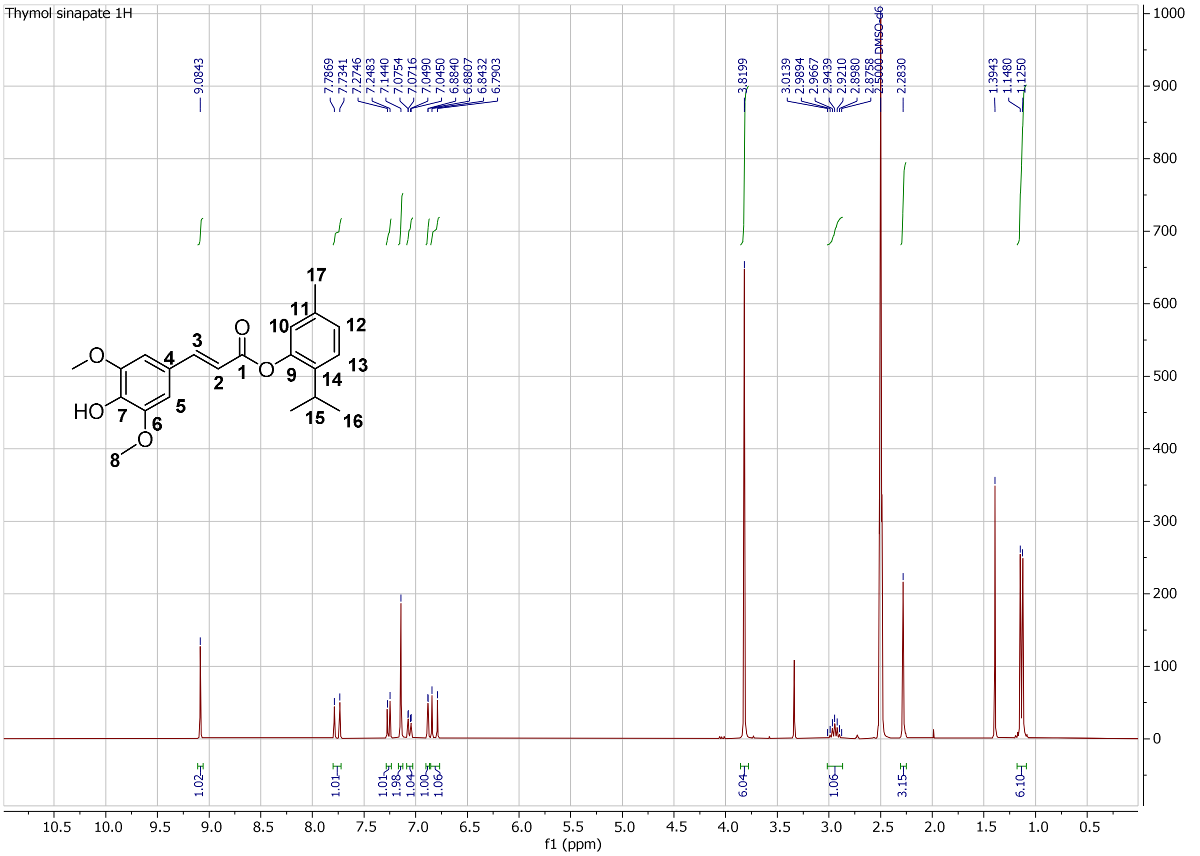
Thymol sinapate 1H
9.0843
7.7869
7.7341
7.2746
7.2483
7.1440
7.0754
7.0716
7.0490
7.0450
6.8840
6.8807
6.8432
6.7903
3.8199
3.0139
2.9894
2.9667
2.9439
2.9210
2.8980
2.8758
2.5000 DMSO-d6
2.2830
1.3943
1.1480
1.1250
1.02
1.01
1.01
1.98
1.04
1.00
1.06
6.04
1.06
3.15
6.10
f1 (ppm)

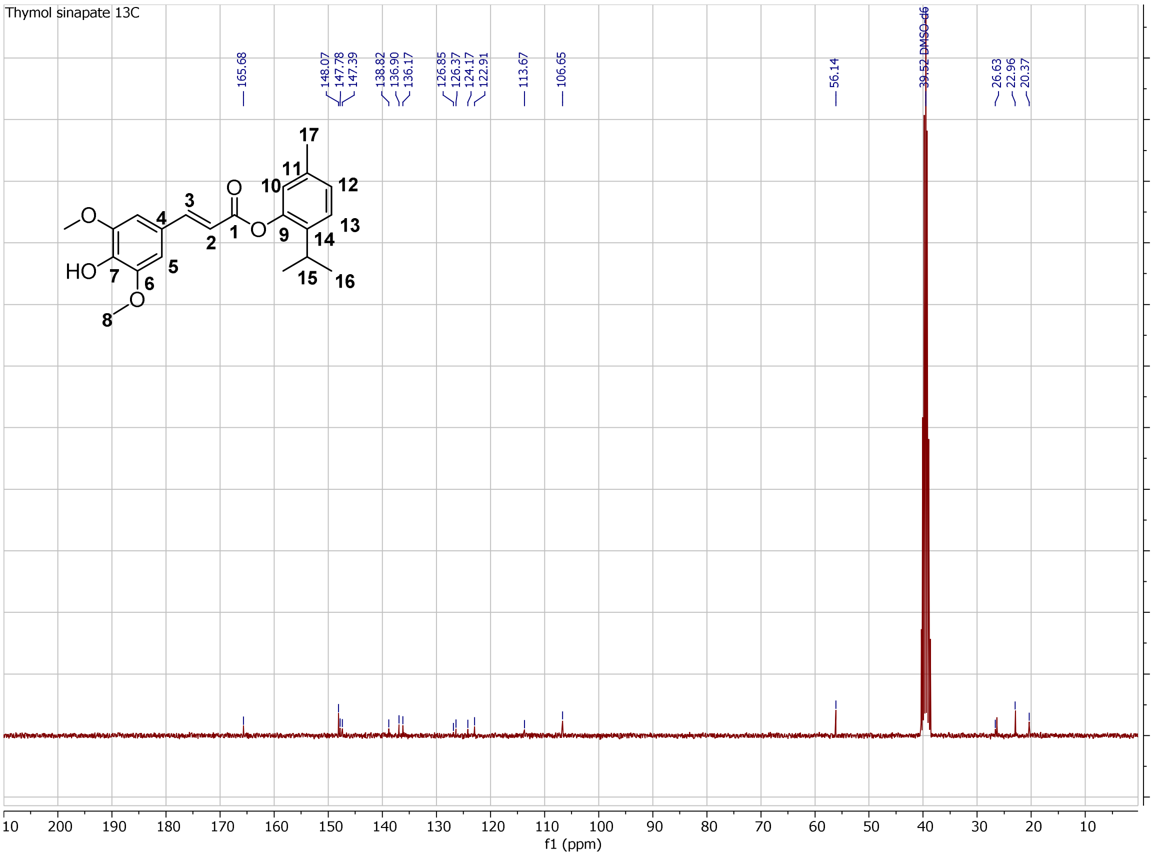
Thymol sinapate 13C
165.68
148.07
147.78
147.39
138.82
136.90
136.17
126.85
126.37
124.17
122.91
113.67
106.65
56.14
39.52 DMSO-d6
26.63
22.96
20.37
f1 (ppm)

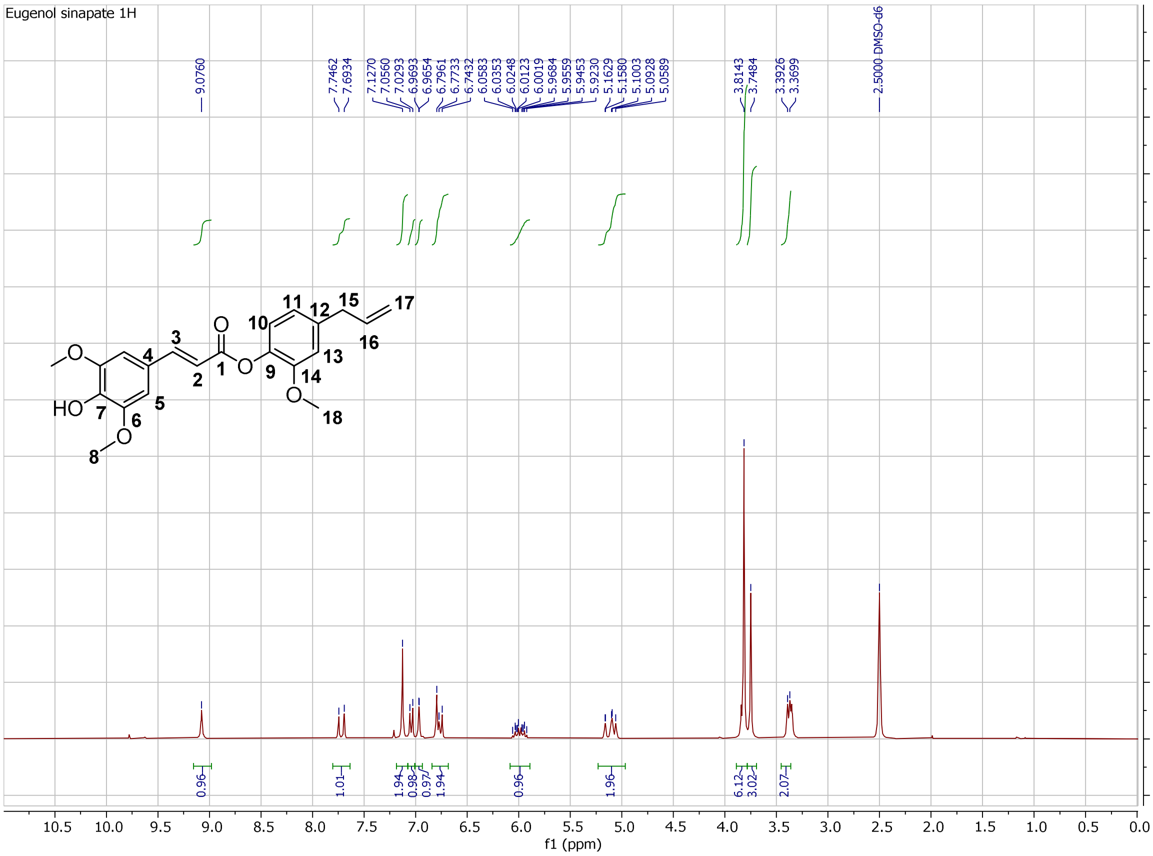
Eugenol sinapate 1H
9.0760
7.7462
7.6934
7.1270
7.0560
7.0293
6.9693
6.9654
6.7961
6.7733
6.7432
6.0583
6.0353
6.0248
6.0123
6.0019
5.9684
5.9559
5.9453
5.9230
5.1629
5.1580
5.1003
5.0928
5.0589
3.8143
3.7484
3.3926
3.3699
2.5000 DMSO-d6
0.96
1.01
1.94
0.98
0.97
1.94
0.96
1.96
6.12
3.02
2.07
f1 (ppm)

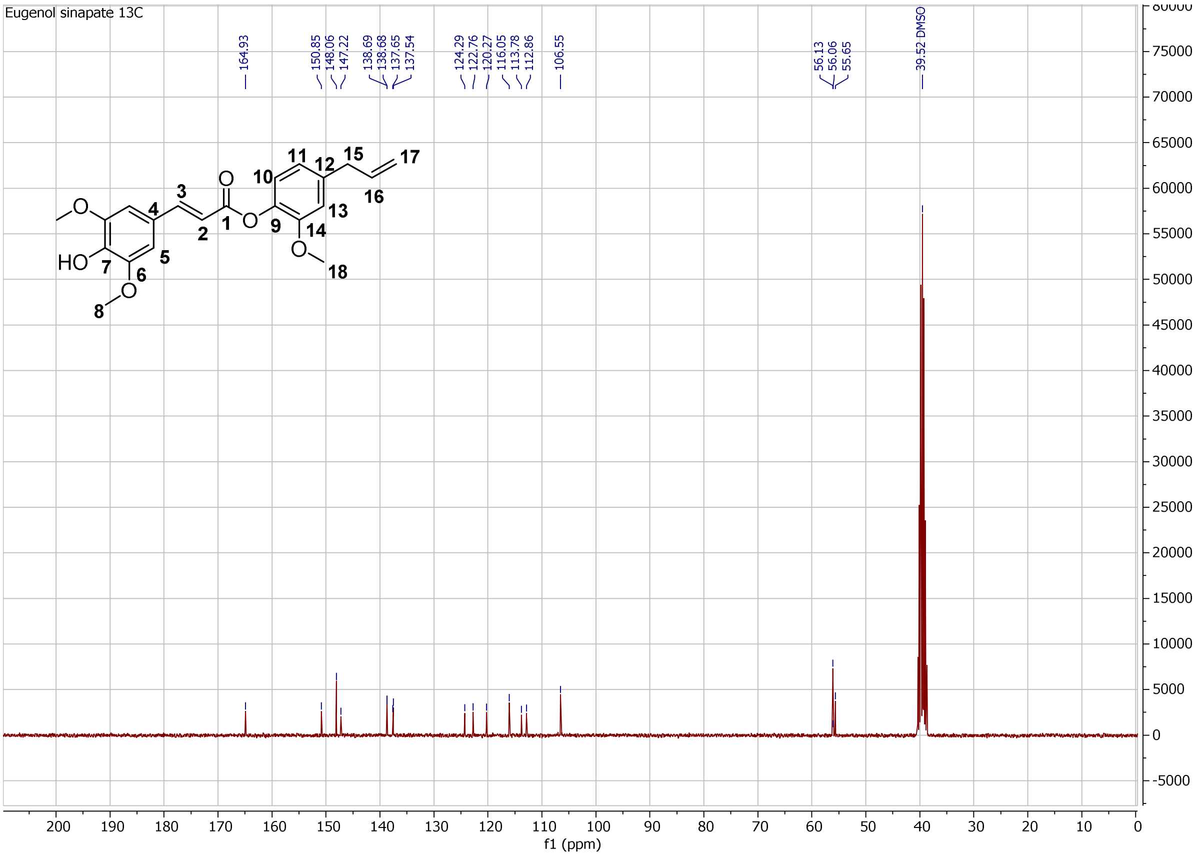

Eugenol sinapate 13C
164.93
150.85
148.06
147.22
138.69
138.68
137.65
137.54
124.29
122.76
120.27
116.05
113.78
112.86
106.55
56.13
56.06
55.65
39.52 DMSO
f1 (ppm)

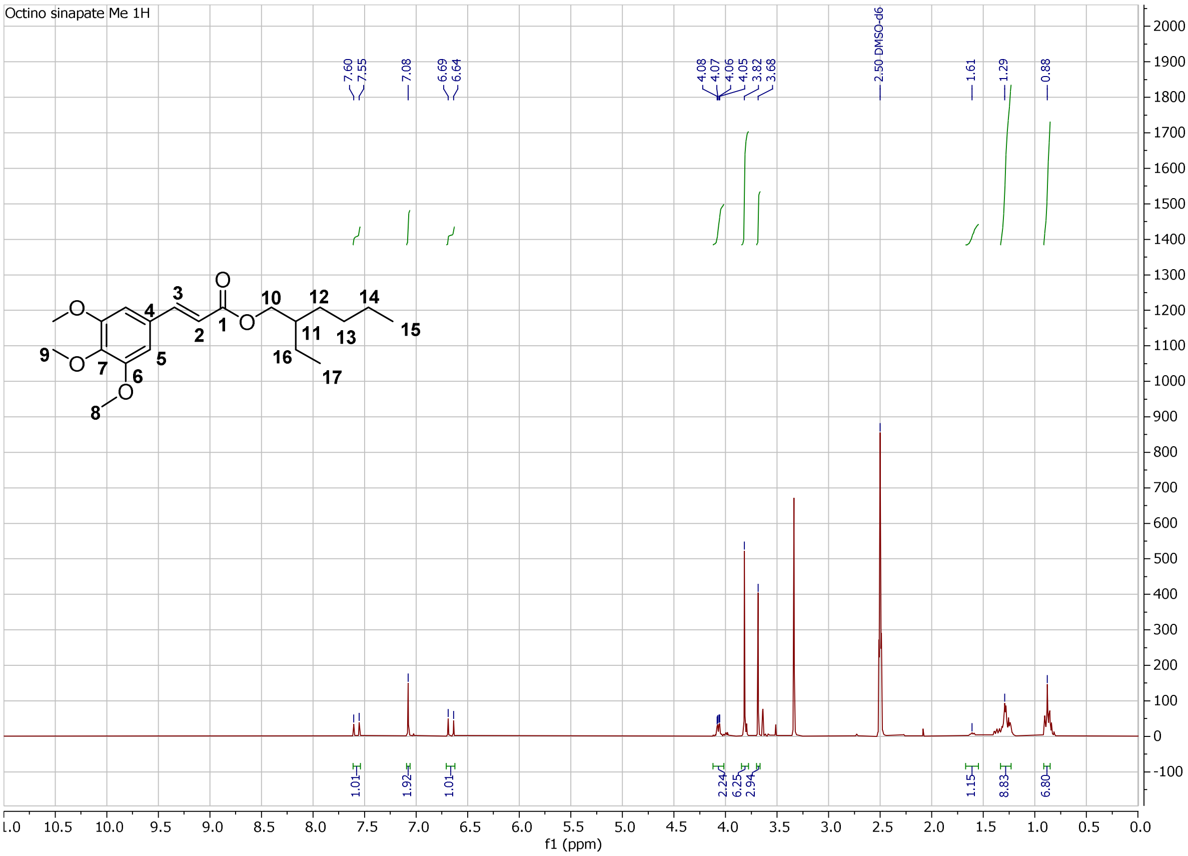

Octino sinapate Me 1H
7.60
7.55
7.08
6.69
6.64
4.08
4.07
4.06
4.05
3.82
3.68
2.50 DMSO-d6
1.61
1.29
0.88
1.01
1.92
1.01
2.24
6.25
2.94
1.15
8.83
6.80
f1 (ppm)

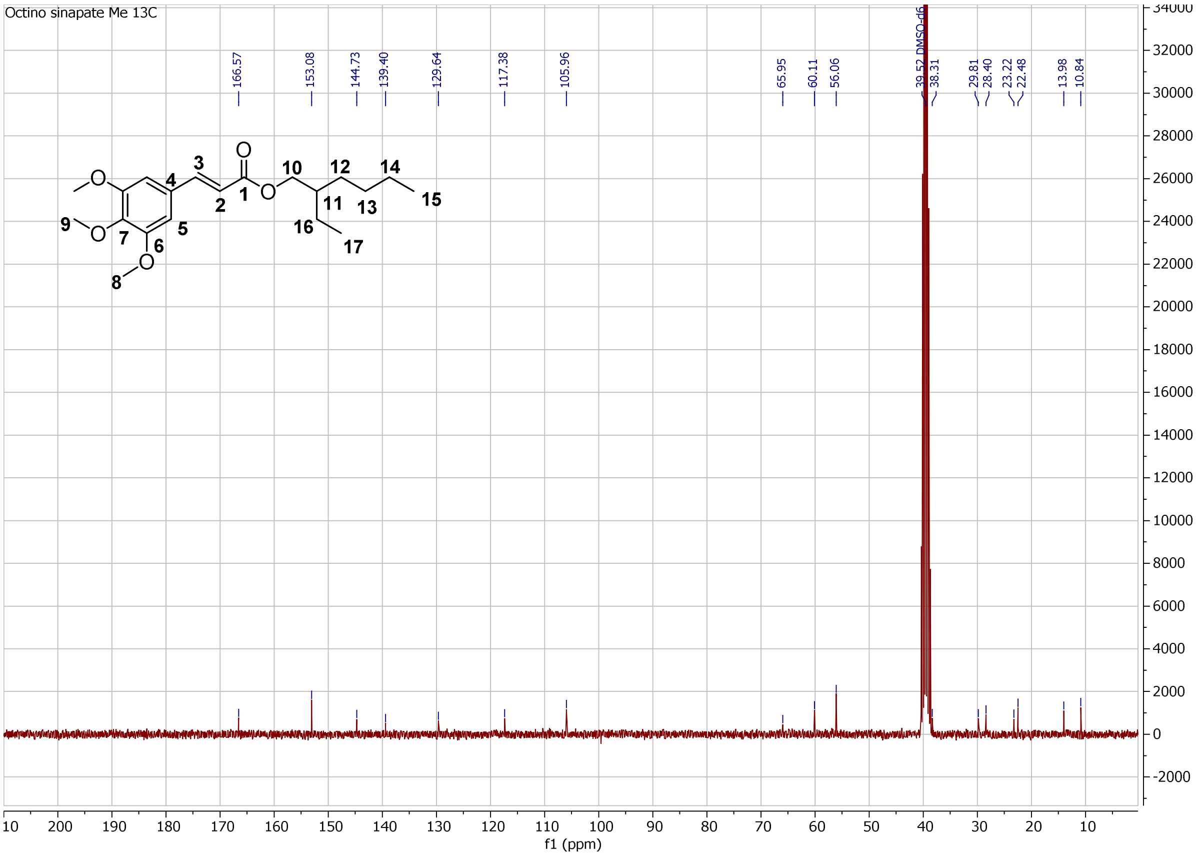
Octino sinapate Me 13C
166.57
153.08
144.73
139.40
129.64
117.38
105.96
65.95
60.11
56.06
39.52 DMSO-d6
38.31
29.81
28.40
23.22
22.48
13.98
10.84
f1 (ppm)

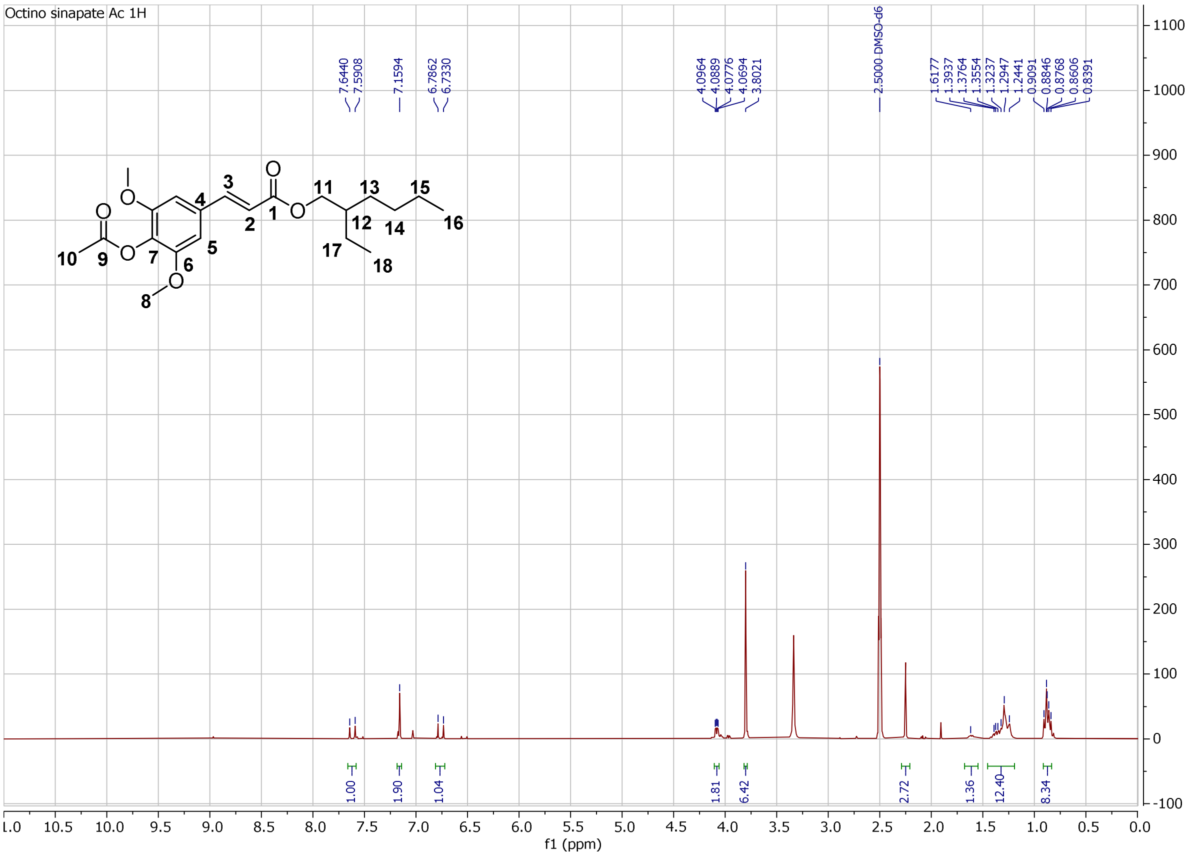
Octino sinapate Ac 1H
7.6440
7.5908
7.1594
6.7862
6.7330
4.0964
4.0889
4.0776
4.0694
3.8021
2.5000 DMSO-d6
1.6177
1.3937
1.3764
1.3554
1.3237
1.2947
1.2441
0.9091
0.8846
0.8768
0.8606
0.8391
1.00
1.90
1.04
1.81
6.42
2.72
1.36
12.40
8.34
f1 (ppm)

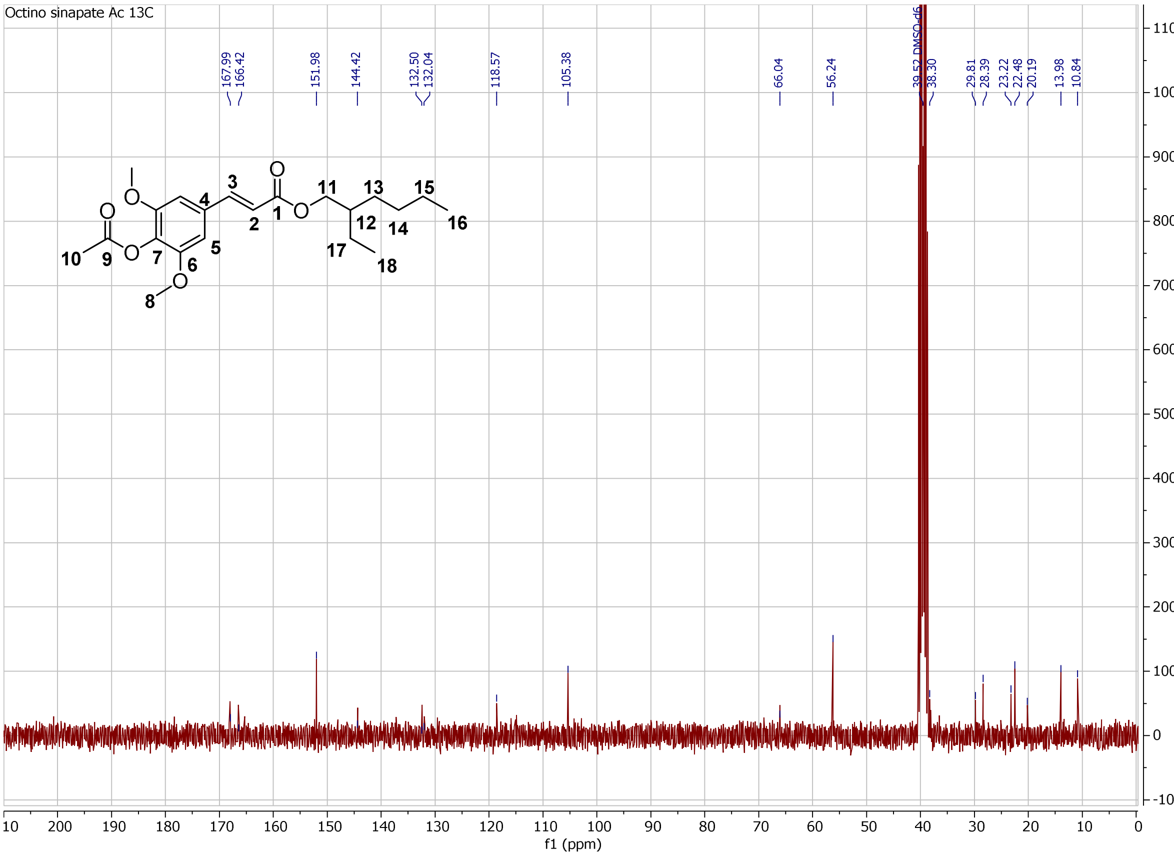
Octino sinapate Ac 13C
167.99
166.42
151.98
144.42
132.50
132.04
118.57
105.38
66.04
56.24
39.52 DMSO-d6
38.30
29.81
28.39
23.22
22.48
20.19
13.98
10.84
f1 (ppm)

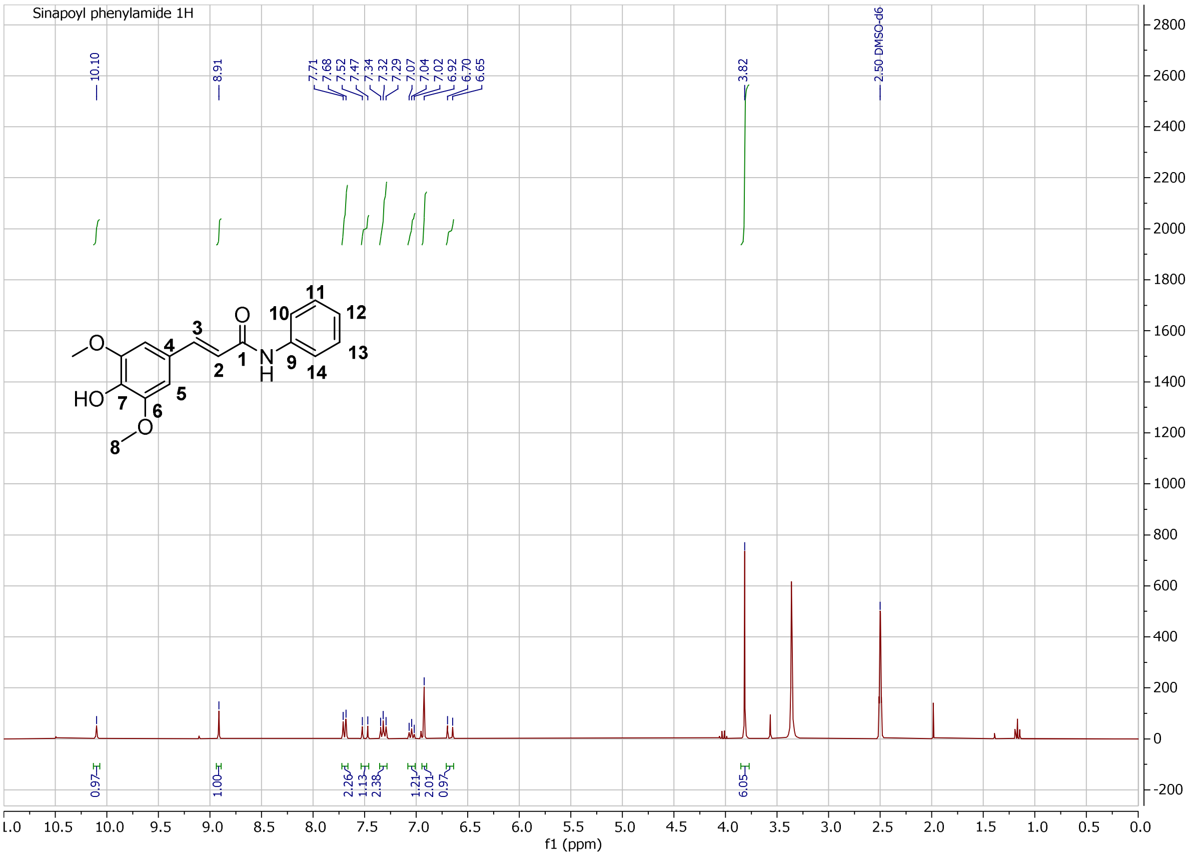
Sinapoyl phenylamide 1H
10.10
8.91
7.71
7.68
7.52
7.47
7.34
7.32
7.29
7.07
7.04
7.02
6.92
6.70
6.65
3.82
2.50 DMSO-d6
0.97
1.00
2.26
1.13
2.38
1.21
2.01
0.97
6.05
f1 (ppm)

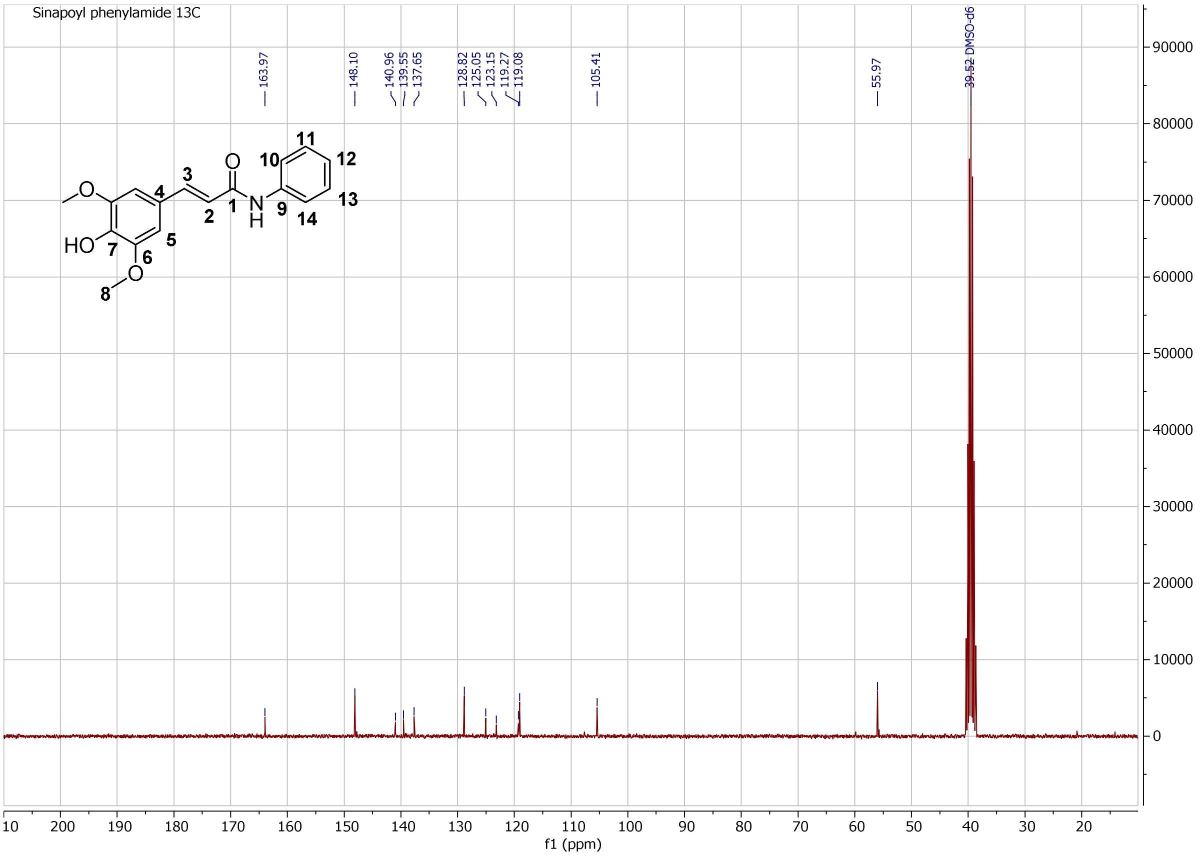
Sinapoyl phenylamide 13C
163.97
148.10
140.96
139.55
137.65
128.82
125.05
123.15
119.27
119.08
105.41
55.97
39.52 DMSO-d6
f1 (ppm)

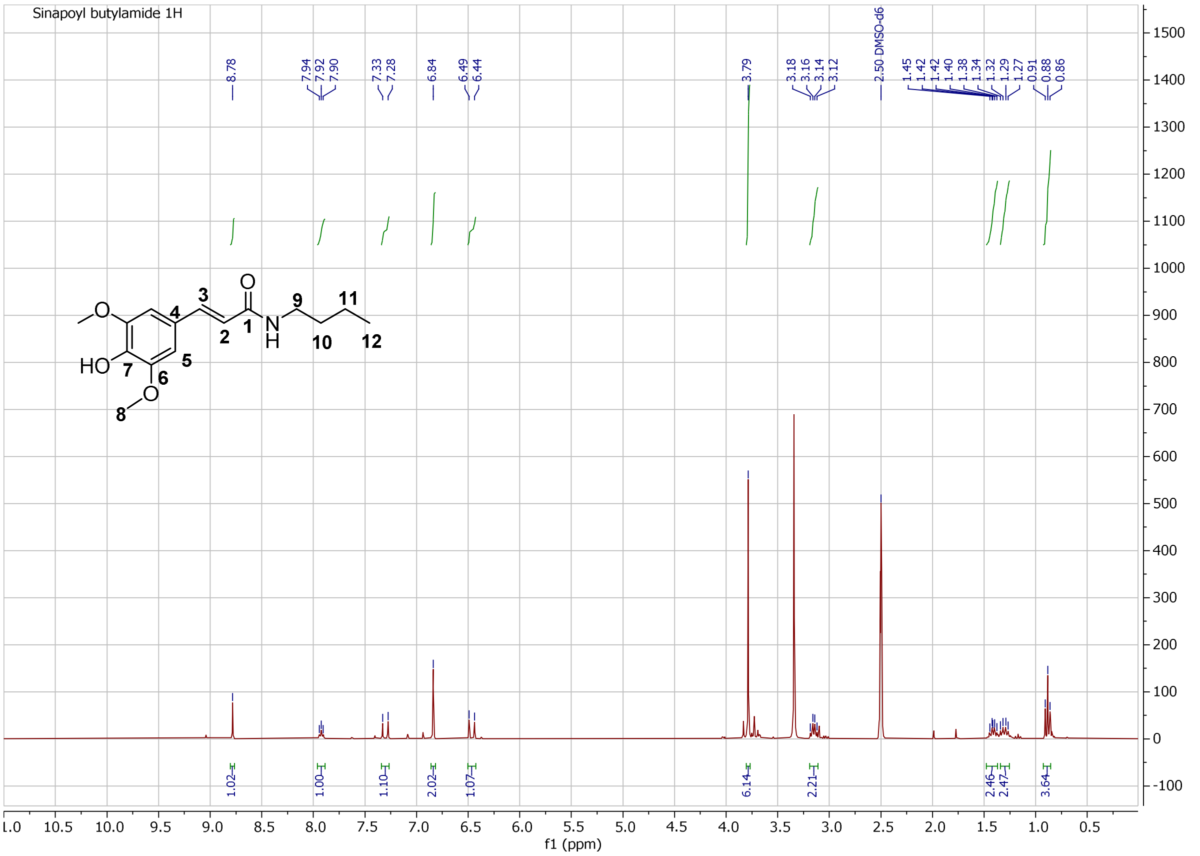
Sinapoyl butylamide 1H
8.78
7.94
7.92
7.90
7.33
7.28
6.84
6.49
6.44
3.79
3.18
3.16
3.14
3.12
2.50 DMSO-d6
1.45
1.42
1.42
1.40
1.38
1.34
1.32
1.29
1.27
0.91
0.88
0.86
1.02
1.00
1.10
2.02
1.07
6.14
2.21
2.46
2.47
3.64
f1 (ppm)

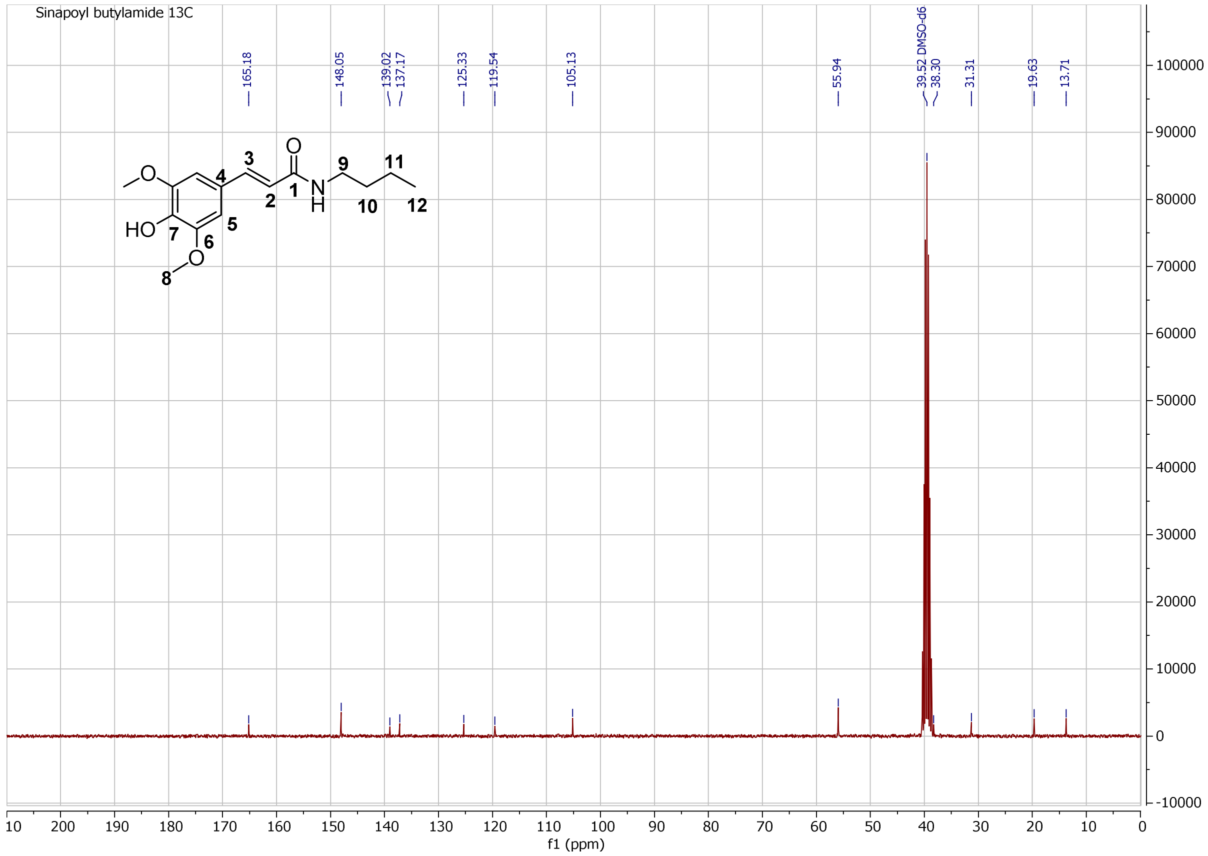
Sinapoyl butylamide 13C
165.18
148.05
139.02
137.17
125.33
119.54
105.13
55.94
39.52 DMSO-d6
38.30
31.31
19.63
13.71
f1 (ppm)

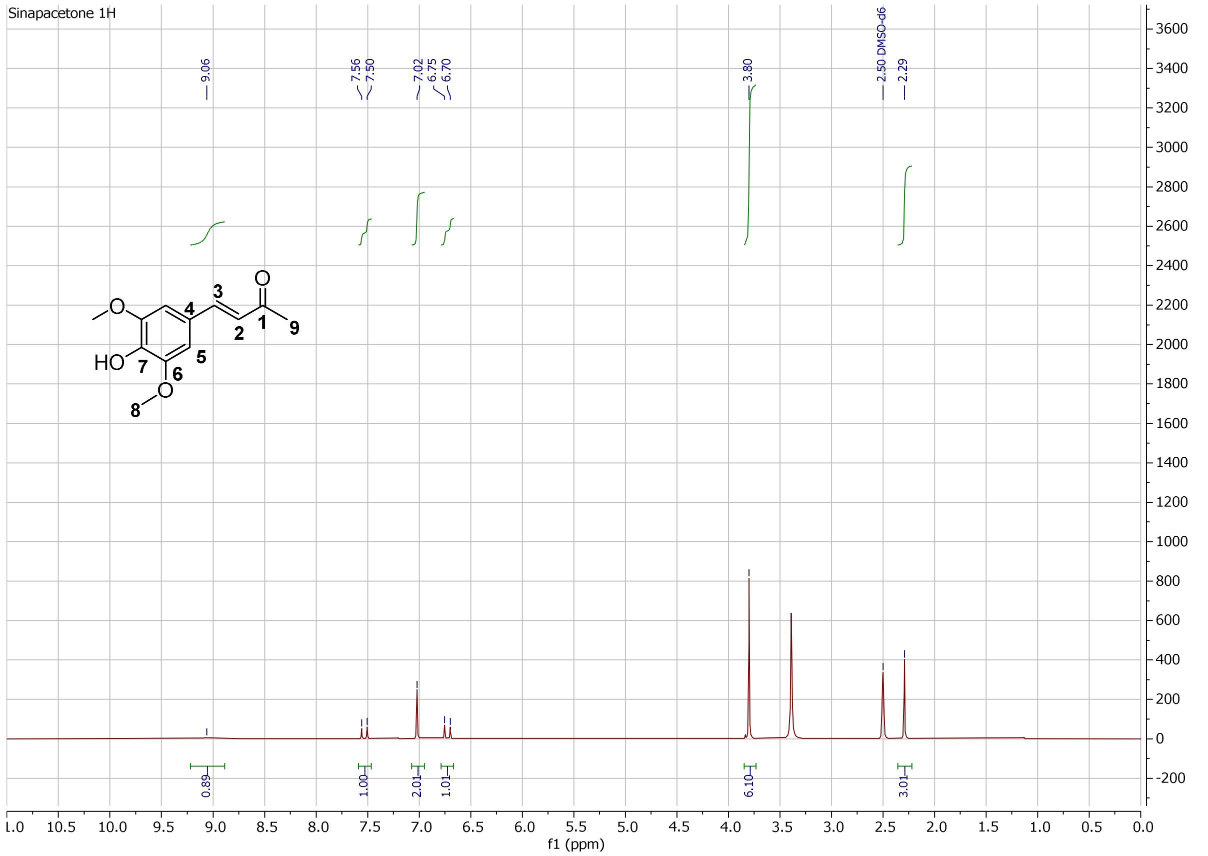
Sinapacetone 1H
9.06
7.56
7.50
7.02
6.75
6.70
3.80
2.50 DMSO-d6
2.29
0.89
1.00
2.01
1.01
6.10
3.01
f1 (ppm)

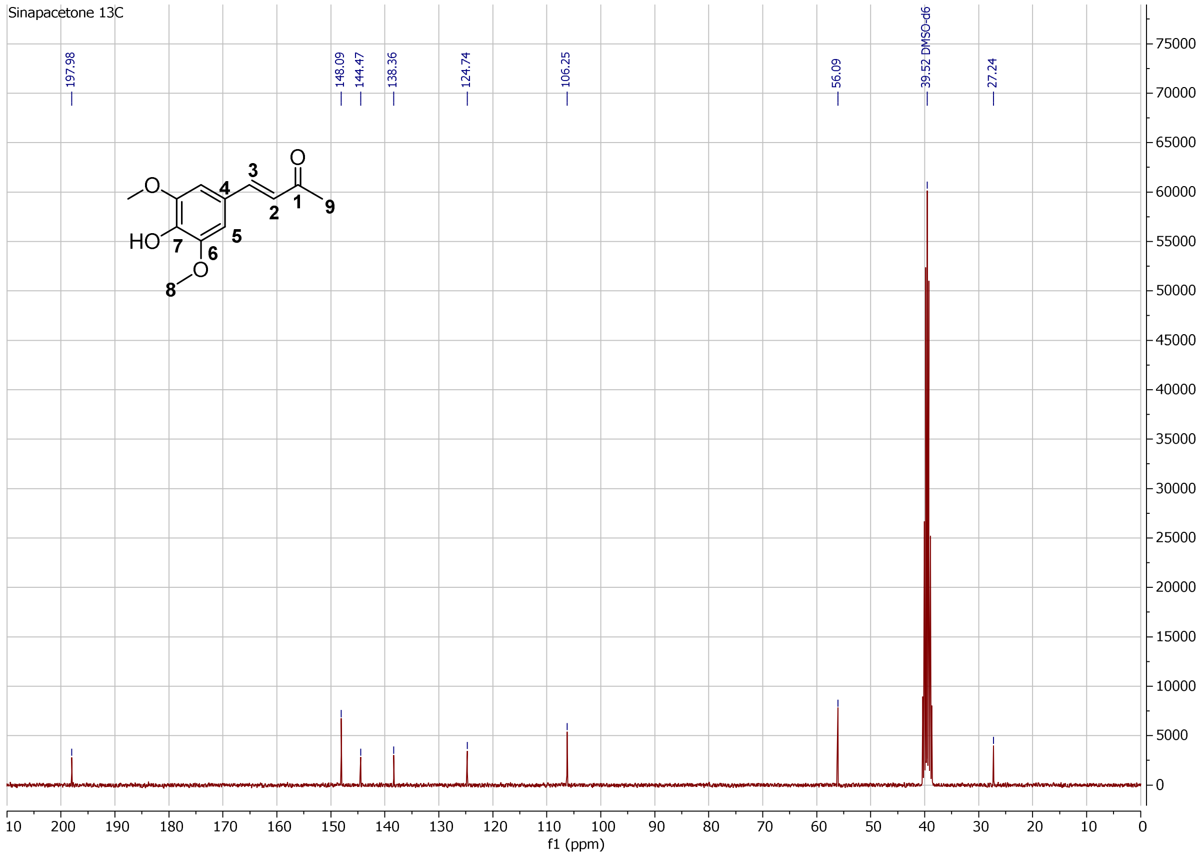
Sinapacetone 13C
197.98
148.09
144.47
138.36
124.74
106.25
56.09
39.52 DMSO-d6
27.24
75000
70000
65000
60000
55000
50000
45000
40000
35000
30000
25000
20000
15000
10000
5000
0
10
200
190
180
170
160
150
140
130
120
110
100
90
80
70
60
50
40
30
20
10
0
f1 (ppm)
O
HO
1
2
3
4
5
6
7
8
9
